# Spatially variable coevolution between a haemosporidian parasite and the MHC of a widely distributed passerine

**DOI:** 10.1002/ece3.1391

**Published:** 2015-02-06

**Authors:** Matthew R Jones, Zachary A Cheviron, Matthew D Carling

**Affiliations:** 1 Department of Zoology and Physiology, Berry Biodiversity Conservation Center, University of Wyoming1000 E. University Ave., Dept. 4304, Laramie, Wyoming, 82071; 2 Department of Animal Biology, School of Integrative Biology, University of Illinois Urbana-Champaign505 South Goodwin Ave., Urbana, Illinois, 61801

**Keywords:** Birds, disease biology, ecological genetics, immunogenetics, natural selection

## Abstract

The environment shapes host–parasite interactions, but how environmental variation affects the diversity and composition of parasite-defense genes of hosts is unresolved. In vertebrates, the highly variable major histocompatibility complex (MHC) gene family plays an essential role in the adaptive immune system by recognizing pathogen infection and initiating the cellular immune response. Investigating MHC-parasite associations across heterogeneous landscapes may elucidate the role of spatially fluctuating selection in the maintenance of high levels of genetic variation at the MHC. We studied patterns of association between an avian haemosporidian blood parasite and the MHC of rufous-collared sparrows (*Zonotrichia capensis*) that inhabit environments with widely varying haemosporidian infection prevalence in the Peruvian Andes. MHC diversity peaked in populations with high infection prevalence, although intra-individual MHC diversity was not associated with infection status. MHC nucleotide and protein sequences associated with infection absence tended to be rare, consistent with negative frequency-dependent selection. We found an MHC variant associated with a ∽26% decrease in infection probability at middle elevations (1501–3100 m) where prevalence was highest. Several other variants were associated with a significant increase in infection probability in low haemosporidian prevalence environments, which can be interpreted as susceptibility or quantitative resistance. Our study highlights important challenges in understanding MHC evolution in natural systems, but may point to a role of negative frequency-dependent selection and fluctuating spatial selection in the evolution of *Z. capensis*MHC.

## Introduction

Pathogen resistance in hosts involves an intricate interplay between host genotype, pathogen genotype, and the environment (Stevens 1960). The outcome of infection is often dependent on the interactions between host and pathogen genotypes resulting in a complex and highly contextual genetic architecture of parasite resistance (Lambrechts et al. 2006). Environmental heterogeneity may further moderate host–parasite interactions and quantitatively or qualitatively alter infection (reviewed in Thomas and Blanford 2003 and Wolinska and King 2009). For instance, the abiotic environment can affect multiple components of infection (e.g., host genetic resistance, postinfection mortality or recovery rate, and parasite virulence or abundance), resulting in spatially variable patterns of infection and parasite-mediated selection (Thomas and Blanford 2003; Mitchell et al. 2005; Lazzaro et al. 2008; Wolinska and King 2009; Mostowy and Engelstädter 2011). Thus, a particular host genotype may confer resistance to a parasite in one environment but have no effect on infection or even confer susceptibility to infection in another (Mitchell et al. 2005). While environmental variability often strongly mediates the outcome of infection, its consequences on host immunogenetic variation are less clear (Lazzaro and Little 2009; Wolinska and King 2009).

A substantial amount of research on the relationship between parasites and host immunogenetic variation has focused on the major histocompatibility complex (MHC), which is involved in the adaptive immune system and parasite resistance in vertebrates. The MHC gene family encodes cell surface proteins that bind a specific range of self-derived or pathogen-derived peptides (Matsumura et al. 1992). The MHC displays bound antigenic peptides to cytotoxic T-lymphocytes, which triggers the death of the infected cell. The specificity in binding affinity between individual MHC molecules and foreign peptides allows for associations between particular MHC alleles and disease prevalence in hosts (Westerdahl et al. 2005, 2012; Bonneaud et al. 2006; Kloch et al. 2010; Eizaguirre et al. 2012a), although the selective advantages of MHC alleles may be environmentally dependent (Bernatchez and Landry 2003; Loiseau et al. 2008, 2011). Levels of genetic variation in MHC are among the highest in vertebrate genomes (Robinson et al. 2013), which is generally attributed to parasite-driven balancing selection or MHC-based mate choice (Reusch et al. 2001; Bernatchez and Landry 2003). Examining the environmental context of MHC-parasite associations may facilitate inferences about whether parasite-mediated selection is operating on hosts and if selection on MHC is heterogeneous across environments (Bonneaud et al. 2006; Dionne et al. 2007; Ekblom et al. 2007; Kloch et al. 2010; Loiseau et al. 2011).

Here we investigated associations between the MHC of *Zonotrichia capensis* (rufous-collared sparrows) and haemosporidian parasite infection across replicate transects that span elevational and latitudinal gradients on the Pacific slope of the Peruvian Andes using previously published haemosporidian parasite prevalence (Jones et al. 2013) and MHC amplicon datasets (Jones et al. 2014). Spatial heterogeneity across mountain ranges in temperature, precipitation, and other abiotic and biotic variables provide a wide array of environmental contexts in which hosts and parasites interact. The geographic range of *Z. capenesis* is broad, extending throughout the Neotropics from sea level to roughly 5000 m elevation on the Pacific slope of the Andes mountain range.

**Figure 1:**
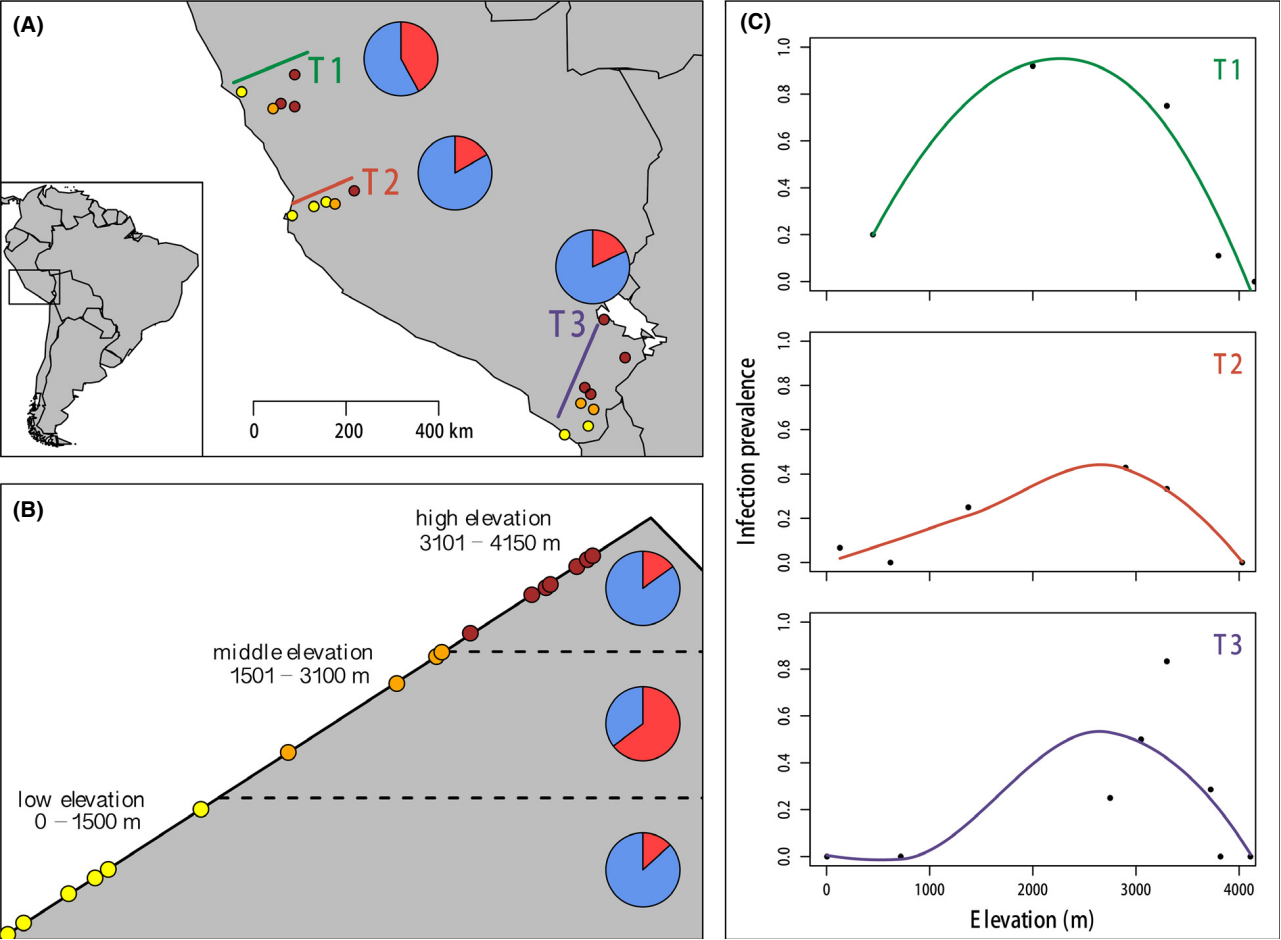
(A) Sampling localities shown as yellow (low elevation), orange (middle elevation), and red (high elevation) points across three transects (T1–T3). The pie graphs show the proportion of infected individuals on each transect (red fraction). (B) Elevational distributions of sampling localities across all transects with the proportion of infected individuals in pie graph. (C) Parasite infection prevalence across elevation for each replicate transect (adapted from Jones et al. 2013). The regression lines are fit using local polynomial regression fitting.

A previous survey of avian malaria and other haemosporidian parasites in Pacific slope *Z. capensis* revealed substantial spatial heterogeneity in infection prevalence across both elevation and latitude (Jones et al. 2013; Fig.1). Individuals that inhabited environments with the highest prevalence of a *Haemoproteus* parasite (*Haemoproteus* sp. KC480265) tended to also have a higher level of MHC diversity (Jones et al. 2014) compared to individuals from low prevalence environments, although it is unclear whether parasite-mediated selection drove this pattern and, if so, what mode of selection was responsible. For instance, heterozygote advantage, negative frequency-dependent selection, and fluctuating selection are all commonly invoked to explain patterns of high MHC variation and its linkage to disease (Spurgin and Richardson 2010). Heterozygote advantage is a form of selection where heterozygous individuals or individuals with high MHC allelic diversity are capable of resistance against a wide array of parasites (Hughes and Nei 1988; Reusch et al. 2001; Penn et al. 2002; Wegner et al. 2003). Under negative frequency-dependent selection, rare MHC variants are favored in host populations because parasites are expected to adapt to common MHC variants (Takahata and Nei 1990). Under fluctuating selection, MHC alleles are selected at different times or environments because spatial or temporal heterogeneity in parasite diversity, virulence, or abundance alters the strength or specificity of directional parasite-mediated selection (Hedrick 2002; Loiseau et al. 2011). Mathematical models suggest that heterozygote advantage is unable to maintain high levels of MHC diversity alone (De Boer et al. 2004); however, both negative frequency-dependent selection and fluctuating selection are capable of maintaining MHC genetic diversity within populations even in the absence of other parasite-mediated selection mechanisms (Hedrick 2002; Borghans et al. 2004; Charbonnel and Pemberton 2005).

Distinguishing between mechanisms of parasite-mediated selection in wild populations is difficult because they are not mutually exclusive and can produce similar signatures of selection (Spurgin and Richardson 2010). Teasing apart negative frequency-dependent selection and fluctuating selection is a major challenge because associations between specific MHC variants and infection status are consistent with both processes. A disease-association study that integrates MHC genealogical information may help to distinguish between these two hypotheses by revealing the origins and relative ages of specific variants associated with infection (Spurgin and Richardson 2010). To infer whether *Haemoproteus*-driven selection influences the spatial variation in MHC diversity in *Z. capensis* we tested whether overall MHC diversity or specific MHC alleles are associated with parasite infection status. Furthermore we tested whether MHC variants associated with lack of infection are rare or young in the population (suggestive of negative frequency-dependent selection) and whether different MHC variants are associated with *Haemoproteus* infection across different environments (suggestive of fluctuating spatial selection).

## Methods

### Study population

We used *Haemoproteus* prevalence and MHC datasets of 184 *Z. capensis* individuals collected along three transects (T1–T3) that span elevational and latitudinal gradients on the Pacific slope of the Peruvian Andes from 2004 to 2007 (Fig.1; Jones et al. 2013, 2014). Populations of *Z. capensis* along the Pacific slope of the Andes are nonmigratory, although information on local movement patterns is lacking (Schulenberg et al. 2007; Cheviron and Brumfield 2009). Along elevational gradients, mean elevation gain was approximately 3900 m over a mean linear distance of approximately 161 km. Latitudinal gradients spanned relatively small elevational distances over large latitudinal distances (mean elevation change = +174 m, mean linear distance = 509 km). Individuals were either collected in mist nests and euthanized or shot (see Table S1 for specimen data). Total genomic DNA was extracted from frozen-preserved pectoral muscle using DNeasy Blood and Tissue Kits (Qiagen, Valencia, CA).

### Infection screening

Avian haemosporidians (*Plasmodium*,*Haemoproteus*, and *Leucocytozoon*) are a diverse group of parasites that, along with their vectors, show strong spatial heterogeneity in their virulence and abundance driven primarily by temperature and precipitation regimes (Valkiūnas 2005; LaPointe et al. 2010, 2012). The acute stage of haemosporidian infection, during which parasites reside in the blood, is brief and associated with reduced host survival in some species (Atkinson et al. 2000, 2001; Valkiūnas 2005). Hosts surviving acute infection may develop chronic infection where parasites occur at low densities within hosts (Valkiūnas 2005; LaPointe et al. 2012). Symptoms of chronic haemosporidian infections may include reduced reproductive output (Richner et al. 1995; Oppliger et al. 1996; Merino et al. 2000; Marzal et al. 2005; Knowles et al. 2010), decreased body condition (Dawson and Bortolotti 2000; Merino et al. 2000), and reduced survival (Martínez-de la Puente et al. 2010).

Jones et al. (2013) described patterns of haemosporidian infection prevalence in *Z. capensis*. We used the dataset published in Jones et al. (2013) to investigate associations between haemosporidian infection and MHC. Details of the protocol used to identify infections can be found therein. Briefly, to screen for infection status (infected or uninfected), we used a nested PCR assay to amplify *cyt* b of *Haemoproteus* and *Plasmodium* parasites from host muscle tissue (Waldenström et al. 2004). Both acute and chronic infections can be identified in host muscle tissue because megalomeronts, apicomplexan parasites in the process of asexual reproduction, develop in host muscle tissue (Valkiūnas 2005). Thus, while transmission dynamics may change seasonally (Valkiūnas 2005), infections are detectable year-round. Genetic data were archived in GenBank (accession # KC480265–KC480268).

Extending the work of Jones et al. (2013) we examined spatial patterns of infection prevalence with a Bayesian model with a binomial likelihood distribution and flat beta prior distribution (*α* = 1, *β* = 1) implemented in R with the *qbeta* and *pbeta* functions (R Development Core Team 2013). We estimated infection prevalence and calculated 95% credible intervals of infection prevalence for individuals grouped according to their latitudinal (T1, T2, or T3) and elevational zone (low: 0–1500 m, middle: 1501–3100 m, high: 3101–4150 m).

### Parasite prevalence ecological modeling

Under fluctuating selection, abiotic conditions are expected to strongly influence parasite prevalence or virulence and, as a consequence, parasite-mediated selection on hosts (Spurgin and Richardson 2010). We inferred important environmental variables correlated with haemosporidian infection prevalence using Random Forests implemented in the package *randomForest* (Liaw and Wiener 2002) in R (R Development Core Team 2013). Ecological modeling is challenging because ecological datasets often contain spatial autocorrelation, high dimensionality, and complex and nonlinear interactions between predictor variables (Breiman 2001a). Random Forests is a highly accurate nonparametric classification and regression tree approach, which accounts for multicollinearity, nonlinear relationships, and complex variable interactions (Breiman 2001b; Cutler et al. 2007; Evans and Cushman 2009). Classification trees (used with a categorical response variable) or regression trees (used with a continuous response variable) assign observations into classes of predictor variable space by recursively partitioning the data in a binary fashion into increasingly homogenous variable space (Breiman et al. 1984). Random Forests combine predictions from many classification or regression trees fit to a subsample (bootstrap sample) of the data. We used Random Forests to create regression trees with parasite infection prevalence at each collection site as the response variable. We used 19 bioclimatic variables (worldclim.org, 0.5 km resolution) related to temperature and precipitation as predictor variables because previous research suggests they are likely variables of importance in driving spatial distributions of avian haemosporidian infection prevalence (Valkiūnas 2005; LaPointe et al. 2010, 2012; Sehgal et al. 2011). To enhance the predictive power of the model, we assessed multicollinearity between environmental variables and removed collinear variables (*P *<* *0.1; Murphy et al. 2010). We ran 10,000 bootstrap replicates with replacement using a 35% bootstrap data withhold (out-of-bag or OOB sample) and used a model improvement ratio to select the optimal model (Murphy et al. 2010).

### MHC sequencing and genealogy

Most passerine MHC is highly complex as a result of gene duplication producing a large number of functional and nonfunctional loci (Hess et al. 2000; Hess and Edwards 2002; Balakrishnan et al. 2010). High-throughput sequencing offers the advantage of obtaining high coverage of MHC sequences across duplicated loci at the cost of being unable to assign alleles to specific loci. Jones et al. (2014) used 454 sequencing to generate a dataset of MHC class I exon 3 amplicons for *Z. capensis* individuals with infection presence or absence data. MHC class I exon 3 plays an important role in defense against intracellular pathogens, such as haemosporidians, because it encodes a substantial portion of the peptide-binding region (Bjorkman et al. 1987; Bonneaud et al. 2006; Alcaide et al. 2009; Loiseau et al. 2011; Wang et al. 2014). Multiplexed MHC amplicon libraries were generated using PCR and sequenced on a Roche 454 GS FLX + platform and MHC nucleotide sequences were culled with a stringent verification procedure (described in Jones et al. 2014). Nucleotide sequences representing putatively functional alleles were archived in GenBank (accession # KF433977–KF434074). Here we refer to unique nucleotide sequences that passed the variant culling criteria as MHC alleles. We refer to unique translated proteins as MHC proteins. Jones et al. (2014) produced a genealogy of putatively functional MHC alleles using MrBayes v.3.2.1 (Ronquist and Huelsenbeck 2003) with a *Carpodacus erythrinus* out-group.

### MHC supertype determination

MHC alleles or proteins are often defined as the functional unit of disease resistance; however, it is the peptide-binding region (PBR) of alleles that interacts with pathogen-derived peptides to produce an immune response. MHC types (hereafter “supertypes”) classified by variation within the PBR of MHC alleles may provide a more accurate predictor of disease resistance compared to MHC alleles or proteins (Doytchinova and Flower 2005).

The closest related species to *Z. capensis* with an annotated MHC PBR is *Gallus gallus*, which is 80–100 million years diverged (Shetty et al. 1999). MHC structure in passerines is highly complex compared to *G. gallus* and has undergone substantial rearrangement (Balakrishnan et al. 2010). Therefore, sequence homology may provide a poor estimate of the *Z. capensis* PBR. On the other hand, positively selected sites in the MHC may provide an accurate alternative to identify functionally important variation (Ellison et al. 2012; Sepil et al. 2012, 2013). To reveal codon positions under positive selection and subsequently classify MHC supertypes, Jones et al. (2014) tested for historical selection using the ratio of nonsynonymous mutations to synonymous mutations (*d*_N_/*d*_S_) in the CodeML module of PAML (Yang 2007) using a “sites” model, which allows *d*_*N*_*/d*_*S*_ to vary across amino acid positions, and a likelihood ratio and an empirical Bayes procedure to detect positively selected sites.

Biochemical properties of positively selected sites were quantified according to their hydrophobicity, steric bulk, polarity, and electronic effects (Doytchinova and Flower 2005; Jones et al. 2014). MHC alleles were then categorized into “supertypes” based on biochemical similarities of amino acids at positively selected sites using a K-means clustering algorithm in the R package *adegenet* (Jombart 2008; Jombart et al. 2010).

### Population divergence analyses

To examine patterns of MHC divergence between populations we tested for isolation-by-distance using a Mantel test in the R package *ecodist* (Goslee and Urban 2007) with distance matrices calculated using the package *gstudio* (Dyer 2009). We partitioned the data by sampling site and used a Euclidean distance metric to calculate distance between population allele frequency vectors. To create a genetic distance matrix for the MHC dataset we coded MHC alleles as “loci” and the presence or absence of the allele as the “allele state”.

### Statistical analyses

We tested for associations between parasite infection status and the total number of alleles per individual, the number of rare alleles (allele frequency <5%) per individual, the number of unique proteins per individual, and the number of unique supertypes per individual. The number of MHC alleles per individual is strongly affected by elevational zone (Jones et al. 2014), so we performed an analysis of covariance (ANCOVA) to control of the effect of elevational zone on MHC diversity. A significant association between infection status and MHC diversity across paralogous loci is consistent with parasite-mediated selection; however, the specific selection mechanism cannot be inferred from this observation alone (Spurgin and Richardson 2010).

To test for correlations between particular MHC variants (alleles, proteins, and supertypes) and parasite infection status we used a Bayesian variable selection regression (BVSR) model carried out in the program piMASS (Guan and Stephens 2011). BVSR models applied to genotype–phenotype association studies use the phenotype as the regression response and genetic variants as regression covariates to evaluate variants or genomic regions that are strongly associated with a particular phenotype. piMASS is generally used for genome-wide association studies with continuous response variables, but is also appropriate for an array of association studies, including candidate gene studies (Linnen et al. 2013) and studies with binary phenotypes (Guan and Stephens 2011). piMASS evaluates multiple predictor models, which incorporate different subsets of genetic variants and produces a model average of parameter estimates. MHC variants strongly associated with infection status are identified by the posterior distribution of *γ*, or the posterior inclusion probability (*PIP*). An elevated *PIP* indicates a strong association with infection status. An estimate of the effect size of variants on a phenotype is given by *β* (negative *β* associated with infection absence and positive *β* associated with infection presence). Additional parameters that are estimated in the model include proportion of variance explained by a genetic variant (*PVE*) and the average effect of a genetic variant on a phenotype (*σ*_*SNP*_). Multi-SNP approaches, such as BVSR, generally outperform single-SNP approaches in detecting true causal variants even in the absence of interactions between variants (Guan and Stephens 2011; Ehret et al. 2012).

For all analyses, we obtained 10 million Markov chain Monte Carlo samples from the joint posterior probability distribution of model parameters (recording values every 1000 iterations) and discarded the first one million samples as burn-in. To investigate negative frequency-dependent selection, we determined allele branch lengths (which we use as a proxy of allele age) based on the MHC genealogy. Branch length provides a rough estimate of allele age because branch lengths are also influenced by selection, recombination, and gene conversion. We calculated mean branch lengths of alleles in each protein or supertype as a proxy of the protein or supertype branch length (see Table S3 for allele translation table). For MHC variants (alleles, proteins, or supertypes) we performed the following linear regressions in R (R Development Core Team 2013): (1) *PIP ˜* frequency of variants with a negative *β*; (2) *PIP* ∽ branch length of variants with a negative *β*; (3) *PIP* ∽ frequency of variants with a positive *β*; and (4) *PIP* ∽ branch length of variants with a positive *β*. For data in which the relationship appeared curvilinear we performed a quadratic polynomial regression and used ANOVA to evaluate whether addition of the quadratic term improved the model fit (*P *<* *0.05).

To examine whether associations between MHC variants and infection status varied across elevation or latitude, we performed separate BVSR analyses for all individuals across transects, individuals from each transect (T1, T2, and T3), and individuals from low (0–1500 m), middle (1501–3100 m), and high elevations (3101–4150 m) across transects. We obtained a list of variants above the 99% empirical quantile for *PIP*, which are likely to be associated with infection status (Guan and Stephens 2011; Gompert et al. 2013). We then examined associations between infection status and MHC variants above the 99% empirical *PIP* quantile using a generalized linear model (GLM) with binomial errors and a logit link. If a variant fell above the 99% quantile for *PIP* and had a *P *<* *0.05 we considered it significantly associated with infection status. In this way, we substantially reduced our final dataset using two separate significance thresholds.

## Results

### Parasite infection prevalence

Jones et al. (2013) found a total of 49 infected individuals (26.6% prevalence) of which 46 were infected with a single *Haemoproteus* lineage (subgenus *Parahaemoproteus; Haemoproteus* sp. KC480265) with the remaining three infections caused by a different *Haemoproteus* haplotype (*Haemoproteus* sp. KC480266) and two unique *Plasmodium* haplotypes (*Plasmodium* sp. KC480267 and *Plasmodium* sp. KC480268). No individuals were infected with multiple parasite haplotypes (Jones et al. 2013). Given that the other three haplotypes each appeared only once and may impose different selective pressures on hosts, these rare infections were eliminated from subsequent analyses. Infection prevalence was significantly higher in the low latitude transect, T1 (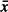 = 42.0%; 95% credible interval: 31.1–53.8%), compared with transects T2 (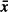 = 16.7%; 95% credible interval: 6.9–23.9%) and T3 (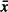 = 18.0%; 95% credible interval: 8.0–24.4%; *P *<* *0.0001; Fig.1). Infection prevalence did not differ significantly between T2 and T3. Prevalence at middle elevations (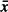 = 64.7%; 95% credible interval: 52.1–88.3%) was significantly higher compared to low (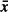 = 13.2%; 95% credible interval: 6.6–24.9%) and high elevations (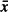 = 15.0%; 95% credible interval: 8.8–24.4%; *P *<* *0.0001; Fig.1) and did not differ between low and high elevation individuals (*P *=* *0.71).

**Figure 2:**
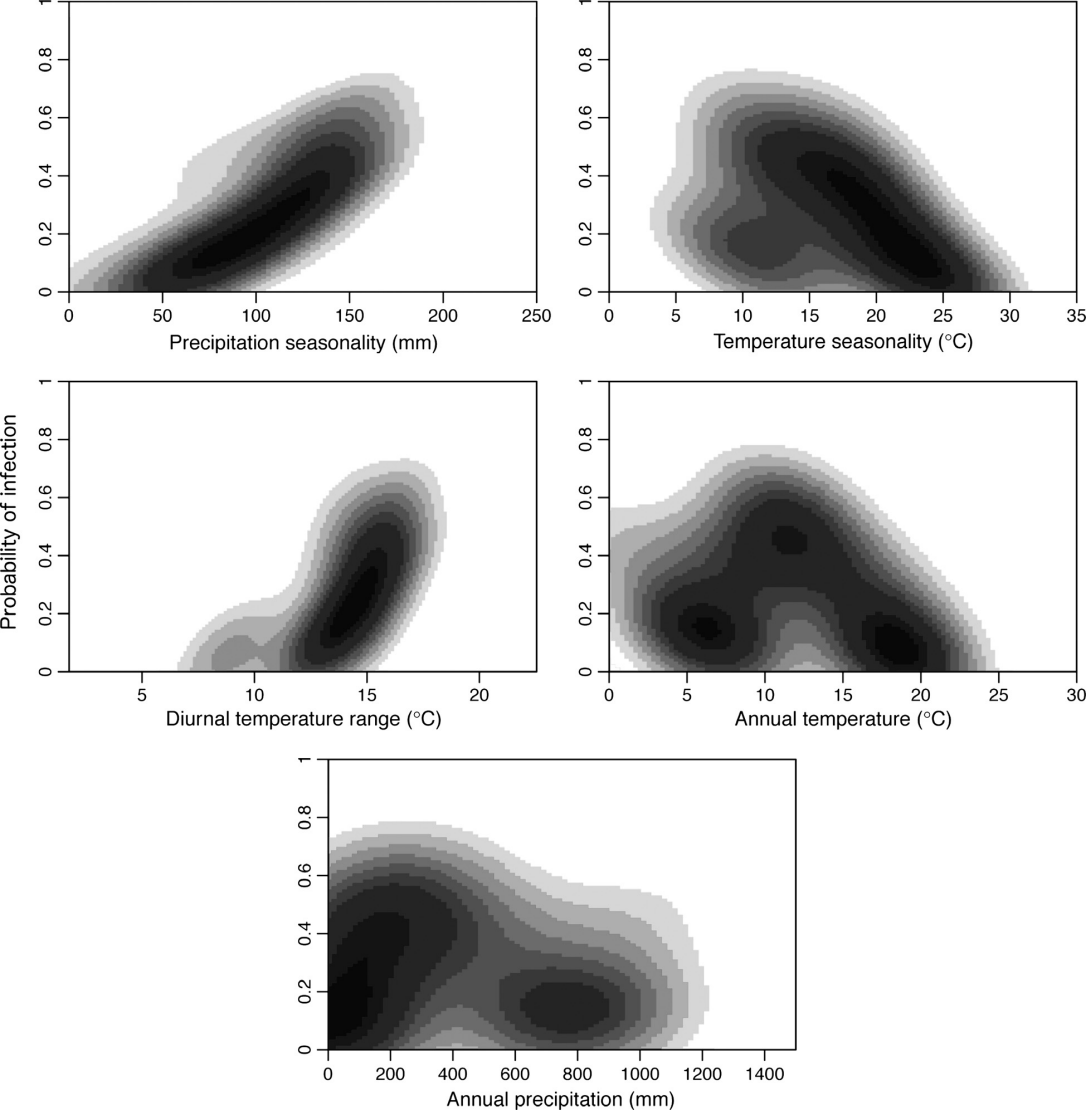
Bioclimatic variables associated with *Haemoproteus* infection in *Zonotrichia capensis* determined by Random Forests. The strength of association between the infection probability and the environmental variable is represented in gray scale with darker colors corresponding to stronger associations. Precipitation seasonality is the coefficient of variation of annual precipitation and temperature seasonality is measured in standard deviations. Diurnal temperature range is the mean of monthly (maximum temperature–minimum temperature) (worldclim.org).

Five environmental variables explained 14.3% of variation (pseudo-*R*^2^) in infection prevalence (mean of squared residuals = 0.0750; Fig.2). The next best model explained only 5.6% of variation in infection prevalence. The abiotic variables included in the optimal random forest model, in order of importance, were precipitation seasonality (bio15; % increase mean squared error (MSE) = 0.012), temperature seasonality (bio4; % decrease MSE = 0.011), diurnal temperature range (bio2; % decrease MSE = 0.008), annual mean temperature (bio1; % decrease MSE = 0.010), and annual precipitation (bio12; % decrease MSE = 0.006). The OOB estimated error rate of the optimal model was 16.7%.

### MHC allelic and supertype diversity

The culled MHC dataset consisted of 98 putatively functional MHC alleles that comprised 56 unique protein sequences (Jones et al. 2014). For putatively functional alleles, Jones et al. (2014) found a model of positive selection (M2) was supported over a nearly neutral model of codon evolution (M1; likelihood ratio = 87.4; *P *<* *0.001). Four codons (positions 18, 53, 61, and 70) showed strong evidence of positive selection (*d*_*N*_*/d*_*S*_ = 5; *P *<* *0.01), one of which (codon 53) corresponded to a peptide-contact site on the *α*-helix of class I MHC, assuming conservation of the peptide-binding region between *Z. capensis* and *G. gallus*. Based on similarities in hydrophobicity, steric bulk, polarity, and electronic effects at positively selected sites, alleles were clustered into 10 MHC supertypes.

Number of MHC alleles, proteins, and supertypes per individual was significantly higher in middle elevations compared to low and high elevation (Jones et al. 2014). MHC allele and supertype diversity per individual was not significantly different between transects (T1–T3) after controlling for elevational zone effects; however, MHC protein diversity per individual was significantly elevated in T1 (Jones et al. 2014). Euclidean genetic distance (distance between population allele vectors) at MHC was not significantly correlated with Euclidean geographic distance (one-tailed *P *=* *0.144; Mantel *r *=* *0.133).

### Associations between infection status and MHC variants

**Figure 3:**
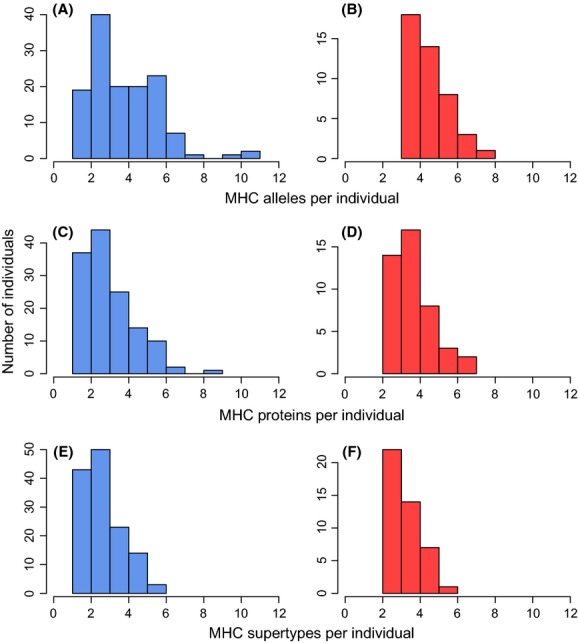
Mean number of MHC alleles (A–B), proteins (C–D), and supertypes (E–F) for uninfected (blue) and infected (red) individuals.

Parasite infection status was not significantly correlated with the number of alleles per individual (*P *=* *0.56), the number of proteins per individual (*P *=* *0.24), the number of supertypes per individual (*P *=* *0.20), or the number of rare alleles per individual (*P *=* *0.43) after controlling for the effect of elevational zone (Fig.3).

The MHC allele dataset explained 23.1% of variance in infection prevalence across all individuals (Table1). The lowest proportion of infection variance explained by MHC was found in the T2 analysis (*PVE* = 0.200), and the highest proportion of variance accounted for by the dataset was in the T1 analysis (*PVE* = 0.508). However, 95% empirical quantiles for the *PVE* and *σ* parameter estimates were large (Table1). Interestingly, MHC proteins and supertypes explained a lower proportion of variance on average compared to alleles (e.g., proteins explained 20.8% and supertypes explained 15.4% of variance for all individuals, Table1). The highest *PVE* for MHC supertypes was in low elevations (*PVE* = 0.223), and the highest *PVE* for proteins was in middle elevations (*PVE* = 0.264).

**Table 1:** Parameter estimates from Bayesian variable selection regression analyses. Parameters estimated include the proportion of variance explained (*PVE*) and the mean phenotypic effect of a variant in a model (*σ*). 95% empirical quantiles are reported in parentheses

Analyses	*PVE*_allele_	*σ*_allele_	*PVE*_protein_	*σ*_protein_	*PVE*_supertype_	*σ*_supertype_
All individuals	0.231(0.050–0.486)	0.426(0.144–1.004)	0.208(0.037–0.475)	0.470(0.145–1.185)	0.154(0.009–0.666)	0.551(0.082–2.590)
T1	0.508(0.056–0.965)	0.754(0.163–2.648)	0.233(0.013–0.639)	0.715(0.100–3.246)	0.210(0.008–0.741)	0.792(0.078–3.582)
T2	0.200(0.007–0.668)	0.800(0.067–4.100)	0.190(0.005–0.726)	0.984(0.670–5.027)	0.176(0.004–0.721)	0.691(0.056–3.154)
T3	0.271(0.013–0.739)	0.665(0.087–2.970)	0.198(0.007–0.681)	0.885(0.081–4.689)	0.196(0.005–0.728)	0.783(0.069–3.546)
Low elevation	0.227(0.008–0.711)	0.877(0.074–4.300)	0.238(0.010–0.721)	0.888(0.090–4.153)	0.223(0.007–0.727)	0.767(0.083–3.325)
Middle elevation	0.229(0.008–0.701)	0.762(0.068–3.918)	0.264(0.013–0.712)	0.695(0.093–3.151)	0.190(0.005–0.732)	0.681(0.058–3.256)
High elevation	0.207(0.008–0.631)	0.782(0.074–4.130)	0.203(0.009–0.636)	0.784(0.089–4.034)	0.203(0.007–0.713)	0.781(0.083–3.620)

**Figure 4:**
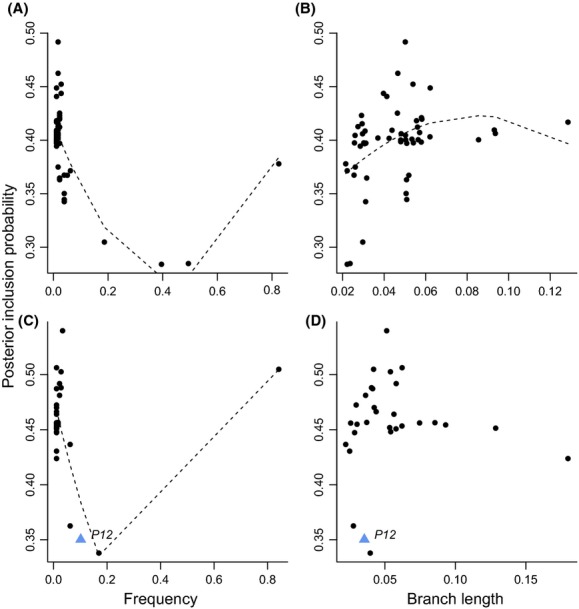
The posterior inclusion probability (*PIP*, degree of association with infection) across all individuals and its association with (A) allele frequency, (B) allele branch length, (C) protein frequency, and (D) mean protein branch length for alleles and proteins with a negative *β*. The dashed lines represent the polynomial regression fit lines. Note that *P12* has a low *PIP* across all individuals despite having a high *PIP* in middle elevation individuals.

Across all individuals, allele and protein sequences associated with absence of infection (*β* < 0) showed a strong negative curvilinear relationship between *PIP* and frequency (alleles: *P *<* *1 × 10^−9^; *R*^2^* *= 0.557; proteins: *P *<* *1 × 10^−4^; *R*^2^* *= 0.503; Fig.4A and 4C), suggesting variants correlated most strongly with resistance are rare. When an outlier protein (Fig.4C) with a relatively high frequency and *PIP* was removed, the relationship was still significant but linear rather than curvilinear (*P *<* *1 × 10^−4^; *R*^2^* *= 0.486). We also found a positive curvilinear relationship between *PIP* and branch length estimate (*P *=* *0.007; *R*^2^* *= 0.175; Fig.4B) for alleles associated with absence of infection (*β* < 0), suggesting the alleles with strong associations are older. No significant relationship was found between *PIP* and branch length for proteins associated with absence of infection (*P *=* *0.196; Fig.4D). For alleles associated with the presence of infection (*β* = 0), we found no significant relationships between *PIP* and allele frequency (*P *=* *0.465) or branch length (*P *=* *0.891). We also found no associations between supertype frequencies or mean supertype branch length and *PIP*.

Seven MHC variants (two alleles, three proteins, and two supertypes) were strongly associated with infection status (above 99% *PIP* quantile and *P *<* *0.05) across different environments (Table2). One MHC variant – protein *P12 –* was significantly associated with infection absence in middle elevations (Table2). Absence of *P12* corresponded to a 33% probability of infection while the presence of *P12* decreased the probability of infection by ∽26% in middle elevation individuals. The remaining variants (two alleles, two proteins, and two supertypes) were significantly correlated with infection presence. The allele ZocaU*6 was significantly associated with infection presence across all pooled individuals yet only for T2 individuals when groups were analyzed separately. MHC protein sequence *P6* also showed a significant positive correlation with *Haemoproteus* across all individuals but only for T1 individuals in separate analyses. The allele ZocaU*3 and protein *P3*, which comprises ZocaU*3, were associated with infection presence in both T3 and high elevation individuals. Supertypes *S4* and *S8* were associated with infection presence in all pooled individuals and T1 individuals, respectively. No MHC variants were correlated with infection presence at low or middle elevations.

**Table 2:** MHC alleles, proteins, and supertypes associated with either absence or presence of *Haemoproteus* infection across different environments. The percent change in infection probability associated with each variant is shown in parentheses

Analyses	Infection absence			Infection presence		
	Alleles	Proteins	Supertypes	Alleles	Proteins	Supertypes
All individuals	–	–	–	ZocaU^*^6 (+22.0%)	*P6* (+17.5%)	*S4* (+15.2%)
T1	–	–	–	–	*P6* (+18.2%)	*S8* (+19.7%)
T2	–	–	–	ZocaU^*^6 (+28.9%)	*–*	*–*
T3	–	–	–	ZocaU^*^3 (+33.5%)	*P3* (+31.5%)	*–*
Low elevation	–	–	–	–	–	–
Middle elevation	–	*P12* (−26.2%)	–	–	–	–
High elevation	–	*–*	*–*	ZocaU^*^3 (+30.7%)	*P3* (+29.3%)	*–*

## Discussion

We investigated patterns of MHC variation in *Z. capensis* and its associations with *Haemoproteus* infections across elevational and latitudinal gradients. Previous research revealed a single *Haemoproteus* (subgenus *Parahaemoproteus*) parasite lineage caused ∽94% of haemosporidian infections and that prevalence was significantly higher at middle elevations across transects (64.7%) and at the low latitude transect (42.0%; Fig.1; Jones et al. 2013). The distributions and abundances of certain avian haemosporidian strains are highly dependent on environmental conditions (Valkiūnas 2005; LaPointe et al. 2012). The development of *Plasmodium relictum* in its vector host, *Culex quinquefasciatus*, cannot occur below ∽13°C, which limits the parasite's upper altitudinal distribution to ∽1800 m above sea level in the Hawaiian Islands (LaPointe et al. 2010). We tested whether similar environmental constraints on *Haemoproteus* may have contributed to the prevalence patterns observed by Jones et al. (2013).

A low probability of *Haemoproteus* infection is most strongly correlated with low precipitation seasonality (Fig.2). Ceratopogonid midges in the genus *Culicoides*, which are believed to be the sole vectors of *Parahaemoproteus* parasites (Valkiūnas 2005), often show substantial seasonal variation in abundance correlated with fluctuations in precipitation (Veggiani Aybar et al. 2010, 2012). The emergence of *Culicoides* often coincides with dry periods preceded by periods of substantial rainfall (Carrasco et al. 2014), which may explain why high precipitation seasonality corresponds to increased infection probability. Temperature seasonality and diurnal temperature range are also strong predictors of *Haemoproteus* prevalence and are highly correlated with prevalence of *Plasmodium* in an African passerine (Seghal et al. 2011). Breeding of most *Culicoides* species occurs in habitats with high water availability (Conte et al. 2007). On the western slope of the Andes water availability is predicted to peak at middle elevations as a result of increased precipitation relative to low elevations and persistence of water runoff from high elevations (McCain 2007). A high probability of *Haemoproteus* infection corresponds with intermediate temperatures and precipitation (Fig.2), which may be driven by preference of *Culicoides* vectors for intermediate elevation habitats with high abundance of water. Bat and plant diversities on the western slope of the Andes also peak at middle elevations (Kalin Arroyo et al. 1988; McCain 2007) and are strongly positively correlated with water abundance and precipitation levels. Thus, the spatial patterns of *Haemoproteus* prevalence we observed may be highly influenced by parasite and vector abundances, which seems to be mediated by the local temperature and precipitation regimes.

We do not know fitness, infection stage (acute or chronic), or parasite load in *Z. capensis*, which limits our ability to infer that *Haemoproteus*-induced selection varies spatially. High MHC diversity observed in individuals inhabiting high *Haemoproteus* prevalence environments (Jones et al. 2014) may be driven by demographic processes (Winternitz et al. 2014), stronger MHC-based sexual selection (Winternitz et al. 2013), or intensified parasite-mediated selection in these environments (Spurgin and Richardson 2010). Here we examine predictions of parasite-mediated selection in the absence of a statistical null model as we lack a sufficient number of neutral markers or phased MHC alleles to infer deviations from neutrality.

### Inferences of parasite-mediated selection

After controlling for the covariance between elevation and MHC diversity, infected *Z. capensis* individuals did not significantly differ from uninfected individuals in the number of MHC alleles, proteins, or supertypes they possessed (Fig.3). This finding is inconsistent with a role of heterozygote advantage driven by *Haemoproteus*; however, more complete surveys of parasite prevalence in *Z. capensis* are needed to completely rule out heterozygote advantage imposed by other pathogens.

Negative frequency-dependent selection predicts that alleles associated with pathogen resistance will be rare in the population. We examined this prediction and found conflicting support. The variant *P12 –* the only variant associated with lack of infection in any environment – is relatively young and found at a moderate frequency across all individuals (10.2%; Fig.4C and D). *P12* also had one of the lowest *PIP*s across all individuals even though it had the highest *PIP* in middle elevation individuals (Fig.4C and D). MHC nucleotide and protein sequences, although not supertypes, associated with decreased *Haemoproteus* infection were typically rare (Fig.4A and C). MHC nucleotide sequences associated with decreased *Haemoproteus* infection also tended to have long branches (Fig.4B), suggesting they are relatively old. Such a pattern may arise if MHC alleles are “recycled” during host–parasite coevolutionary cycles (Lively 2001; Eizaguirre et al. 2012b). Negative frequency-dependent selection is expected to result in high MHC diversity as selection acts against both fixation and loss of alleles (Slade and McCallum 1992; Eizaguirre and Lenz 2010). Selection on standing genetic variation may allow host populations to quickly evolve resistance to parasites and maintain pace with rapidly evolving parasites (Dionne et al. 2007; Barrett and Schluter 2008; Eizaguirre et al. 2012b). MHC supertypes, which we expect to be a more accurate representation of disease resistance than alleles or proteins, do not show evidence of negative frequency-dependent selection. The incongruence between datasets suggests either spurious correlations in the allele and protein datasets, differences in parasite-mediated selection acting on different components of MHC variation (e.g., negative frequency-dependent selection on nonpositively selected sites), or incorrect or incomplete characterization of functionally important sites on the MHC. We detected only four positively selected sites on MHC class I exon 3 in *Z. capensis* while Alcaide et al. (2013) reports 11 sites with high probabilities of positive selection across 16 passerine species examined. While the PBR generally comprises the positively selected sites in humans and mice (Hughes and Nei 1988; Hughes and Yeager 1998), historical selection and stochastic forces can shape *d*_*N*_/*d*_*S*_ ratios and inferences of positively selected sites (Mugal et al. 2013). Thus, experimental validation of the PBR is preferable because evidence of selection at the site level may not translate into important functional differences between alleles. For example, in Pacific slope *Z. capensis*, patterns of spatial variation in hemoglobin (Hb) polymorphisms suggests local adaptation along elevational gradients, but functional experiments revealed that elevationally segregating Hb variants do not differ in their oxygenation properties (Cheviron et al. 2014). Sepil et al. (2013) also advocate an experimental approach to identify the PBR after finding that including divergent alleles can dramatically alter the outcome of supertype clustering based on positively selected sites. However, because of the inherent problems identifying the PBR bioinformatically and the difficulty of experimentally validating peptide-binding residues, identifying functionally important MHC variation may remain a major obstacle for nonmodel species without an annotated genome.

In addition to negative frequency-dependent selection, we tested a prediction of fluctuating spatial selection that variants associated with infection will be population specific (Spurgin and Richardson 2010). Overall, we found seven MHC variants with significant environment-specific associations with *Haemoproteus* infection status, although the mechanisms giving rise to this pattern are unclear. Stochasticity in variant sampling across environments may have produced false-positive associations (e.g., a variant is randomly sampled only in infected individuals). Incongruence between variant types (alleles, proteins, and supertypes) associated with infection in the same environment suggests this may contribute. For instance, variants *P6* and *S8* are associated with infection presence for T1 individuals; however, *P6* falls within a different supertype cluster (*S4*). Variant *P12* falls within the same supertype cluster (*S10*) as ZocaU*3 and *P3* although it has an opposite direction of association, indicating either that these associations are spurious or that our supertype classification lacks important functional variation. Alternatively, these discrepancies may reflect different components of MHC variation contributing different forms of resistance to parasites across the landscape.

The variant *P12* was associated with a ∽26% decreased infection probability in middle elevation individuals. *Haemoproteus* prevalence is elevated in middle elevations, which may confer a concomitant increase in the strength of parasite-induced selection. The higher frequency of *P12* in middle elevations (17.5%) relative to low (11.1%) and high elevations (5.6%) may suggest that this variant is undergoing a selective sweep in middle elevations and is less beneficial at other elevations. We identified six variants associated with infection presence in environments of low *Haemoproteus* prevalence. Many hypotheses have been put forth to explain the persistence of genetic variation associated with increased parasite infection. Fluctuating parasite communities and abiotic conditions can decrease the long-term efficacy of selection on host populations, which may maintain functional immunogenetic variation in hosts and even deleterious susceptibility variants (Lazzaro and Little 2009). Variants conferring susceptibility to harboring a particular parasite may also arise and remain in populations due to counter-selection imposed by other diseases, favoring an allele conferring resistance to one at the expense of susceptibility to another (Bonneaud et al. 2006; Loiseau et al. 2008). This may explain the abundance of variants correlated with infection presence in low prevalence environments where *Haemoproteus* is expected exert the weakest selection. In the absence of fluctuating selection, susceptibility variants may be maintained in populations by drift if they are only mildly deleterious, which may be the case with *Haemoproteus* parasites, whose fitness effects during chronic infection vary from no observable effects (Valkiūnas 2005; Bensch et al. 2007) to sex-specific reduced survival in the most extreme circumstances (Martínez-de la Puente et al. 2010). These variants may also be interpreted as conferring quantitative resistance, that is, they that do not eliminate infection but allow a host to cope with infection (Westerdahl et al. 2012; Sepil et al. 2013). Hence, noncarriers that become infected may be more likely to die, resulting in a positive association between allele presence and infection presence. Additional data on fitness consequences of avian haemosporidian infection, spatial patterns of prevalence for other diseases, and associations between MHC and other diseases in *Z. capensis* are needed to test these hypotheses.

## Conclusions

Our results were consistent with predictions of negative frequency-dependent selection and fluctuating selection on *Z. capensis* MHC, although alternative mechanisms, including demographic processes, may also influence these patterns. Our study highlights some of the difficulties in testing and inferring mechanisms of parasite-mediated selection acting on natural host populations and their parasites. In particular, we demonstrate the challenges of identifying the functionally important sites in MHC and interpreting associations between parasites and MHC across different levels of variation. MHC nucleotide and protein sequences showed consistent trends with negative frequency-dependent selection; however, MHC supertypes showed no trend. The environment-specific association patterns we found between MHC variants and infection, while expected under fluctuating selection, are difficult to attribute to selection in the absence of detailed fitness or functional data. Nonetheless, our study may provide insight into the dynamics of parasite-mediated selection and host–parasite coevolution in a natural vertebrate host–parasite system. This contributes to a growing body of work that suggests environmental variability may be important in maintaining MHC polymorphism across broad scales and promoting local adaptation in MHC to parasite communities at relatively fine spatial scales.

## Data Accessibility

*Plasmodium* and *Haemoproteus cyt* b sequences: GenBank Accessions KC480265–KC480268.

MHC sequences: GenBank Accessions KF433977–KF434074.

## Supporting Information

**Data S1**. MHC variant data per individual.

**Table S1.***Zonotrichia capensis* specimen data. **Table S2.** Sampling locality information. **Table S3.** Translation table between MHC alleles, proteins, and supertypes.
