# Supplementary material for: Spatially variable coevolution between a haemosporidian parasite and the MHC of a widely distributed passerine
Source: Ecol Evol. 2015 Feb 6;5(5):1045–60. doi: 10.1002/ece3.1391 (PMC4364819; doi:10.1002/ece3.1391)
Supplement: Supplementary file 1 [file ece30005-1045-sd1.pdf]

MBE Advance Access published August 26, 2014

Integrating Evolutionary and Functional Tests of Adaptive Hypotheses: A Case Study of Altitudinal Differentiation in Hemoglobin Function in an Andean Sparrow, *Zonotrichia capensis*

Zachary A. Cheviron,*[,1,2] Chandrasekhar Natarajan,[2] Joana Projecto-Garcia,[2] Douglas K. Eddy,[1] Jennifer Jones,[1] Matthew D. Carling,[3] Christopher C. Witt,[4,5] Hideaki Moriyama,[2] Roy E. Weber,[6] Angela Fago,[6] and Jay F. Storz*[,2]

[1]Department of Animal Biology, School of Integrative Biology, University of Illinois, Urbana-Champaign

[2]School of Biological Sciences, University of Nebraska, Lincoln

[3]Department of Zoology and Physiology, University of Wyoming

[4]Department of Biology, University of New Mexico

[5]Museum of Southwestern Biology, University of New Mexico

[6]Zoophysiology, Department of Bioscience, Aarhus University, Aarhus, Denmark

***Corresponding author:** E-mail: cheviron@illinois.edu; jstorz2@unl.edu.

Associate editor: Joshua Akey

Abstract

In air-breathing vertebrates, the physiologically optimal blood-O_2 affinity is jointly determined by the prevailing partial pressure of atmospheric O_2, the efficacy of pulmonary O_2 transfer, and internal metabolic demands. Consequently, genetic variation in the oxygenation properties of hemoglobin (Hb) may be subject to spatially varying selection in species with broad elevational distributions. Here we report the results of a combined functional and evolutionary analysis of Hb polymorphism in the rufous-collared sparrow (*Zonotrichia capensis*), a species that is continuously distributed across a steep elevational gradient on the Pacific slope of the Peruvian Andes. We integrated a population genomic analysis that included all postnatally expressed Hb genes with functional studies of naturally occurring Hb variants, as well as recombinant Hb (rHb) mutants that were engineered through site-directed mutagenesis. We identified three clinally varying amino acid polymorphisms: Two in the α^A-globin gene, which encodes the α-chain subunits of the major HbA isoform, and one in the α^D-globin gene, which encodes the α-chain subunits of the minor HbD isoform. We then constructed and experimentally tested single- and double-mutant rHbs representing each of the alternative α^A-globin genotypes that predominate at different elevations. Although the locus-specific patterns of altitudinal differentiation suggested a history of spatially varying selection acting on Hb polymorphism, the experimental tests demonstrated that the observed amino acid mutations have no discernible effect on respiratory properties of the HbA or HbD isoforms. These results highlight the importance of experimentally validating the hypothesized effects of genetic changes in protein function to avoid the pitfalls of adaptive storytelling.

Key words: **adaptation, functional synthesis, high-altitude adaptation, hypoxia, protein evolution.**

Article

Downloaded from http://mbe.oxfordjournals.org/ at University of Nebraska-Lincoln Libraries on August 29, 2014

Introduction

High-altitude hypoxia can impose severe constraints on the capacity for aerobic exercise and thermogenesis in endothermic vertebrates. To some degree, these constraints on organismal performance can be mitigated through physiological adjustments that enhance the flux capacity of the O_2-transport pathway. In birds and other vertebrates, the fine-tuned modulation of blood-O_2 affinity represents an especially well-documented mechanism for enhancing circulatory O_2 conductance under hypoxia (Petschow et al. 1977; Monge and León-Velarde 1991; Nikinmaa 2001; Samaja et al. 2003; Scott and Milsom 2006; Storz, Scott, et al. 2010; Scott 2011; Mairbaurl and Weber 2012). In many cases, adaptive changes in blood-O_2 affinity stem directly from genetically based changes in hemoglobin (Hb) function. Under severe hypoxia, an increase in Hb-O_2 affinity can help preserve an adequate level of tissue oxygenation by enhancing pulmonary O_2 uptake while simultaneously maintaining the pressure gradient that drives O_2 diffusion from capillary blood to the tissue mitochondria (Storz, Scott, et al. 2010). However, an elevated Hb-O_2 affinity is not unconditionally beneficial with increasing elevation. Under moderate levels of arterial O_2 desaturation (as might be experienced at intermediate altitudes), theory predicts that tissue oxygenation is actually increased by a reduced Hb-O_2 affinity because the benefits of increasing O_2 unloading in the systemic circulation more than offset the diminishing marginal returns of safeguarding arterial O_2 saturation (Turek et al. 1973; Turek and Kreuzer 1976; Turek, Kreuzer, Ringnalda 1978; Turek, Kreuzer,

© The Author 2014. Published by Oxford University Press on behalf of the Society for Molecular Biology and Evolution. All rights reserved. For permissions, please e-mail: journals.permissions@oup.com

Turek-Maischeider, et al. 1978; Bencowitz et al. 1982; Willford et al. 1982; Mairbaurl and Weber 2012).

As the optimal blood-O_2 affinity is expected to vary as a function of altitude, genetic variation in Hb function may be subject to spatially varying selection in species that inhabit a broad range of elevations. Thus, studies of population-level variation in species that are distributed across steep elevational gradients provide some of the best opportunities for investigating microevolutionary mechanisms of physiological adaptation to hypoxia. The rufous-collared sparrow (*Zonotrichia capensis*) is especially well-suited to intraspecific studies of hypoxia adaptation because it has one of the broadest elevational distributions of any passerine bird. In the Peruvian Andes, *Z. capensis* is continuously distributed from sea-level to elevations of over 4,500 m above sea-level (a.s.l.) (Schulenberg et al. 2007), and populations at the extremes of this altitudinal distribution are connected by high levels of gene flow (Cheviron and Brumfield 2009). Nonetheless, populations that are native to different elevations exhibit a number of physiological differences that are likely to be adaptive. For example, sparrows that are native to high altitude (> 4,500 m a.s.l.) have higher summit metabolic rates and lower critical temperatures (i.e., the temperature at which metabolic resources must be used to maintain constant body temperature) compared with their lowland counterparts (Castro 1983; Castro et al. 1985; Castro and Wunder 1990).

Here, we report the results of a combined functional and evolutionary analysis of Hb polymorphism in *Z. capensis* that were sampled from replicate elevational transects along the Pacific slope of the Peruvian Andes. We integrate a population genetic analysis of DNA sequence variation in adult Hb genes with functional studies of naturally occurring Hb variants, as well as recombinant Hb (rHb) mutants that were engineered through site-directed mutagenesis. We place these results within the broader context of genomic differentiation between high- and low-elevation populations through a survey of 483 randomly sampled restriction-site associated DNA (RAD) polymorphisms that map to putative *Zonotrichia* protein-coding genes. The population genetic analyses revealed three clinally varying amino acid polymorphisms: Two in the α^A-globin gene, which encodes the α-chain subunits of the major adult Hb isoform (HbA), and one in the α^D-globin gene, which encodes the α-chain subunits of the minor isoform (HbD). The locus-specific patterns of clinal variation and altitudinal differentiation suggested a history of spatially varying selection between populations of *Z. capensis* that are native to different elevations. However, the experimental tests demonstrated that the observed amino acid mutations have no discernible effect on respiratory properties of the HbA or HbD isoforms. These results demonstrate the importance of experimentally validating inferences about molecular adaptation that are derived from population genetic data.

Results

Our analysis of Hb variation in *Z. capensis* was based on a total of 197 museum-vouchered specimens collected from a broad range of elevations in the Peruvian Andes (supplementary table S1, Supplementary Material online). For the population genetic analysis of Hb polymorphisms, we collected a total of 168 individual specimens along a series of replicate transects on the Pacific slope of the Andes (fig. 1). We surveyed three separate elevational transects (T1–T3) that spanned nearly 4,000 m in vertical relief over relatively short geographic distances (mean elevational gain = 3,840 m [range 3,700–4,105 m], mean linear distance = 166.8 km [range 123–213 km]), and four latitudinal control transects (C1–C4) that spanned a much greater geographic distance but over a relatively uniform elevation (mean elevational gain = 165 m [range 10–435 m], mean linear distance = 495.9 km [range 286–762 km]) (fig. 1). This sampling design allowed us to separate the effects of altitude and geographic distance on population genetic structure. For the functional analyses of native Hb variants, we also obtained blood samples from 29 specimens with known globin genotypes that were collected from low- or high-altitude localities (<800 and > 3,500 m a.s.l., respectively; supplementary tables S1 and S2, Supplementary Material online).

Hb Isoform Composition

We used a combination of isoelectric focusing (IEF) and tandem mass spectrometry (MS/MS) to characterize Hb isoform composition in the circulating erythrocytes of adult *Z. capensis*. These analyses revealed that adult sparrows express two structurally distinct Hb isoforms, HbA (pI = 8.7–8.8) and HbD (pI = 7.3–7.5; fig. 2), consistent with data from the majority of other avian taxa examined to date (Grispo et al. 2012). The HbA isoform ($\alpha_2^A \beta_2^A$) incorporates products of the α^A-globin gene, whereas the minor HbD isoform ($\alpha_2^D \beta_2^A$) incorporates products of the α^D-globin gene; both isoforms share the same β-type subunit (Hoffmann and Storz 2007; Grispo et al. 2012). In the hemolysates of wild-caught sparrows, HbA was present at a consistently higher concentration than the alternative HbD isoform, but there was substantial interindividual variation. The relative abundance of HbD tended to be slightly higher in the red cells of highland sparrows but the altitudinal difference was not statistically significant: The median proportion of HbD was 0.32 in the highland birds (range = 0.23–0.67, n = 13) versus 0.26 in the lowland birds (range = 0.16–0.52, n = 17, Mann–Whitney U = 68.5, two-tailed P = 0.081). MS/MS analysis confirmed that the subunit components of the two adult Hb isoforms represent products of the α^A-, α^D-, and β^A-globin genes. We therefore focused our population genetic analyses on these three genes.

Altitudinal Patterns of Sequence Polymorphism

The three adult-expressed globin genes exhibited a broad range of nucleotide variation, with silent-site diversities spanning an order of magnitude, from π = 0.0115 for β^A-globin to π = 0.0054 and 0.0095 for α^A- and α^D-globin, respectively (table 1). Measures of the site-frequency spectrum (Tajima's D) and intralocus linkage disequilibrium (LD) (Z_{nS}) did not exhibit significant departures from neutral-equilibrium expectations for any of the three genes.

Downloaded from http://mbe.oxfordjournals.org/ at University of Nebraska-Lincoln Libraries on August 29, 2014

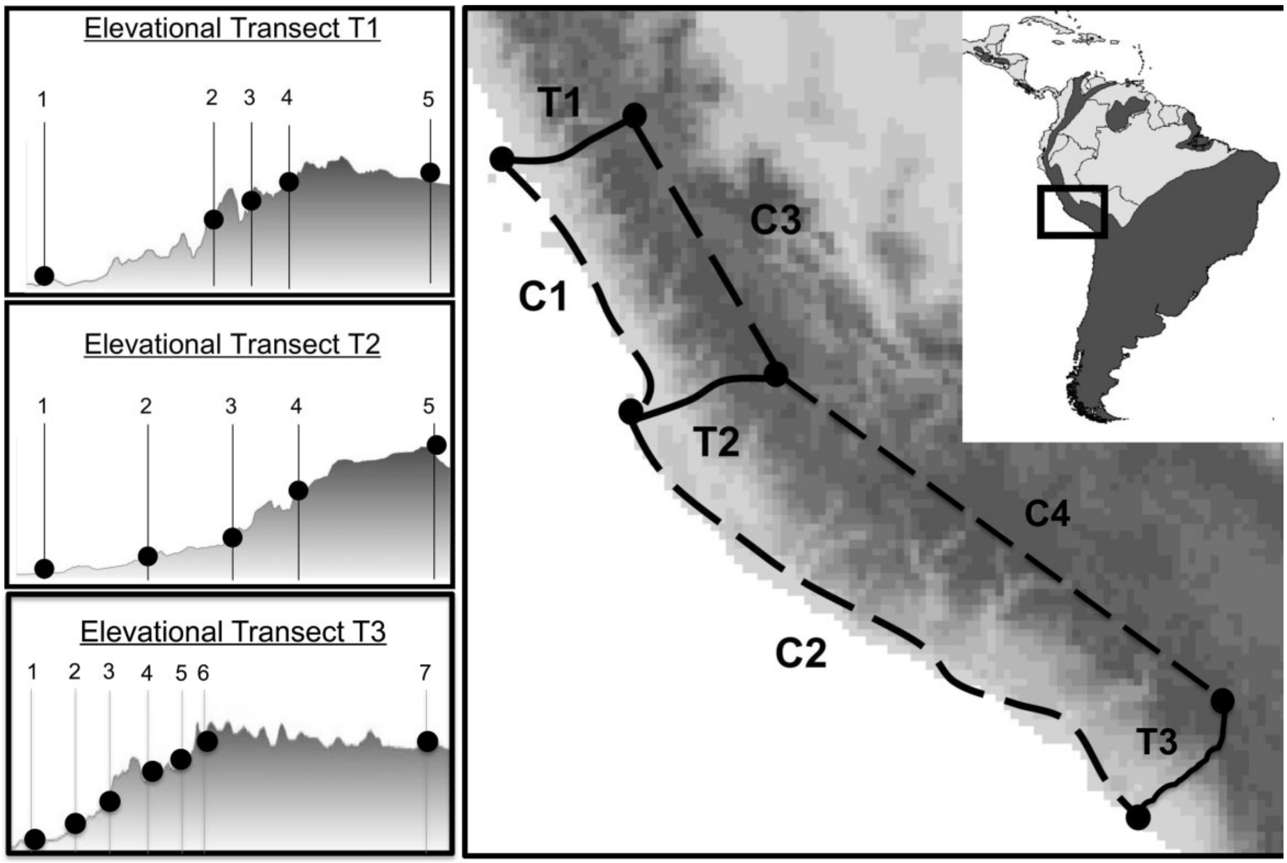

Fig. 1. Sampling design. Elevational transects and control transects are illustrated as solid lines and dashed lines, respectively. Locations of sampling sites along each elevational transect are depicted as black dots. Geographic coordinates and elevations of the sampling localities are listed in supplementary table S1, Supplementary Material online. The geographic distribution of *Zonotrichia capensis* is depicted by grey shading in the inset map.

The sequence data revealed three amino acid polymorphisms that exhibited dramatic altitudinal shifts in allele frequency: Two in the α^A-globin gene (49Gly/Ala and 77Ala/Ser) and one in the α^D-globin gene (50Pro/Ser). Site α^A49 is the seventh residue position of the CD interhelical loop (CD7), site α^A77 is the sixth position of the EF interhelical loop (EF6), and site α^D50 is the eighth residue position of the CD interhelical loop (CD8); each of the three sites is an exterior, solvent-exposed residue position. At all three sites, the derived (nonancestral) variant was present at high frequency in the high-altitude population samples. In the case of the α^A-globin gene, the two-site haplotype 49Gly–77Ala predominated at low altitude and was replaced by the alternative two-site haplotype 49Ala–77Ser above 3,000 m a.s.l. Although allele frequencies varied across each of the surveyed transects, levels of nucleotide differentiation (F_{st}) for the α^A-globin gene were over four times higher in comparisons between population samples from the endpoints of the elevational transects than in comparisons between endpoints of the control transects (α^A $F_{st\ elevational} = 0.642$, α^A $F_{st\ control} = 0.151$). A similar pattern of nucleotide differentiation was observed for the α^D-globin gene. The α^D-50Pro allele predominated at low elevations and was replaced by the alternative α^D-50Ser allele above 2,500 m a.s.l. Again, levels of nucleotide differentiation across elevational transects greatly exceeded those across the control transects, although this difference was not as great as that observed for the α^A-globin gene (α^D $F_{st\ elevational} = 0.453$, α^D $F_{st\ control} = 0.121$). Consistent with observed levels of nucleotide differentiation across the transects, coalescent-based estimates of effective gene flow (migration rates—*m*) for α^A-globin were 65-fold lower across the elevational transects than the control transects (α^A $m_{control}$:$m_{elevational} = 65.8$; table 2). For the α^D-globin gene, estimated levels of gene flow were also lower across the elevational transects than the control transects, but this reduction was not nearly as great as that observed for the α^A-globin gene (α^D $m_{control}$:$m_{elevational} = 1.79$).

For each clinally varying amino acid polymorphism, we estimated cline shape parameters on each replicate transect to obtain a more fine-grained view of allele frequency variation across the altitudinal gradient. Estimates of cline centers for the two-site α^A-globin haplotype were remarkably similar across the three replicated transects, ranging from roughly 3,400 to 4,000 m a.s.l. (table 3 and fig. 3), and cline widths were relatively narrow, ranging from 59.2 m (transect T3) to 710.8 m (transect T2) (table 3). Altitudinal clines were similar for the amino acid polymorphism in the α^D-globin gene, but were generally wider and were centered at lower elevations. For transects T2 and T3, clines for the α^D-globin polymorphism were centered at 2,930 m and 485 m a.s.l., and estimated cline widths were 1,446 m and 911 m, respectively. Cline shape parameters were not estimated for

Downloaded from http://mbe.oxfordjournals.org/ at University of Nebraska-Lincoln Libraries on August 29, 2014

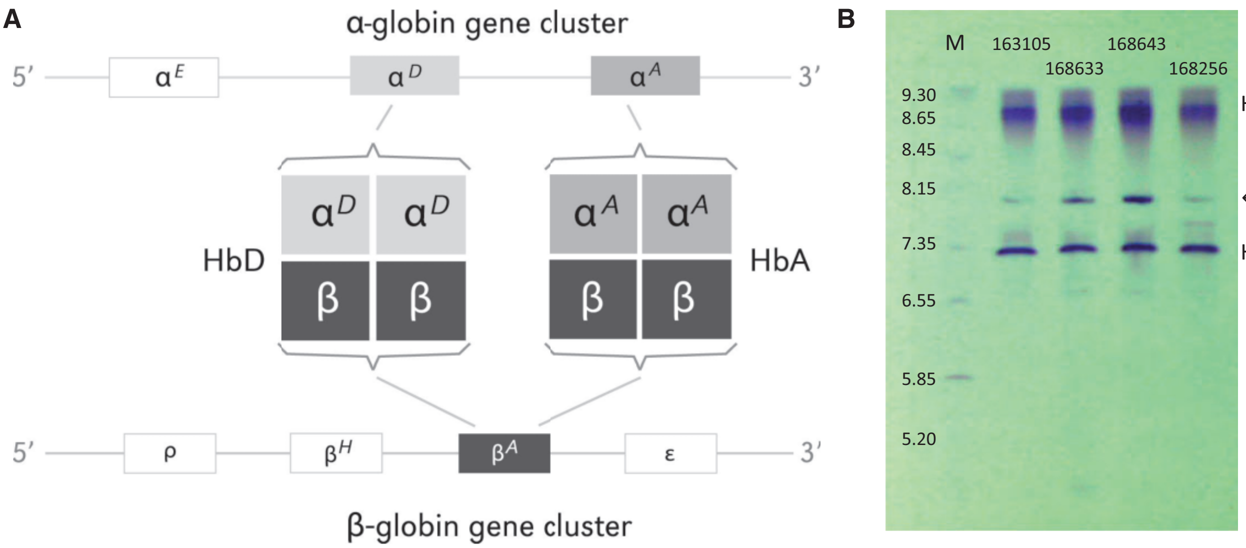

Fig. 2. Postnatally expressed Hb isoforms in the definitive erythrocytes of rufous-collared sparrows, *Zonotrichia capensis*. (*A*) The major isoform, HbA ($\alpha_2^A\ \beta_2$), has α-type subunits encoded by the α^A-globin gene, and the minor isoform, HbD ($\alpha_2^D\ \beta_2$), has α-type subunits encoded by the α^D-globin gene. Both isoforms share identical β-type subunits encoded by the β^A-globin gene. Results of the mass-spectrometry analyses revealed that the remaining members of the α- and β-globin gene families (α^E-, ρ-, β^H-, and ε-globin) are not expressed at detectable levels during adulthood. Within each gene cluster, the intergenic spacing is not drawn to scale. (*B*) IEF gel (pH 3–9) showing the separation of native HbA and HbD isoforms from red cell lysates of four representative specimens of *Z. capensis*. Bands corresponding to native HbA and HbD are indicated (in each case, shadow bands represent native proteins with one or more heme groups in different oxidation states); intermediate bands (indicated by arrow) represent gel-loading points. The leftmost lane (M) shows an IEF standard with corresponding isoelectric points for each band.

Table 1. DNA Polymorphism and Intragenic LD in the Adult-Expressed Globin Genes of *Zonotrichia capensis*.

Gene	Length (bp)	N	S	h	H_d	$\pi_{(silent)}$	$\theta_{W(silent)}$	Tajima's D	4Nc	Z_{nS}
α^D-globin	778	188	44	71	0.951	0.0095	0.0154	−1.1256	0.0230	0.0362
α^A-globin	665	216	20	38	0.884	0.0054	0.0077	−0.7539	0.0693	0.0251
β^A-globin	1,310	60	83	45	0.981	0.0115	0.0169	−1.1147	0.0165	0.0879

Note.—*N*, number of sampled alleles; *S*, number of segregating sites; *h*, number of haplotypes; H_d, haplotype diversity. For each of the three genes, summary measures of the site-frequency spectrum (Tajima's *D*) and intragenic LD (Z_{nS}) were consistent with neutral-equilibrium expectations.

the α^D-globin gene along transect T1 due to the lack of clinal variation.

Analyzing the α^A-globin mutations separately revealed spatial concordance between the clines for the α^A- and α^D-globin genes (table 3, fig. 3). The center for the α^A-77Ala/Ser cline was statistically concordant with the center of the α^D cline on transects T2 and T3, whereas the α^A-49Gly/Ala cline was centered roughly 500–3,300 m upslope (table 3, fig. 3). Thus, the single-mutant α^A-globin haplotype 49Gly–77Ser predominates at intermediate altitudes. Similar to the concordance in cline centers for the two-site α^A-globin haplotype, the frequency of the 49Gly–77Ser haplotype peaked at similar altitudes across all three transects: Transect T1, 3,870 m; transect T2, 3,150 m; and transect T3, 2,900 m. The altitudinal pattern of clinal variation at the α^A- and α^D-globin genes stands in contrast to the lack of altitudinal differentiation at the β^A-globin gene. The β^A-globin gene harbored a single intermediate-frequency replacement polymorphism (β^A-12Ser/Thr), but in contrast to the α^A and α^D polymorphisms, there was no significant difference in allele frequency between high- and low-altitude samples.

Population Genomic Analysis

To examine patterns of altitudinal differentiation at the globin genes in a genome-wide context, we surveyed 483 RAD polymorphisms in 15 high-elevation and 15 low-elevation birds that map to putative *Zonotrichia* protein-coding genes (see Materials and Methods). The overall levels of genomic differentiation between high- and low-elevation populations were low (median site-specific $F_{st} = 0.052$, upper bound of the 95% quantile = 0.242) (fig. 4). Site-specific F_{st} values for all three α-globin amino acid polymorphisms in the same panel of individuals exceeded the 95% quantile of the empirical distribution (fig. 4), a pattern that suggests a possible history of spatially varying selection. Finally, levels of intragenic LD decay to background levels within less than 1 kb in both α-like globin genes (fig. 5), suggesting that the altitudinal patterns of differentiation are not attributable to spatially varying selection at an unsurveyed linked gene.

Oxygenation Properties of HbA and HbD Isoforms

We purified HbA and HbD isoforms from red cell lysates of highland and lowland birds that were alternative double

Downloaded from http://mbe.oxfordjournals.org/ at University of Nebraska-Lincoln Libraries on August 29, 2014

Table 2. Estimates of Symmetrical Migration Rates (m) Using IMa.

Locus	Transect							$m_{control}$:$m_{elevational}$
	T1	T2	T3	C1	C2	C3	C4	
α^A	6.98 (0.725–34.975)	0.63 (0–11.625)	0.125 (0–3.875)	3.375 (0–43.6)	201.1 (45.1–249.9)	246.6 (41.3–249.9)	231.1 (42.1–249.9)	65.8
α^D	8.25 (0.1–95.1)	12.9 (2.1–87.8)	1.25 (0–50.8)	10.05 (1.6–84.5)	16.9 (5.6–747.4)	9.4 (0.6–99.2)	17.3 (2.4–99.9)	1.79

NOTE.—90% highest posterior density intervals are given in parentheses.

Table 3. Maximum-Likelihood Estimates of Altitudinal Cline Centers (c) and Cline Widths (w) for Amino Acid Polymorphisms in the α^A- and α^D-Globin Genes.

Transect	α^D-50Ser		α^A-49Ala		α^A-77Ser		α^A Two-Site Haplotype	
	c	w	c	w	c	w	c	w
T1	—	—	3,894 (3,856–4,128)	250 (0–829)	—	—	3,992 (3,843–4,133)	272 (1–863)
T2	2,930 (1,409–3,476)	1,446 (0–5,455)	3,473 (3,235–3,874)	698 (55–2,203)	3,008 (767–3,114)	243 (0–2,556)	3,411 (3,168–3,895)	711 (57–2,200)
T3	485 (63–1,500)	910 (4–5,270)	3,846 (3,797–4,082)	73 (0–312)	2,285 (478–3,118)	3,172 (2–6,560)	3,860 (3,805–4,091)	59 (0–256)

NOTE.—The two log-likelihood support values are given in parentheses. Cline centers are reported in meters a.s.l. and widths are reported in meters. Allele frequencies for α^D-50Ser and α^A-77Ser did not vary clinally along transect T1 (fig. 3), preventing the estimation of cline shape parameters.

homozygotes at sites α^A49 and α^A77 ($n = 3$ highland birds [49Ala–77Ser] and $n = 6$ lowland birds [49Gly–77Ala]) (supplementary table S2, Supplementary Material online). The nine specimens were all homozygous for the ancestral β^A-12Thr allele. As there was no confounding amino acid variation at other sites in the α^A- or β^A-globin genes, the comparison between highland and lowland HbA variants isolates the net functional effect of mutations at α^A49 and α^A77. Similarly, all of the highland birds were α^D50-Ser homozygotes, so the comparison between highland and lowland HbD variants isolates the functional effect of the mutation at α^D50.

Genetically based changes in Hb function can be brought about by amino acid mutations that alter the intrinsic O_2-binding affinity of the Hb tetramer and/or mutations that alter the sensitivity of Hb to the allosteric effectors that modulate O_2 binding in the red blood cell (Weber and Fago 2004; Mairbaurl and Weber 2012). To gain insight into mechanisms of allosteric regulation, we measured the oxygenation properties of purified HbA and HbD solutions in the presence and absence of two main allosteric effectors that regulate Hb-O_2 affinity: Cl^- ions (added as 0.1 M KCl) and inositol hexaphosphate (IHP [a chemical analog of inositol pentaphosphate found in avian red cells], at 2-fold molar excess over tetrameric Hb). For samples representing each of the highland and lowland genotype classes, analysis of O_2-equilibrium curves revealed that the HbD isoform exhibited a uniformly higher O_2-affinity than the HbA isoform, both in the absence (stripped) and presence of allosteric effectors (figs. 6 and 7). This is demonstrated by the uniformly left-shifted O_2-equilibrium curves for HbD relative to those of HbA under the same treatment conditions (fig. 6). Hence, HbD isoforms exhibited uniformly lower values of P_{50} (the partial pressure of O_2 at which heme is 50% saturated; table 4, fig. 7) compared with HbA isoforms. By calculating a weighted average of $P_{50(KCl+IHP)}$ for HbA and HbD in their naturally occurring relative concentrations, we determined that composite values of Hb-O_2 affinity were essentially identical for the highland and lowland sparrows: 33.13 versus 32.68 torr, respectively.

O_2-affinities of both HbA and HbD were strongly reduced in the presence of IHP (figs. 6 and 7). Although Cl^- and IHP both have the effect of reducing Hb-O_2 affinity, O_2-affinities of HbA and HbD were higher in the simultaneous presence of both effectors than in the presence of IHP alone (fig. 6). This reflects competition for shared cationic binding sites between the weak effector (Cl^-) and the strong effector (IHP), a pattern that has been documented for the Hbs of other bird species (Grispo et al. 2012; Projecto-Garcia et al. 2013). Both isoforms exhibited cooperative O_2-binding; in the presence of allosteric effectors, the range of estimated Hill coefficients (n_{50}'s) was 1.78–2.31 for HbA and 1.96–2.58 for HbD (table 4).

Comparison of High- and Low-Altitude HbA and HbD Variants

In the case of HbA, the highland and lowland variants did not exhibit any detectable difference in intrinsic O_2-affinity (P_{50}'s of "stripped" Hbs were essentially identical; fig. 7A). There were subtle but nonsignificant differences in O_2-affinity in the presence of Cl^- ions, in the presence of IHP (at 2-fold molar excess over tetrameric Hb), and in the simultaneous presence of both effectors (the "KCl + IHP" treatment, which closely approximates the in vivo conditions in avian

Downloaded from http://mbe.oxfordjournals.org/ at University of Nebraka-Lincoln Libraries on August 29, 2014

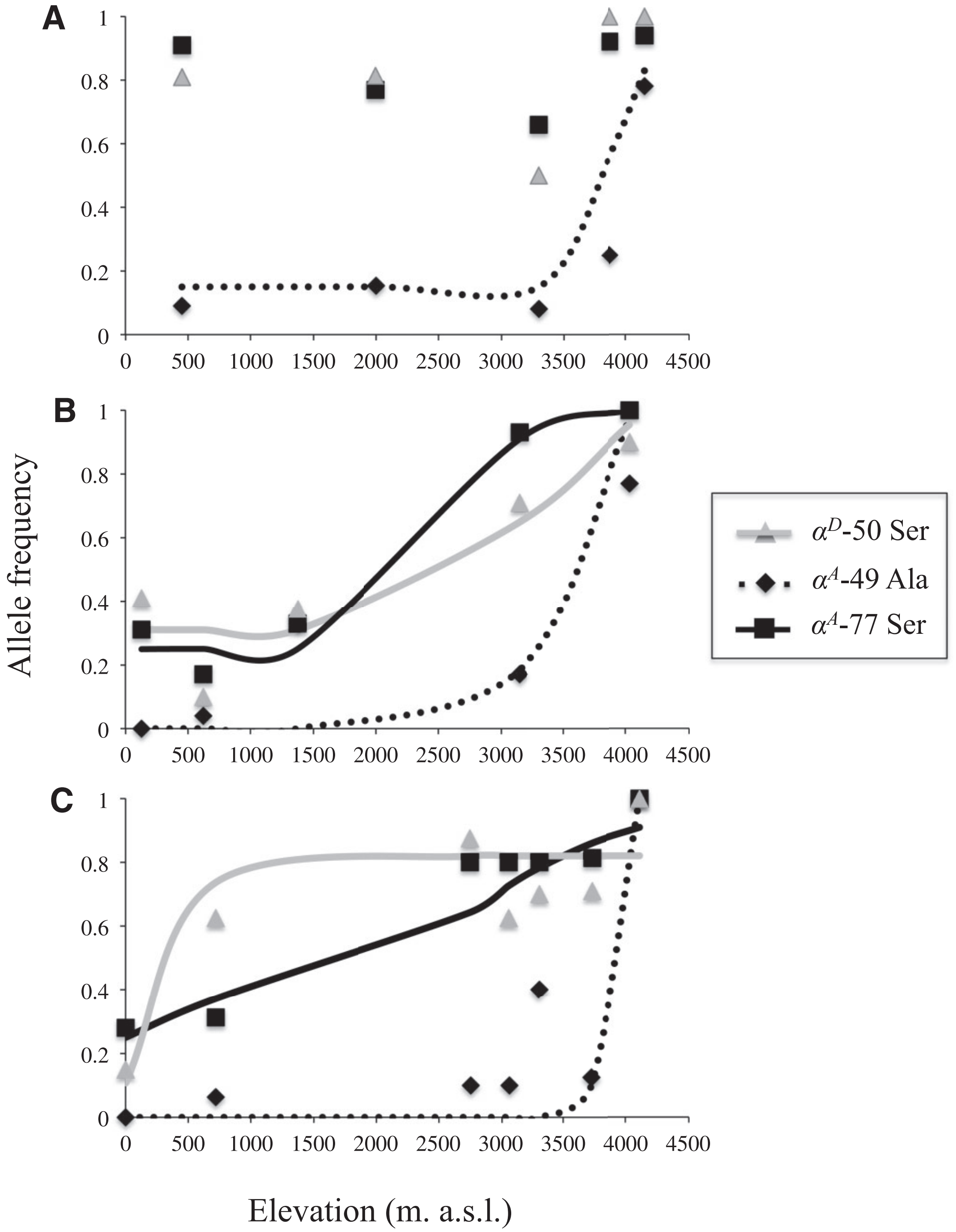

Fig. 3. Altitudinal clines in allele frequency for amino acid polymorphisms in the α^A- and α^D-globin genes of *Zonotrichia capensis*: (*A*) Transect T1, (*B*) transect T2, and (*C*) transect T3 (see fig. 1). Curves represent maximum-likelihood estimates of cline shape parameters for the derived allele at each polymorphic site (see Materials and Methods). Cline shape parameters are listed in table 3.

erythrocytes [Petschow et al. 1977]; fig. 7*A* and *B*). In the case of HbD, there was no difference in intrinsic O_2-affinity between the highland and lowland variants (values of $P_{50(\text{stripped})}$ were essentially identical), nor was there a detectable difference in the presence of Cl^- ions, but the highland variant exhibited a slightly increased O_2-affinity in the presence of IHP ($P_{50(\text{IHP, }37^\circ\text{, pH 7.4})} = 26.69 \pm 1.43$ vs. 31.58 ± 0.96 torr, respectively) and in the simultaneous presence of Cl^- and IHP ($P_{50(\text{KCl+IHP, }37^\circ\text{, pH 7.4})} = 18.56 \pm 0.86$ vs. 22.42 ± 1.02 torr, respectively; fig. 7*C*). A comparison of log-transformed differences in P_{50} values in the presence and absence of anionic effectors (fig. 7*D*) revealed that the highland HbD variant was characterized by a slightly lower IHP sensitivity compared with the lowland variant ($\Delta\log P_{50(\text{IHP-str})} = 1.15$ vs. 1.24, respectively; $\Delta\log P_{50([\text{KCl+IHP}]\text{-str})} = 1.00$ vs. 1.10, respectively).

Downloaded from http://mbe.oxfordjournals.org/ at University of Nebraska-Lincoln Libraries on August 29, 2014

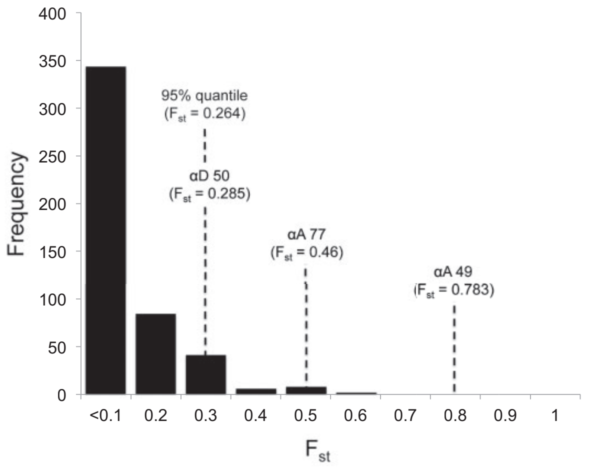

Fig. 4. Frequency histogram of F_{st} values for 483 RAD polymorphisms that map to transcripts of putative *Zonotrichia* protein-coding genes. F_{st} values are binned in 0.1 increments with the bin value representing the maximum F_{st} value for all loci included within it.

Protein Engineering Isolates the Functional Effects of Individual α^A-Globin Mutations

Although the analysis of native HbA variants revealed no significant net difference in HbA O_2-affinity between alternative double homozygotes at the two-variable α^A sites, "49Ala–77Ser" and "49Gly–77Ala" (fig. 7A), it is possible that the absence of a detectable combined effect could conceal significant individual effects of the mutations that are only manifest in one or both of the constituent single-mutant genotypes '49Gly–77Ser' and/or '49Ala–77Ala'. Such epistatic effects would be biologically relevant because both single-mutant genotypes are present at intermediate frequencies at midelevations. To test for offsetting effects of the two α^A mutations (i.e., partial or reciprocal sign-epistasis; Weinreich et al. 2005; Poelwijk et al. 2007), we used site-directed mutagenesis to synthesize rHb mutants representing each of the four possible genotypic combinations: The most common lowland genotype (the ancestral "49Gly–77Ala"), the most common highland genotype (the derived double-mutant "49Ala–77Ser"), and each of the alternative single-mutant intermediates ("49Gly–77Ser" and "49Ala–77Ala"). Functional analysis of the purified rHb mutants revealed no significant additive or epistatic effects of mutations at the two sites and, consistent with the analysis of the native Hb variants, the experiments revealed no significant difference in O_2-affinity between the highland and lowland genotypes (table 5).

In summary, the HbA isoforms of high- and low-altitude sparrows did not exhibit discernible differences in O_2-binding properties, and site-directed mutagenesis experiments confirmed that the two clinally varying α^A polymorphisms did not contribute to changes in Hb-O_2 affinity, either singly or in combination.

Discussion

Recent years have witnessed a surge of interest in the use of DNA sequence variation to identify genetic targets of positive

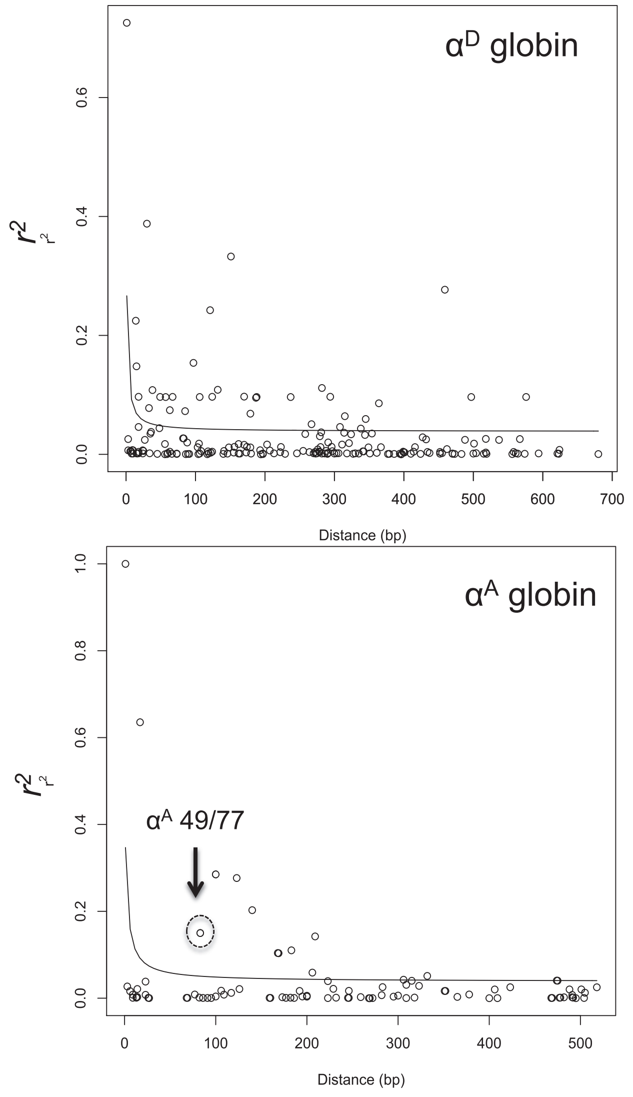

Fig. 5. Rates of decay of linkage disequilibrium across α^A- and α^D-globin coding regions.

selection. Although neutrality tests based on polymorphism and/or divergence data are useful for generating hypotheses about the adaptive significance of particular mutational changes, such hypotheses should be tested with functional experiments. In the absence of experimental validation, analyses of variation in coding sequence can lead to facile inferences about the adaptive significance of observed amino acid replacements. This is especially true in studies of candidate genes in natural populations where there is some plausible a priori expectation about the adaptive significance of changes in protein function.

In this study, we have shown that altitudinal clines in Hb polymorphisms that could be interpreted as reflecting local adaptation are not associated with any discernible variation in oxygenation properties. In the absence of experimental data, it would have been tempting to conclude that the dramatic altitudinal shifts in allele frequency at α^A49 and α^A77 reflect a history of spatially varying selection on the respiratory properties of Hb in *Z. capensis* populations that are native to different elevations. Such an interpretation would seem

Downloaded from http://mbe.oxfordjournals.org/ at University of Nebraska-Lincoln Libraries on August 29, 2014

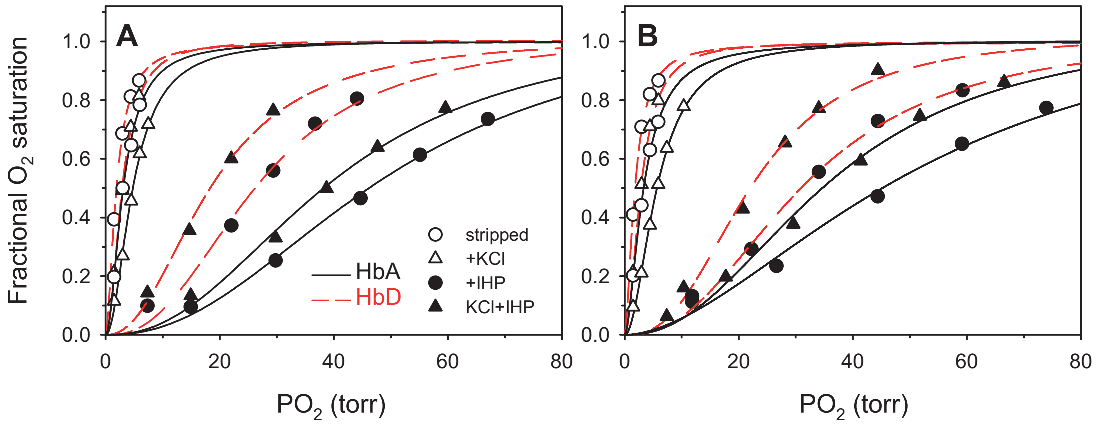

Fig. 6. Oxygen-equilibrium curves for purified HbA and HbD isoforms from highland and lowland *Zonotrichia capensis* ([*A*] and [*B*], respectively) measured at pH 7.4 and 37 °C in the absence (stripped) and presence of allosteric effectors ([Cl-], 0.1 M; [HEPES], 0.1 M; IHP/Hb tetramer ratio, 2.0; [Heme], 0.300 mM).

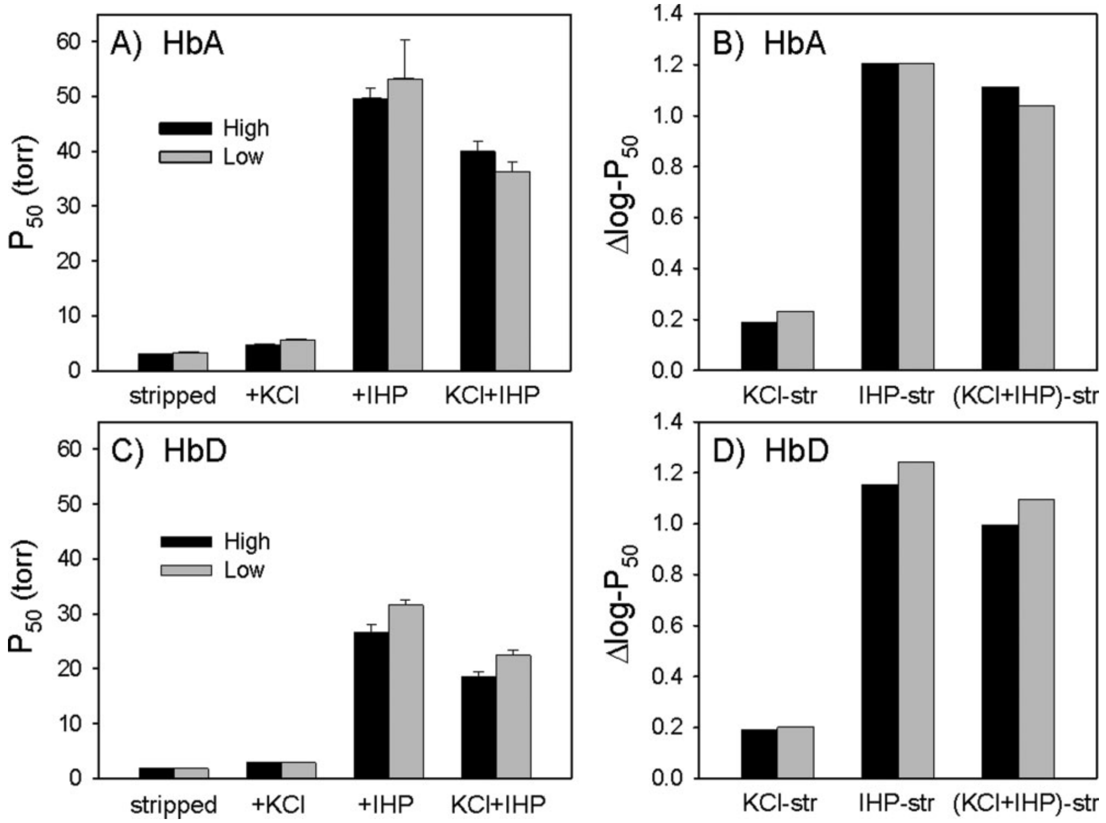

Fig. 7. Oxygenation properties of HbA and HbD isoforms from high- and low-altitude populations of *Zonotrichia capensis*. (*A*) P_{50} values (means ± SEM) for purified HbA variants of highland and lowland sparrows measured at pH 7.4 and 37 °C in the absence (stripped) and presence of allosteric effectors ([Cl^-], 0.1 M; [HEPES], 0.1 M; IHP/Hb tetramer ratio, 2.0; [Heme], 0.300 mM). (*B*) Log-transformed differences in P_{50} values of highland and lowland HbA variants in the presence and absence of allosteric effectors. The $\Delta \log P_{50}$ values measure the extent to which Hb-O_2 affinity is reduced in the presence of a given allosteric effector (Cl^-, IHP, or both anions together). (*C*) P_{50} values for HbD variants of highland and lowland sparrows (experimental conditions as above). (*D*) $\Delta \log P_{50}$ values for HbD variants of highland and lowland sparrows.

plausible given that evolved increases in Hb-O_2 affinity have been documented in other vertebrate taxa that are native to high altitude (Weber et al. 2002; Storz 2007; Weber 2007; Storz and Moriyama 2008; Storz et al. 2009; Storz, Runck, et al. 2010; Projecto-Garcia et al. 2013; Revsbech et al. 2013). The absence of detectable functional effects of amino acid replacements at sites α^A49 and α^A77 in the HbA isoform of *Z. capensis* is consistent with studies of human Hb mutations at the same sites, Hb Savaria (α49Ser→Arg) and Hb Guizhou (α77Pro→Ala), neither of which have altered O_2-affinities relative to normal human Hb (Szelenyi et al. 1980; Hattori et al. 1985).

Downloaded from http://mbe.oxfordjournals.org/ at University of Nebraska-Lincoln Libraries on August 29, 2014

Table 4. O_2-Affinities (P_{50}, torr; mean ± SEM) and Cooperativity Coefficients (n_{50}; mean ± SEM) for Purified HbA and HbD isoHbs Measured in 0.1 M HEPES Buffer at pH 7.40, 37 °C.

	Highland HbA (49Ala–77Ser)	Lowland HbA (49Gly–77Ala)	Highland HbD (50Ser)	Lowland HbD (50Pro)
P_{50} (torr)				
Stripped	3.09 ± 0.11	3.34 ± 0.06	1.88 ± 0.03	1.80 ± 0.06
KCl	4.78 ± 0.14	5.69 ± 0.15	2.90 ± 0.08	2.87 ± 0.04
IHP	49.57 ± 2.04	53.18 ± 7.23	26.69 ± 1.43	31.58 ± 0.96
KCl + IHP	39.98 ± 1.82	36.28 ± 1.85	18.56 ± 0.86	22.42 ± 1.02
n_{50}				
Stripped	1.87 ± 0.12	1.72 ± 0.05	1.71 ± 0.05	1.71 ± 0.09
KCl	1.99 ± 0.11	1.97 ± 0.09	2.02 ± 0.09	1.96 ± 0.05
IHP	2.25 ± 0.17	1.78 ± 0.03	2.44 ± 0.36	2.39 ± 0.15
KCl + IHP	2.31 ± 0.23	2.26 ± 0.02	2.22 ± 0.22	2.58 ± 0.28

NOTE.—Measurements were conducted in the absence of anionic effectors (stripped), in the presence of 0.1 M KCl or IHP (IHP/Hb tetramer ratio = 2.0), and in the presence of both effectors. [Heme], 0.300 mM.

It remains possible that the observed amino acid polymorphisms in *Z. capensis* Hbs could have effects on fitness that are not directly related to respiratory functions of Hb. For example, an intensively studied two-locus β-globin polymorphism in house mice (genus *Mus*) shows all the hallmarks of long-term balancing selection (Storz et al. 2007; Runck et al. 2009, 2010), and it was hypothesized that allelic variation in Hb-O_2 affinity might impinge on some fitness-related aspect of physiological performance (Berry 1978). This hypothesis was rejected by functional experiments on purified β-chain isoforms, which revealed no allelic differences in oxygenation properties (Runck et al. 2010; Storz et al. 2012). However, allelic β-chain differences in reactive cysteine content contribute to variation in the blood-mediated metabolism of oxidants and thiol reactants such as nitric oxide and glutathione, thereby influencing cellular responses to oxidative and nitrosative stress (Giustarini et al. 2006; Hempe et al. 2007; Storz et al. 2012). This example and the many recently discovered "moonlighting" functions of enzymes in intermediary metabolism (Marden 2013) demonstrate the importance of maintaining a wide field of vision when considering the possible functional significance of protein polymorphism. However, in contrast to the house mouse Hbs, the nature of the amino acid variation in the *Z. capensis* Hbs does not suggest the potential for similar effects on intraerythrocytic redox status. Thus, we conservatively conclude that the amino acid variation in *Z. capensis* Hb is physiologically inconsequential with respect to blood-O_2 transport.

Our experimental results therefore leave us with two possible alternatives that require further testing: (1) The outlier F_{st} values are false positives and altitudinal clines in allele frequency at the α^A- and α^D-globin genes simply reflect the interplay between genetic drift and gene flow, or 2) the altitudinal clines reflect the indirect effects of divergent selection on closely linked noncoding sites (e.g., *cis*-regulatory elements in immediately upstream or downstream flanking regions that were not included in our survey of sequence variation).

Table 5. O_2-Affinities (P_{50}, torr; mean ± SEM) and Cooperativity Coefficients (n_{50}; mean ± SEM) for Purified rHb Mutants Measured in 0.1 M HEPES Buffer at pH 7.40, 37 °C.

	49G–77A	49A–77A	49G–77S	49A–77S
P_{50} (torr)				
Stripped	2.70 ± 0.05	2.63 ± 0.06	2.88 ± 0.03	2.80 ± 0.03
KCl	3.75 ± 0.06	3.57 ± 0.06	4.18 ± 0.02	3.88 ± 0.13
IHP	27.75 ± 1.15	26.47 ± 1.21	30.85 ± 0.83	28.66 ± 1.00
KCl + IHP	21.64 ± 0.99	20.03 ± 0.83	24.41 ± 0.68	23.40 ± 1.22
n_{50}				
Stripped	1.46 ± 0.03	1.49 ± 0.05	1.51 ± 0.02	1.54 ± 0.03
KCl	1.59 ± 0.04	1.57 ± 0.05	1.91 ± 0.02	1.69 ± 0.08
IHP	1.59 ± 0.10	1.48 ± 0.09	1.65 ± 0.08	1.48 ± 0.07
KCl + IHP	1.56 ± 0.09	1.71 ± 0.14	1.83 ± 0.09	1.58 ± 0.11

NOTE.—Measurements were conducted in the absence of anionic effectors (stripped), in the presence of 0.1 M KCl, in the presence of IHP (IHP/Hb tetramer ratio = 2.0), and in the presence of both effectors. [Heme], 0.300 mM. The 49 G–77 A genotype represents the most common two-site α^A-globin genotype in lowland sparrows, and the double-mutant 49 A–77S represents the most common two-site genotype in highland sparrows. The single-mutant genotypes, 49 A–77 A and 49 G–77 S, are present at intermediate frequencies at middle elevations.

The altitudinal patterning of the α^A 77Ala/Ser polymorphism in *Z. capensis* mirrors that reported for an α^A 77Ala/Thr replacement change that is shared by several independent lineages of high-altitude waterfowl (McCracken et al. 2009). In several different species of ducks and sheldgeese, the ancestral α^A 77Ala allele predominated in birds sampled from lowland localities, whereas a derived α^A 77Thr allele predominated in conspecific populations or sister species native to high altitudes. The functional effects of these parallel amino acid changes were not experimentally tested, but the authors suggested that the parallel amino acid replacements may contribute to parallel adaptation of Hb function (McCracken et al. 2009). McCracken et al. (2009) hypothesized that replacing the ancestral α^A 77Ala residue with Thr (which, like Ser, has a side chain with a polar –OH group) may increase Hb-O_2 affinity by reducing the binding affinity for the negatively charged inositol pentaphosphate at a putative oxygenation-linked polyanion-binding site formed by a cluster of charged residues at the N- and C-termini of the α-chains (Tamburrini et al. 2000; Riccio et al. 2001). Our experiments involving native and rHb mutants demonstrate that the α^A77Ala→Ser replacement has no demonstrable effect on Hb-O_2 affinity or phosphate sensitivity (tables 4 and 5; figs. 6*A* and *B* and 7*A* and *B*). This is consistent with the external position of the α77 residue at the edge of the central cavity (Fermi and Perutz 1981). If the α^A77Ala→Ser replacement in sparrow Hb has the same effect as the physiochemically similar α^A 77Ala→Thr replacement in ducks and sheldgeese, then our experimental results predict that the observed substitutions in the HbA isoforms of these waterfowl species are inconsequential with respect to blood-O_2 transport.

Insights into Structure–Function Relationships

The highland variant of sparrow HbD was characterized by a reduced sensitivity to IHP compared with the lowland variant.

Downloaded from http://mbe.oxfordjournals.org/ at University of Nebraka-Lincoln Libraries on August 29, 2014

IHP has the effect of reducing Hb-O_2 affinity by electrostatically binding and stabilizing deoxy Hb in the "T-state" quaternary structure. In avian Hbs, oxygenation-linked IHP-binding involves charge–charge interactions with seven highly conserved β-chain residues that line the opening of the central cavity: Val1, His2, Lys82, Arg135, Val136, His139, Arg143 (Arnone and Perutz 1974; Tamburrini et al. 2000). Given that the known oxygenation-linked phosphate-binding sites are restricted to the β-chain subunit, the observed difference in IHP-sensitivity between the highland and lowland HbD variants is intriguing (fig. 7*C* and *D*). The implication is that the α^D50Pro→Gly replacement must indirectly perturb phosphate-binding at the β–β interface. Indirect effects of α-chain amino acid replacements on phosphate-sensitivities have also been documented in the Hbs of mice (Natarajan et al. 2013) and turtles (Damsgaard et al. 2013), suggesting that long-range, second-order perturbations of Hb allostery may be common.

Conclusion

It is clear that *Z. capensis* is able to tolerate a broad range of elevations without any locally adaptive evolutionary changes in the oxygenation properties of Hb. In contrast, comparative data for Andean hummingbirds demonstrate a strong positive association between Hb-O_2 affinity and native elevation that is attributable to parallel affinity-altering substitutions in multiple lineages (Projecto-Garcia et al. 2013). Likewise, the bar-headed goose—renowned for trans-Himalayan migratory flights—has evolved an elevated Hb-O_2 affinity relative to lowland congeners and other geese (Jessen et al. 1991; Weber et al. 1993). Whether highland bird species have typically evolved different Hb-O_2 affinities relative to lowland sister taxa remains an open question—a question that can only be answered by systematic comparative studies that include experimental data. Another open question concerns the role of regulatory adjustments in intraerythrocytic Hb isoform composition as a mechanism for optimizing blood-O_2 affinity to prevailing conditions. As avian HbD has a higher O_2-affinity than HbA (Grispo et al. 2012), intracellular changes in the HbA/HbD ratio could potentially produce substantial changes in blood-O_2 affinity (Weber et al. 1988; Hiebl et al. 1989; Hoffmann and Storz 2007). The HbA/HbD ratio showed slight (but nonsignificant) differences between highland and lowland *Z. capensis*. In Andean hummingbirds, interspecific variation in the HbA/HbD ratio exhibited no association with native elevation (Projecto-Garcia et al. 2013). Studies of additional avian taxa are needed to assess the importance of this potential mechanism for modulating blood-O_2 affinity.

Maintenance of adequate tissue O_2 supply in animals exposed to altitudinal hypoxia depends on an ensemble of adaptive responses, which include long-term, evolutionary changes in anatomical and molecular structures, as well as short-term nongenetic adjustments that rapidly modulate the flux capacity of O_2 transport (Storz, Scott, et al. 2010). The absence of genetically based structural adaptations that affect Hb function in *Z. capensis* may reflect a general strategy of enhanced physiological plasticity to cope with changes in O_2 availability across its broad elevational distribution (Cheviron et al. 2008). On the steep western slope of the Peruvian Andes, ambient O_2 tensions vary dramatically over relatively short geographic distances. This fine-scaled environmental variation may be expected to select for phenotypic plasticity over genotypic specialization in species with broad elevational distributions (Cheviron et al. 2008, 2013, 2014; Tufts et al. 2013).

Current research in evolutionary genetics relies heavily on indirect inferences drawn from statistical analyses of DNA sequence variation, but studies that buttress inferences of positive selection and adaptation with rigorous experimental evidence are rare. Results of our study of Hb polymorphism in *Z. capensis* highlight the importance of experimentally validating the hypothesized effects of genetically based changes in protein function to avoid the pitfalls of adaptive storytelling. In addition to raising evidentiary standards for inferring molecular adaptation, the integration of functional experiments with population genetic analyses can also provide valuable mechanistic insights that enrich our understanding of evolutionary pattern and process (Dean and Thornton 2007; Dalziel et al. 2009; Storz and Wheat 2010; Barrett and Hoekstra 2011; Storz and Zera 2011; Yokoyama 2012; Harms and Thornton 2013).

Materials and Methods

Sampling

Voucher specimens of *Z. capensis* (*n* = 197) and tissue samples (supplementary tables S1 and S2, Supplementary Material online) were deposited in the ornithological collections of the Louisiana State University Museum of Natural Science (Baton Rouge), the Museo de Historia Natural, Universidad Nacional Mayor de San Marcos (Lima, Peru), the Centro de Ornitología y Biodiversidad (Lima, Peru), and/or the Museum of Southwestern Biology of the University of New Mexico (Albuquerque).

Determination of Hb Isoform Composition

We used a combination of IEF (PhastSystem; GE Healthcare Bio-Sciences, Piscataway, NJ) and MS/MS to characterize Hb isoform composition in the mature erythrocytes of 29 *Z. capensis* specimens. After separating native Hbs by means of IEF, gel bands were excised and digested with trypsin. The resultant peptides were then identified by means of MS/MS. The peak lists of the MS/MS data were generated by Distiller (Matrix Science, London, UK) using the charge state recognition and deisotoping with default parameters for quadrupole time-of-flight data. Database searches of the resultant MS/MS spectra were performed using Mascot (Matrix Science, v1.9.0, London, UK). Specifically, peptide mass fingerprints derived from the MS/MS analysis were used to query a custom database of avian α- and β-globin sequences. These amino acid sequences were derived from conceptual translations of the adult α^A-, α^D-, and β^A-globin genes of *Z. capensis*, in addition to the full complement of embryonic and adult α- and β-like globin genes that have been annotated in the genome assemblies of zebra finch (*Taeniopygia guttata*), chicken (*Gallus gallus*), turkey (*Melagris gallopavo*), and

Downloaded from http://mbe.oxfordjournals.org/ at University of Nebraka-Lincoln Libraries on August 29, 2014

mallard duck (*Anas platyrhynchos*), and other amniote vertebrates (Hoffmann et al. 2010, 2011, 2012; Storz et al. 2011). The following search parameters were used for the MS/MS analysis: No restriction on protein molecular weight or isoelectric point, and methionine oxidation allowed as a variable peptide modification. Mass accuracy settings were 0.15 Da for peptide mass and 0.12 Da for fragment ion masses. We identified all significant protein hits that matched more than one peptide with $P < 0.05$. After separating the HbA and HbD isoforms by native gel IEF and identifying each band on the gel by MS/MS, the relative abundance of the different isoforms was quantified densitometrically using Image J (Abramoff et al. 2004).

Sanger Sequencing of Globin Genes

Sequence data for the α^A- and α^D-globin genes were generated for a total of 163 individual specimens that were collected along the replicate elevational transects (fig. 1). Because a preliminary survey of β^A-globin variation in 30 individuals (15 high elevation and 15 low elevation) did not exhibit high levels of differentiation between the endpoints of the altitudinal transects, this gene was not included in additional surveys of nucleotide variation at intermediate elevations. Sequence data for the α^A-, α^D-, and β^A-globin have been deposited in GenBank (accession numbers KM246945-KM247263).

For the population genetic analyses of the adult-expressed globin genes, we extracted total genomic DNA from pectoral muscle using DNeasy tissue extraction kits (Qiagen, Valencia, CA) or standard phenol chloroform protocols. All polymerase chain reactions (PCRs) were performed in 25 μl volumes with 0.1 μl of either AmpliTaq Gold Taq Polymerase or Invitrogen Taq DNA polymerase, 2.5 μl 10 $\times$ Tris buffer with $MgCl_2$, 1.5 μl dNTP mix (each dNTP 50 μM), 1.5 μl of each primer (10 μM), and approximately 50 ng of template DNA. Thermocycling profiles were as follows: An initial denaturing step at 94 °C for 2 min, followed by 35 cycles of a 30-s denaturing step at 94 °C, a 30-s annealing step at a locus-specific annealing temperature, and 1-min extension step at 72 °C. A final extension step was carried out at 72 °C for 10 min. Primer sequences and PCR annealing temperatures for the amplification of globin genes are provided in supplementary table S3, Supplementary Material online. All PCR products were purified using Alkaline Phosphatase (CIP) (New England Biolabs #M0290L) and Exonuclease I (*Escherichia coli*) (New England Biolabs #M0293L), by adding 0.5 μl of each enzyme per 5 μl of PCR product and incubated at 37 °C for 30 min followed by an 80 °C for 20 min. Purified PCR products were sequenced in both directions using Big Dye Terminator chemistry (Applied Biosystems, Foster City, CA).

For the subset of specimens used in the functional analyses of Hb-O_2 affinity, we isolated RNA from packed red cells and generated cDNA sequences for the α^A-, α^D-, and β^A-globin genes (supplementary table S2, Supplementary Material online). We extracted total RNA using TRI-reagent (Sigma #T9424) and BCP phase separation reagent (Fisher #BP151) following manufacturer protocols, and we amplified the globin genes using a Qiagen OneStep RT-PCR kit. All one-step reverse transcription (RT)-PCRs were performed in 25 μl volumes following manufacturer protocols, with the locus-specific annealing temperatures listed in supplementary table S3, Supplementary Material online. RT-PCR products were also purified using Alkaline Phosphatase (CIP) and Exonuclease I (*E. coli*), and sequenced using BigDye Terminator chemistry.

Multiple individuals were heterozygous for an indel polymorphism in one or more of the globin genes. For these individuals, we cloned diploid globin gene amplicons using a TOPO TA Cloning Kit (Invitrogen #K4575-40) following manufacturer protocol. For each individual specimen, we amplified and sequenced inserts from six to eight positive clones per gene using T3 and T7 vector primers.

Population Genetic Analyses

Sequences from both strands were aligned and edited using SEQUENCHER, version 5.0.1 (Genecodes, Ann Arbor, MI). For each of the sequenced loci (α^A-, α^D-, and β^A-globin genes), we inferred the haplotype phase of heterozygous sites using the program PHASE (Stephens et al. 2001; Stephens and Donnelly 2003). All of the PHASE analyses were run for 10,000 steps with a 1,000-step burn-in and a thinning interval of 10. We computed summary statistics of nucleotide polymorphism and LD using DnaSP v. 5.0 (Librado and Rozas 2009). To characterize levels of nucleotide variation in the globin genes, we calculated nucleotide diversity, π, and Watterson's θ_W, an estimator of population mutation rate ($= 4N\mu$, where N is the effective population size and μ is the mutation rate per nucleotide). We calculated Tajima's (1989) D to characterize the distribution of allele frequencies at silent sites, and we calculated F_{st} for individual amino acid polymorphisms using the program Arlequin (Excoffier et al. 2005). To characterize the levels of intralocus LD, we computed Hudson's (1987) estimator of the scaled recombination rate, $4Nc$, where c is the rate of crossing-over between adjacent nucleotides, and Kelly's (1997) Z_{nS}, which provides a measure of LD based on the average squared correlation in allele frequencies (r^2) between pairs of sites. To test whether measured values of Tajima's D and Z_{nS} deviated from neutral-equilibrium expectations, we obtained critical values for each statistic by conducting 10,000 coalescent simulations (no recombination) that were conditioned on the number of segregating sites. We measured the rate of decay of intragenic LD as a function of physical distance under a recombination–mutation–drift model (Hill and Weir 1988) by means of nonlinear regression, using the *nls* function in R (R Core Team 2012).

We tested for differences in locus-specific migration rates between the elevational and control transects for α^A- and α^D-globin using the program IMa (Hey and Neilsen 2007). We restricted all of the IMa analyses to populations sampled from the endpoints of each transect. This allowed us to estimate levels of gene flow across each transect, while avoiding the transition zone between high and low altitude populations

Downloaded from http://mbe.oxfordjournals.org/ at University of Nebraka-Lincoln Libraries on August 29, 2014

along the elevational transects (Cheviron and Brumfield 2009). For each transect, we estimated the parameter m (rate of migration scaled by the neutral mutation rate μ). To obtain a single estimate of migration rate across each sample transect, we constrained m to be symmetric in all analyses.

Because IMa assumes the absence of intralocus recombination, we restricted the IMa analyses to the largest nonrecombining block of sequence that included the altitudinally varying amino acid polymorphisms in the α^A- and α^D-globin genes. Intralocus recombination was detected using the four-gamete test (Hudson and Kaplan 1995) implemented in DNAsp v. 5.10.1 (Librado and Rozas 2009). For each IMa analysis, we performed initial runs with large, flat priors for each of the model parameters, and we used the results of these runs to define priors that encompassed the entire posterior probability distribution of each parameter estimate. Using these priors, we ran two replicate runs for each transect comparisons.

Because we observed altitudinal shifts in allele frequencies for both the α^A- and α^D-globin genes along the elevational transects, we fit maximum-likelihood clines to the allele frequency data to estimate four parameters—cline center (c), cline width (w), and the allele frequencies at the ends of the cline (p_{min} and p_{max}). We used the following search parameters in the program ClineFit (Porter et al. 1997): A burn-in of 300 parameter tries per step, and then after the burn-in, 2,000 replicates were saved with 30 replicates run between saves. We statistically assessed cline concordance (equal cline widths) and coincidence (equal cline centers) between the two α-globin genes using two log-likelihood support limits (ln L_{max} − 2), which are analogous to 95% confidence intervals (Edwards 1972).

Surveying Genome-Wide Differentiation with a Genotyping-by-Sequencing Approach

To more broadly survey patterns of genomic differentiation between high- and low-elevation populations, we produced multiplexed, reduced-representation Illumina libraries following Parchman et al. (2012). Briefly, we digested genomic DNA samples for a total of 30 individuals (15 from high elevation and 15 from low elevation; supplementary table S1, Supplementary Material online) with two restriction endonucleases (*Eco*RI and *Mse*I), and then ligated double-stranded adaptor oligonucleotides that contained Illumina sequencing binding sites and a unique 10-bp barcode for individual identification. We used PCR to amplify the adaptor-ligated fragments. Details on the adaptor sequences as well as the digestion and PCR conditions can be found in Parchman et al. (2012). Following amplification, barcoded amplicons from each individual were pooled in equimolar concentrations and electrophoresed on 2% agarose gel for size selection. We excised fragments that were between 350 and 500 bp in length and purified these gel-extracted fragments using a Qiaquick gel extraction kit (Qiagen Inc.). The pooled library was sequenced in a single lane on the Illumina HiSeq 1000 platform as 100-nt single-end reads at the National Center for Genome Research (Santa Fe, NM), producing an average of 1.67 million reads/individual (range 697,458–2,899,942) (supplementary table S4, Supplementary Material online).

Sequences were parsed by individual barcodes and trimmed of adaptor sequences and low quality bases using custom Perl scripts (Parchman T, personal communication) resulting in a final mean read length of 87 nt. To limit our analysis of sequence polymorphism within putative *Zonotrichia* protein-coding genes, we first mapped individual reads to the published transcriptome of a closely related congener, *Z. leucophrys* (Balakarishinan et al. 2014), using the "−sensitive-local" settings in Bowtie2 (Langmead and Salzberg 2012). On average, 19% of the reads mapped to known transcripts (supplementary table S4, Supplementary Material online). Aligned reads were then processed using the program STACKS (Catchen, Hohenlohe, et al. 2013) to identify single-nucleotide polymorphisms (SNPs) in reads that mapped to transcripts using the following input parameters for pstacks: - m3, −model_type snp, −alpha 0.05. All 30 individuals were included when compiling the SNP catalog in cstacks. We restricted downstream analysis to loci that were genotyped in at least eight individuals per population with a minimum sequencing depth of 10 reads/locus/individual and a minor allele frequency (MAF) of 0.05, resulting in a final data set of 483 unique loci. We calculated locus-specific F_{st} values using the program POPULATIONS implemented in STACKS (Catchen, Bassham, et al. 2013). Parsed Illumina reads have been deposited in the NCBI short read archive (SRA PRJNA255680).

Measurement of Hb-O_2 Affinity

In order to isolate the effects of mutations at sites 49 and 77 in the α^A-globin gene, we screened coding sequences in the α^A-, α^D-, and β^A-globin genes to identify sets of highland and lowland specimens with informative multilocus genotypes. For the highland genotype, we pooled blood samples from three individual specimens that were "α^A-49Ala–77Ser" double-homozygotes, and for the lowland genotype we pooled samples from six specimens that were alternative "α^A-49Gly–77Ala" double homozygotes. By obtaining diploid sequence data for each of the three postnatally expressed globin genes, we confirmed that these nine specimens harbored no additional amino acid mutations in the α- or β-type subunits that could confound the comparison between the two-site α^A genotypes.

For each of the pooled samples, the HbA and HbD isoforms were separated and were stripped of organic phosphates and other anions by ion-exchange fast-protein liquid chromatography (FPLC), using a HiTrap QHP column (GE Healthcare), as described by Projecto-Garcia et al. (2013). O_2-equilibrium curves were measured on 3 μl thin-film samples (0.3 mM heme concentration), at 37 °C, 0.1 M HEPES buffer (pH 7.4). Using standard experimental conditions (Imai 1982; Mairbaurl and Weber 2012), we measured oxygenation properties of purified Hb solutions under four treatments: 1) In the absence of allosteric effectors (stripped), 2) in the presence of 0.1 M Cl^- (in the form of KCl), 3) in the

Downloaded from http://mbe.oxfordjournals.org/ at University of Nebraska-Lincoln Libraries on August 29, 2014

presence of IHP (IHP/Hb tetramer ratio = 2.0), and 4) in the simultaneous presence of both effectors. The curves were measured using a modified O_2 diffusion chamber and absorption at 436 nm was monitored while subjecting thin-film samples to varying O_2 tensions of gas mixtures (prepared using Wösthoff gas-mixing pumps that perfuse the chamber; Weber 1981, 1992; Weber et al. 2004). P_{50} (O_2 tension at half-saturation) and n_{50} (Hill's cooperativity coefficient at half-saturation) values were obtained by fitting the Hill equation $Y = PO_2{}^n/(P_{50}{}^n + PO_2{}^n)$ to the experimental O_2 saturation data using a nonlinear regression model (Y, fractional saturation; PO_2, partial pressure of O_2; n, cooperativity coefficient). Free Cl^- concentrations were controlled with a model 926S Mark II chloride analyzer (Sherwood Scientific Ltd, Cambridge, UK).

Vector Construction and Site-Directed Mutagenesis

The α^A- and β^A-globin genes of *Z. capensis* were synthesized by Genscript (Piscataway, NJ) after optimizing nucleotide sequences to match *E. coli* codon preferences. The pGM-RCS plasmid contained tandemly cloned copies of the α- and β-globin genes along with the *methionine aminopeptidase* (MAP) gene, as described by Natarajan et al. (2011, 2013). To maximize efficiency in the posttranslational cleaving of N-terminal methionines from the nascent α- and β-chain polypeptides, an additional copy of the MAP gene was cloned into the pCO-MAP plasmid and was coexpressed with a *kanamycin-resistant* gene. The JM109 (DE3) cells transformed with both the pGM-RCS and pCO-MAP plasmids were subject to dual selection on ampicillin/kanamycin LB plates.

The mutagenesis experiments were performed using the QuikChange® II XL Site-Directed Mutagenesis kit from Stratagene (LaJolla, CA) as per the manufacturer's protocol. The lowland α^A-globin genotype "49Gly–77Ala" was converted to the highland genotype "49Ala–77Ser" by engineering two codon changes through site-directed mutagenesis. The same procedure was used to engineer the two single-mutant intermediates between the highland and lowland genotypes ("49Ala–77Ala" and "49Gly–77Ser"). Each engineered codon change was verified by sequencing the plasmid DNA.

Expression and Purification of rHbs

Large-scale production of rHb was conducted in 1–1.5 l batches of TB medium. Bacterial cultures were grown at 37 °C in an orbital shaker at 200 rpm until the optical density reached 0.6–0.8 at 600 nm. The cells were induced by 0.2 mM IPTG and supplemented with hemin (50 μg/ml) and glucose (20 g/l). The cells were then grown at 28 °C for 16 h in an orbital shaker at 200 rpm. The bacterial cultures were saturated with CO gas for 15 min and the cells were harvested by centrifugation. The cell pellets were stored in −80 °C overnight and were then resuspended in lysis buffer (3 ml/g of cells, 50 mM Tris base, 1 mM ethylenediaminetetraacetic acid [EDTA], 0.5 mM dithiothreitol, lysozyme 1 mg/g of cells) and sonicated. Polyethyleneimine solution (0.5–1%) was added to the crude lysates to precipitate the nucleic acids. The rHbs were purified by two-step ion exchange chromatography using FPLC. The clarified crude lysate was dialyzed overnight at 4 °C against CO equilibrated Tris buffer (20 mM Tris–HCl, 0.5 mM EDTA, pH 8.6). The dialyzed lysate was centrifuged at 13,000 rpm for 20 min at 4 °C and the supernatant was subjected to anion exchange chromatography by passing it through a HiTrap™ Q Sepharose Fast Flow column (GE Healthcare 17-5156-01). Eluted rHb solutions were concentrated by centrifugal filtrate (Amicon Ultra-4, 30,000 MW) and dialyzed over night at 4 °C against CO-equilibrated Na_3PO_4 buffer (10 mM, pH 7.0). The dialyzed samples were then purified by cation exchange chromatography using HiTrap™ SP Sepharose Fast Flow column (GE Healthcare 17-5157-01). The bound rHb was eluted with a linear gradient of 20 mM sodium phosphate buffer, pH 8.0. The eluted rHb fractions were concentrated by using centrifugal filtrate and stored in −80 °C. The purified rHbs were analyzed by 18% sodium dodecyl sulphate–polyacrylamide gel electrophoresis and by IEF on a PhastGel IEF 3-9 (GE Healthcare #17-0543-01). Absorbance spectra of oxyHb, deoxyHb, and CO derivatives were measured at 450–600 nm to confirm that the absorbance maxima of rHb mutants correspond to those of the native Hbs.

Supplementary Material

Supplementary tables S1–S4 are available at *Molecular Biology and Evolution* online (http://www.mbe.oxfordjournals.org/).

Acknowledgments

The authors thank the Peruvian government agencies INRENA and DGFFS for permits (042-2004-INRENA-IFFS-DCB, 012C/C-2005-INRENA-IANP, 004-2007-INRENA-IFFS-DCB, 135-2009-AG-DGFFS-DGEFFS, 0377-2010-AG-DGFFS-DGEFFS). They thank T. Valqui, E. Bautista, and CORBIDI for assistance in the field. They thank Anny Bang and Elin Ellebæk Petersen for lab assistance in Aarhus, Kathy Williams for lab assistance in Lincoln, Matt Jones for lab assistance in Laramie, and Federico Hoffmann for bioinformatics help. They also thank P. Benham, N. Sly, and two anonymous reviewers for helpful comments on the manuscript. This work was funded by the University of Illinois Department of Animal Biology, University of Wyoming Department of Zoology and Physiology, the Faculty of Science and Technology, Aarhus University, and grants from the Danish Council for Independent Research, Natural Sciences (grant nr. 10-084-565), National Institute of Health/National Heart, Lung, and Blood Institute (R01 HL087216 and HL087216-S1) and the National Science Foundation (IOS-0949931, DEB-1146491).

References

Abramoff MD, Magelhaes PJ, Ram SJ. 2004. Image processing with Image. *J. Biophotonics Int.* 11:36–42.

Arnone A, Perutz MF. 1974. Structure of inositol hexaphosphate-human deoxyhaemoglobin complex. *Nature* 249:34–36.

Balakarishinan CN, Mukai M, Gosner RA, Wingfield JC, London SE, Tuttle EM, Clayton DF. 2014. Brain transcriptome sequencing and assembly of the songbird model systems for the study of social behavior. *PeerJ.* 2:e396.

Downloaded from http://mbe.oxfordjournals.org/ at University of Nebraska-Lincoln Libraries on August 29, 2014

Barrett RDH, Hoekstra HE. 2011. Molecular spandrels: tests of adaptation at the genetic level. *Nat Rev Genet.* 12:760–780.

Bencowitz H, Wagner P, West J. 1982. Effect of change in P_{50} on exercise tolerance at high altitude: a theoretical study. *J Appl Physiol.* 53: 1487–1495.

Berry R. 1978. Genetic variation in wild house mice: where natural selection and history meet. *Am Sci.* 66:52–60.

Castro G, Carey C, Whittembry J, Monge C. 1985. Comparative responses of sea level and montane rufous collared sparrows, *Zonotrichia capensis*, to hypoxia and cold. *Comp Biochem Physiol A Mol Integr Physiol.* 82:847–850.

Castro G, Wunder BA. 1990. Cold adaptations in the rufous-collared sparrow *Zonotrichia capensis*. *Comp Biochem Physiol A Mol Integr Physiol.* 98:101–102.

Castro G. 1983. Un estudio sobre las adaptaciones a la Altitud en el Gorrion Andino (*Zonotrichia capensis*). *Primer Symp Ornitol Neotrop.* 1:27–32.

Catchen J, Hohenlohe PA, Bassham S, Amores A, Cresko WA. 2013. Stacks: an analysis tool set for population genomics. *Mol Ecol.* 22: 3124–3140.

Catchen J, Bassham S, Wilson T, Currey M, O'Brien C, Yeates Q, Cresko WA. 2013. The population structure and recent colonization history of Oregon threespine stickleback determined using restriction-site associated DNA sequencing. *Mol Ecol.* 22:2864–2883.

Cheviron ZA, Whitehead A, Brumfield RT. 2008. Transcriptomic variation and plasticity in rufous-collared sparrows (*Zonotrichia capensis*) along an elevational gradient. *Mol Ecol.* 17:4556–4569.

Cheviron ZA, Brumfield RT. 2009. Migration-selection balance and local adaptation of mitochondrial haplotypes in rufous-collared sparrows along an elevational gradient. *Evolution* 63:1593–1605.

Cheviron ZA, Bachman GC, Storz JF. 2013. Contributions of phenotypic plasticity to population differences in thermogenic performance between highland and lowland deer mice. *J Exp Biol.* 216:1160–1166.

Cheviron ZA, Connaty AD, McClelland GB, Storz JF. 2014. Functional genomics of hypoxic cold-stress in high-altitude deer mice: transcriptomic plasticity and thermogenic performance. *Evolution* 68: 48–62.

Dalziel AC, Rogers SM, Schulte PM. 2009. Linking genotypes to phenotypes and fitness: how mechanistic biology can inform molecular ecology. *Mol Ecol.* 18:4997–5017.

Damsgaard C, Storz JF, Hoffmann FG, Fago A. 2013. Hemoglobin isoform differentiation and allosteric regulation of oxygen binding in the turtle, *Trachemys scripta*. *Am J Physiol Regul Integr Comp Physiol.* 305:R961–R967.

Dean AM, Thornton JW. 2007. Mechanistic approaches to the study of evolution: the functional synthesis. *Nat Rev Genet.* 8: 675–688.

Edwards AWF. 1972. Likelihood: an account of the statistical concept of likelihood and its application to scientific inference. Cambridge: Cambridge University Press.

Excoffier L, Laval G, Schneider S. 2005. Arlequin ver. 3.0: an integrated software package for population genetic data analysis. *Evol Bioinform Online.* 1:47–50.

Fermi G, Perutz MF. 1981. Atlas of molecular structures in biology. 2. Haemoglobin and myoglobin. Oxford: Clarendon Press.

Giustarini D, Dalle-Donne I, Cavarra E, Fineschi S, Lungarella G, Milzani A, Rossi R. 2006. Metabolism of oxidants by blood from different mouse strains. *Biochem Pharmacol.* 71:1753–1764.

Grispo MT, Natarajan C, Projecto-Garcia J, Moriyama H, Weber RE, Storz JF. 2012. Gene duplication and the evolution of hemoglobin isoform differentiation in birds. *J Biol Chem.* 287:37647–37658.

Harms MJ, Thornton JW. 2013. Evolutionary biochemistry: revealing the historical and physical causes of protein function. *Nat Rev Genet.* 14: 559–571.

Hattori Y, Ohba Y, Suda T, Miura Y, Yoshinaka H, Miyaji T. 1985. Hemoglobin GuiZhou in Japan. *Hemoglobin* 9:187–192.

Hiebl I, Weber RE, Schneeganss D, Kosters J, Braunitzer G. 1989. Structural adaptations in the major and minor hemoglobin components of adult Ruppell's griffon (*Gyps ruepellii*, Aegypiinae): a new molecular pattern for hypoxia tolerance. *Biol Chem Hoppe Seyler.* 369:217–232.

Hill WG, Weir B. 1988. Variances and covariances of squared linkage disequilibria in finite populations. *Theor Popul Biol.* 33:54–78.

Hempe JM, Ory-Ascani J, Hsia D. 2007. Genetic variation in mouse beta globin cysteine content modifies glutathione metabolism: implications for the use of mouse models. *Exp Biol Med.* 232:437–444.

Hey J, Neilsen R. 2007. Integration within the Felsenstein equation for improved Markov chain Monte Carlo methods in population genetics. *Proc Natl Acad Sci U S A.* 104:2785–2790.

Hoffmann FG, Storz JF. 2007. The α^D-globin gene originated via duplication of an embryonic α-like globin gene in the ancestor of tetrapod vertebrates. *Mol Biol Evol.* 24:1982–1990.

Hoffmann FG, Storz JF, Gorr TA, Opazo JC. 2010. Lineage-specific patterns of functional diversification in the α- and β-globin gene families of tetrapod vertebrates. *Mol Biol Evol.* 27:1126–1138.

Hoffman FG, Opazo JC, Storz JF. 2011. Differential loss and retention of cytoglobin, myoglobin, and globin E during the radiation of vertebrates. *Genome Biol Evol.* 3:588–600.

Hoffmann FG, Opazo JC, Storz JF. 2012. Whole-genome duplications spurred the functional diversification of the globin gene superfamily in vertebrates. *Mol Biol Evol.* 29:303–312.

Hudson RR. 1987. Estimating the recombination parameter of a finite population model without selection. *Genet Res.* 50:245–250.

Hudson RR, Kaplan NL. 1995. Deleterious background selection with recombination. *Genetics* 141:1605–1617.

Imai K. 1982. Allosteric effects in haemoglobin. Cambridge: Cambridge University Press.

Jessen TH, Weber RE, Fermi G, Tame J, Braunitzer G. 1991. Adaptation of bird hemoglobins to high-altitudes—demonstration of molecular mechanism by protein engineering. *Proc Natl Acad Sci U S A.* 88: 6519–6522.

Kelly JK. 1997. A test of neutrality based on interlocus associations. *Genetics* 146:1197–1206.

Langmead B, Salzberg SL. 2012. Fast-gapped read alignment with Bowtie 2. *Nat Methods* 9:357–359.

Librado P, Rozas J. 2009. DnaSP v5: a software for comprehensive analysis of DNA polymorphism data. *Bioinformatics* 25:1451–1452.

Mairbaurl H, Weber RE. 2012. Oxygen transport by hemoglobin. *Compr Physiol.* 2:1463–1489.

Marden JH. 2013. Nature's inordinate fondness for metabolic enzymes: why metabolic enzyme loci are so frequently targets of selection. *Mol Ecol.* 22:5743–5764.

McCracken KG, Barger CP, Bulgarella M, Johnson KP, Sonsthagen SA, Trucco J, Valqui TH, Wilson RE, Winker K, Sorenson MD. 2009. Parallel evolution in the major haemoglobin genes of eight species of Andean waterfowl. *Mol Ecol.* 18:3992–4005.

Monge C, León-Velarde F. 1991. Physiological adaptation to high altitude: oxygen transport in mammals and birds. *Physiol Rev.* 71: 1135–1172.

Natarajan C, Jiang XB, Fago A, Weber RE, Moriyama H, Storz JF. 2011. Expression and purification of recombinant hemoglobin in *Escherichia coli*. *PLoS One* 6:e20176.

Natarajan C, Inoguchi N, Weber RE, Fago A, Moriyama H, Storz JF. 2013. Epistasis among adaptive mutations in deer mouse hemoglobin. *Science* 340:1324–1327.

Nikinmaa M. 2001. Haemoglobin function in vertebrates: evolutionary changes in cellular regulation in hypoxia. *Respir Physiol.* 128:317–329.

Parchman TL, Gompert Z, Benkman CW, Schilkey FD, Mudge J, Buerkle CA. 2012. Genome wide association mapping of an adaptive trait in lodgepole pine. *Mol Ecol.* 21:2836–2838.

Petschow D, Wurdinger I, Baumann R, Duhm J, Braunitzer G, Bauer C. 1977. Causes of high blood O_2 affinity of animals living at high altitude. *J Appl Physiol.* 42:139–143.

Poelwijk FJ, Kivet DJ, Weinreich DM, Tans SJ. 2007. Empirical fitness landscapes reveal accessible paths. *Nature* 445:383–386.

Porter AH, Wenger RH, Geiger H, Scholl A, Shapiro AM. 1997. The *Pontia daplidice-edusa* hybrid zone in northwestern Italy. *Evolution* 51: 1561–1573.

Downloaded from http://mbe.oxfordjournals.org/ at University of Nebraka-Lincoln Libraries on August 29, 2014

Projecto-Garcia J, Natarajan C, Moriyama H, Weber RE, Fago A, Cheviron ZA, Dudley R, McGuire JA, Witt CC, Storz JF. 2013. Repeated elevational transitions in hemoglobin function during the evolution of Andean hummingbirds. *Proc Natl Acad Sci U S A.* 110:20669–20674.

R Core Team. 2012. R: a language and environment for statistical computing. Vienna (Austria): R Foundation for Statistical Computing. Available from: http://www.R-project.org/.

Revsbech I, Tufts DM, Projecto-Garcia J, Moriyama H, Weber RE, Storz JF, Fago A. 2013. Hemoglobin function and allosteric regulation in semi-fossorial rodents (family Sciuridae) with different altitudinal ranges. *J Exp Biol.* 216:4264–4271.

Riccio A, Tamburrini M, Giardina B, di Prisco G. 2001. Molecular dynamics analysis of a second phosphate site in the hemoglobins of the seabird, south polar skua. Is there a site-site migratory mechanism along the central cavity? *Biophys J.* 81:1938–1946.

Runck AM, Moriyama H, Storz JF. 2009. Evolution of duplicated β-globin genes and the structural basis of hemoglobin isoform differentiation in *Mus*. *Mol Biol Evol.* 26:2521–2532.

Runck AM, Weber RE, Fago A, Storz JF. 2010. Evolutionary and functional properties of a two-locus β-globin polymorphism in Indian house mice. *Genetics* 184:1121–1131.

Samaja M, Crespi T, Guazzi M, Vandegriff KD. 2003. Oxygen transport in blood at high altitude: role of the hemoglobin-oxygen affinity and impact of the phenomena related to hemoglobin allosterism and red cell function. *Eur J Biochem.* 90:351–359.

Schulenberg TS, Stotz DF, Lane DF, O'Neill JP, Parker TP. 2007. Birds of Peru. Princeton (NJ): Princeton University Press.

Scott GR, Milsom WK. 2006. Flying high: a theoretical analysis of the factors limiting exercise performance in birds at altitude. *Respir Physiol Neurobiol.* 154:284–301.

Scott GR. 2011. Elevated performance: the unique physiology of birds that fly at high altitudes. *J Exp Biol.* 214:2455–2462.

Stephens M, Donnelly P. 2003. A comparison of Bayesian methods for haplotype reconstruction from population genotype data. *Am J Hum Genet.* 73:1162–1169.

Stephens M, Smith N, Donnelly P. 2001. A new method for haplotype reconstruction from population data. *Am J Hum Genet.* 68:978–989.

Storz JF. 2007. Hemoglobin function and physiological adaptation to hypoxia in high-altitude mammals. *J Mammal.* 88:24–31.

Storz JF, Moriyama H. 2008. Mechanisms of hemoglobin adaptation to high-altitude hypoxia. *High Alt Med Biol.* 9:148–157.

Storz JF, Wheat CW. 2010. Integrating evolutionary and functional approaches for inferring adaptation at specific loci. *Evolution* 64:2489–2509.

Storz JF, Zera AJ. 2011. Experimental approaches to evaluate the contributions of candidate protein-coding mutations to phenotypic evolution. In: Orgogozo V, Rockman MV, editors. Molecular methods in evolutionary genetics. New York: Springer. p. 377–396.

Storz JF, Baze M, Waite JL, Hoffmann FG, Opazo JC, Hayes JP. 2007. Complex signatures of selection and gene conversion in the duplicated globin genes of house mice. *Genetics* 177:481–500.

Storz JF, Runck AM, Sabatino SJ, Kelly JK, Ferrand N, Moriyama H, Weber RE, Fago A. 2009. Evolutionary and functional insights into the mechanism underlying high-altitude adaptation of deer mouse hemoglobin. *Proc Natl Acad Sci U S A.* 106:14450–14455.

Storz JF, Runck AM, Moriyama H, Weber RE, Fago A. 2010. Genetic differences in hemoglobin function between highland and lowland deer mice. *J Exp Biol.* 213:2565–2574.

Storz JF, Scott GR, Cheviron ZA. 2010. Phenotypic plasticity and genetic adaptation to high-altitude hypoxia in vertebrates. *J Exp Biol.* 213:4125–4136.

Storz JF, Hoffman FG, Opazo JC, Sanger TJ, Moriyama H. 2011. Developmental regulation of hemoglobin synthesis in the green anole lizard, *Anolis carolinensis*. *J Exp Biol.* 214:575–581.

Storz JF, Weber RE, Fago A. 2012. Oxygenation properties and oxidation rates of mouse hemoglobins that differ in reactive cysteine content. *Comp Biochem Physiol A Mol Integr Physiol.* 161:265–270.

Szelenyi JG, Horanyi M, Foldi J, Hudacsek J, Istvan L, Hollan SR. 1980. A new hemoglobin variant in Hungary: Hb Savaria—α49(CE7)Ser→Arg. *Hemoglobin* 4:27–38.

Tajima F. 1989. Statistical method of testing the neutral mutation hypothesis by DNA polymorphism. *Genetics* 123:585–595.

Tamburrini M, Riccio A, Romano M, Giardina B, di Prisco G. 2000. Structural and functional analysis of the two haemoglobins of the Antarctic seabird *Catharacta maccormicki*. *Eur J Biochem.* 267:6089–6098.

Tufts DM, Revsbech I, Cheviron ZA, Weber RE, Fago A, Storz JF. 2013. Phenotypic plasticity in blood-oxygen transport in highland and lowland deer mice. *J Exp Biol.* 216:1167–1173.

Turek Z, Kreuzer F. 1976. Effect of a shift of the oxygen dissociation curve on myocardial oxygenation at hypoxia. *Adv Exp Med Biol.* 75:657–662.

Turek Z, Kreuzer F, Hoofd L. 1973. Advantage or disadvantage of a decrease of blood oxygen affinity for tissue oxygen supply at hypoxia. A theoretical study comparing man and rat. *Pflügers Arch.* 342:185–187.

Turek Z, Kreuzer F, Ringnalda BEM. 1978. Blood gases at several levels of oxygenation in rats with a left-shifted blood oxygen dissociation curve. *Pflügers Arch.* 376:7–13.

Turek Z, Kreuzer F, Turek-Maischeider M, Ringnalda BEM. 1978. Blood oxygen content, cardiac output, and flow to organs at several levels of oxygenation in rats with a left-shifted blood oxygen dissociation curve. *Pflügers Arch.* 376:201–207.

Weber RE. 1981. Cationic control of oxygen affinity in lugworm erythocruorin. *Nature* 292:386–387.

Weber RE. 1992. Use of ionic and zwitterionic (Tris/BisTris and HEPES) buffers in studies on hemoglobin function. *J Appl Physiol.* 72:1611–1615.

Weber RE. 2007. High-altitude adaptations in vertebrate hemoglobins. *Respir Physiol Neurobiol.* 158:132–142.

Weber RE, Fago A. 2004. Functional adaptation and its molecular basis in vertebrate hemoglobins, neuroglobins and cytoglobins. *Respir Physiol Neurobiol.* 144:141–159.

Weber RE, Hiebl I, Braunitzer G. 1988. High altitude and hemoglobin function in the vultures *Gyps rueppellii* and *Aegypius monachus*. *Biol Chem Hoppe Seyler.* 369:233–240.

Weber RE, Jessen TH, Malte H, Tame J. 1993. Mutant hemoglobins (α^{119}-Ala and β^{55}-Ser)—functions related to high-altitude respiration in geese. *J Appl Physiol.* 75:2646–2655.

Weber RE, Voelter W, Fago A, Echner H, Campanella E, Low PS. 2004. Modulation of red cell glycolysis: interactions between vertebrate hemoglobins and cytoplasmic domains of band 3 red cell membrane proteins. *Am J Physiol Regul Integr Comp Physiol.* 287:R454–R464.

Weber RE, Ostojic H, Fago A, Dewilde S, Van Hauwaert ML, Moens L, Monge C. 2002. Novel mechanism for high-altitude adaptation in hemoglobin of the Andean frog *Telmatobius peruvianus*. *Am J Physiol Regul Integr Comp Physiol.* 283:R1052–R1060.

Weinreich DM, Watson RA, Chao L. 2005. Sign epistasis and constraint on evolutionary trajectories. *Evolution* 59:1165–1174.

Willford DC, Hill EP, Moores WY. 1982. Theoretical analysis of optimal P_{50}. *J Appl Physiol.* 52:1043–1048.

Yokoyama S. 2012. Synthesis of experimental biology and evolutionary biology: an example from the world of vision. *Bioscience* 62:939–948.

Downloaded from http://mbe.oxfordjournals.org/ at University of Nebraska-Lincoln Libraries on August 29, 2014
