# Supplementary material for: Spatially variable coevolution between a haemosporidian parasite and the MHC of a widely distributed passerine
Source: Ecol Evol. 2015 Feb 6;5(5):1045–60. doi: 10.1002/ece3.1391 (PMC4364819; doi:10.1002/ece3.1391)
Supplement: Supplementary file 2 [file ece30005-1045-sd2.pdf]

Immunogenetics
DOI 10.1007/s00251-014-0800-7

ORIGINAL PAPER

Variation in positively selected major histocompatibility complex class I loci in rufous-collared sparrows (*Zonotrichia capensis*)

Matthew R. Jones · Zachary A. Cheviron · Matthew D. Carling

Received: 18 April 2014 / Accepted: 25 August 2014
© Springer-Verlag Berlin Heidelberg 2014

Abstract The major histocompatibility complex (MHC) is a highly variable family of genes involved in parasite recognition and the initiation of adaptive immune system responses. Variation in MHC loci is maintained primarily through parasite-mediated selection or disassortative mate choice. To characterize MHC diversity of rufous-collared sparrows (*Zonotrichia capensis*), an abundant South American passerine, we examined allelic and nucleotide variation in MHC class I exon 3 using pyrosequencing. Exon 3 comprises a substantial portion of the peptide-binding region (PBR) of class I MHC and thus plays an important role in intracellular pathogen defense. We identified 98 putatively functional alleles that produce 56 unique protein sequences across at least 6 paralogous loci. Allelic diversity per individual and exon-wide nucleotide diversity were relatively low; however, we found specific amino acid positions with high nucleotide diversity and signatures of positive selection (elevated d_N/d_S) that may correspond to the PBR. Based on the variation in physicochemical properties of amino acids at these "positively selected sites," we identified ten functional MHC supertypes. Spatial variation in nucleotide diversity and the number of MHC alleles, proteins, and supertypes per individual suggests that environmental heterogeneity may affect patterns of MHC diversity. Furthermore, populations with high MHC diversity have higher prevalence of avian malaria, consistent with parasite-mediated selection on MHC. Together, these results provide a framework for subsequent investigations of selective agents acting on MHC in *Z. capensis*.

Keywords Genetic variation · Elevational gradient · Major histocompatibility complex · Natural selection · Parasites · *Zonotrichia capensis*

Electronic supplementary material The online version of this article (doi:10.1007/s00251-014-0800-7) contains supplementary material, which is available to authorized users.

M. R. Jones (✉) · M. D. Carling
Department of Zoology and Physiology, Berry Biodiversity Conservation Center, University of Wyoming, 1000 E. University Ave., Dept. 4304, Laramie, WY 82071, USA
e-mail: jonesmr9@gmail.com

Z. A. Cheviron
Department of Animal Biology, School of Integrative Biology, University of Illinois Urbana-Champaign, 505 South Goodwin Ave., Urbana, IL 61801, USA

Present Address:
M. R. Jones
Division of Biological Sciences, University of Montana, 32 Campus Dr. HS104, Missoula, MT 59812, USA

Background

The major histocompatibility complex (MHC) has received considerable attention from the fields of ecology, conservation, and evolutionary biology in recent years because of its relevance to disease resistance and its high level of genetic variability within and between species (Spurgin and Richardson 2010). The MHC is an essential component of the vertebrate adaptive immune system that encodes intracellular (MHC class I or MHC-I) and extracellular (MHC class II or MHC-II) peptide-binding proteins. MHC peptide-binding regions (PBRs) bind a specific range of foreign pathogen-derived peptides or self-derived peptides and present them to cytotoxic T cells to initiate the adaptive immune system responses (Matsumura et al. 1992). The binding specificity between MHC proteins and pathogen peptides provides an important mechanism of disease resistance in hosts (Hammer et al. 1995; Wucherpfennig et al. 1995). For example, MHC genotypes in many species are associated with resistance or susceptibility to diseases and parasites such as malaria (Hill 1998; Westerdahl et al. 2005; Bonneaud et al. 2006; Westerdahl et al. 2012), Rous sarcoma (LePage et al. 2000;

Published online: 04 September 2014

Wallny et al. 2006), and Marek's disease (Cole 1968; Wakenell et al. 1996; Wang et al. 2014). Understanding population level MHC diversity and variation may therefore enhance our knowledge of disease ecology and host-parasite evolutionary dynamics.

MHC is among the most variable gene families in vertebrates and has contributed to our understanding of evolutionary mechanisms maintaining genetic variation in populations (Spurgin and Richardson 2010). For example, the human MHC (or human leukocyte antigen (HLA)) gene family comprises over 400 loci distributed across approximately 7.6 Mb of the human genome (Horton et al. 2004). Since estimates of HLA mutation rates appear relatively low for primate genes, disease-related selection pressures or mate choice patterns likely drive the levels of standing MHC variation (Klein et al. 1993; Edwards and Hedrick 1998). Several nonexclusive mechanisms of parasite-mediated selection have been proposed to explain high MHC diversity in vertebrates, including heterozygote advantage, negative frequency-dependent selection, and fluctuating selection (Spurgin and Richardson 2010). Under the heterozygote advantage hypothesis, individuals with a high diversity of MHC alleles are able to bind a wider range of pathogen-derived peptides and are less likely to harbor infections from a wide array of parasites (Hughes and Nei 1988; Penn et al. 2002; Wegner et al. 2003; Kloch et al. 2010). The negative frequency-dependent selection hypothesis suggests that rare MHC alleles are more likely to confer resistance to specific parasites than more common alleles (Takahata and Nei 1990). Finally, the fluctuating selection hypothesis proposes that MHC variation arises as a result of spatial or temporal heterogeneity in the strength or specificity of directional parasite-mediated selection on MHC (Hedrick 2002; Loiseau et al. 2010). MHC diversity may also be affected by other historical factors including population bottlenecks followed by genetic drift (Babik et al. 2009a; Sutton et al. 2011; Sutton et al. 2013), gene flow (Hansen et al. 2007; Nadachowska-Brzyska et al. 2012), and sexual selection (Bernatchez and Landry 2003). MHC genotypes are correlated with the quality of sexually selected traits in several species, suggesting that selection on MHC may follow the "good genes" hypothesis of sexual selection to enhance parasite resistance of offspring (von-Schantz et al. 1996; Ditchkoff et al. 2001; Dunn et al. 2013). Individuals may choose mates with high MHC variation or dissimilar MHC genotypes (disassortative mating) to maximize allelic diversity in offspring or to avoid inbreeding (Reusch et al. 2001).

Across families of birds, the composition and variability of MHC genes are highly diverse. A relatively simplistic MHC is believed to be the ancestral MHC state in birds. For example, chickens possess a "minimal essential MHC," named for its highly compact and minimalist gene composition, and are basal in avian phylogenies (Kaufman et al. 1999; Hackett et al. 2008). Chicken MHC consists of only 46 tightly linked genes across a 242-kb region on chromosome 16 (Kaufman et al. 1999; Shiina et al. 2007; Delany et al. 2009). Other nonpasserines, for example parrots (Hughes et al. 2008) and some birds of prey (Alcaide et al. 2007), also possess a simplistic, minimal essential MHC, while some basal bird species (e.g., *Coturnix japonica*, *Apteryx owenii*) possess a relatively more complex MHC that is composed of multiple functional paralogs and pseudogenes, complicating inferences of the ancestral MHC state in birds (Shiina et al. 2004; Miller et al. 2011). The simplistic nature of the minimal essential MHC and reduced recombination at these loci is expected to enhance long-term coevolution between genes and affect the evolution and functionality of coadapted gene complexes (Kaufman et al. 1999).

By contrast, passerines have a more complex MHC composed of multiple functional paralogs and pseudogenes (Westerdahl 2007; Sepil et al. 2012). The Zebra Finch MHC spans a much longer distance (~739-kb region) across at least two chromosomes (Balakrishnan et al. 2010; Ekblom et al. 2011). Like other complex gene families, variation in MHC gene copy number is believed to arise through repeated gene duplication events with subsequent selection on or degradation of paralogous loci.

Characterizing genetic variation at the MHC is an important task for understanding disease resistance as well as elucidating evolutionary mechanisms responsible for maintaining genetic variation in populations. Accurate genotyping of MHC is a challenge (Westerdahl 2007; Sepil et al. 2012), and recently, next-generation 454 pyrosequencing has been applied to characterize the MHC in fish (Ellison et al. 2012; Lamaze et al. 2014), nonavian reptile (Stiebens et al. 2013), bird (Zagalska-Neubauer et al. 2010; Spurgin et al. 2011; Sepil et al. 2012; Strandh et al. 2012; Dunn et al. 2013), and mammal species (Babik et al. 2009b; Wiseman et al. 2009; Kloch et al. 2010; Babik et al. 2012). Pyrosequencing of MHC amplicons provides deep coverage of alleles across multiple loci and is a powerful method for detecting rare variants in multilocus systems (Promerová et al. 2012). This method comes at the cost of not being able to assign alleles to specific loci since all MHC paralogs are sequenced in parallel. However, alleles across multiple loci can be functionally grouped into MHC "supertypes," which share physicochemical or peptide-binding characteristics (Doytchinova and Flower 2005; Ellison et al. 2012; Sepil et al. 2012). MHC supertypes may more accurately reflect the units of selection on MHC since different MHC alleles may not differ at functionally important sites. Thus, identification of MHC supertypes can facilitate inferences of selection on MHC diversity and its relationship to disease (Doytchinova and Flower 2005; Sepil et al. 2012).

We investigated variation at MHC-I exon 3 of *Zonotrichia capensis* (rufous-collared sparrow), a widely distributed (from 0 to roughly 5,000 m a.s.l.) and abundant Neotropical

passerine using 454 pyrosequencing. MHC-I molecules are found on nearly all somatic cells of birds and bind peptides derived from intracellular pathogens, such as malaria and viruses (Bonneaud et al. 2006; Wallny et al. 2006; Westerdahl 2007). Avian MHC-I molecules are heterodimers composed of an α chain and β_2-macroglobulin (Westerdahl et al. 1999). The majority of polymorphism at avian class I MHC resides in exon 2 and, in particular, exon 3 (α_1 and α_2 domains), which encode the PBR (Bjorkman et al. 1987; Alcaide et al. 2009; Alcaide et al. 2013). We focused our efforts on MHC-I exon 3 because it is generally more polymorphic than exon 2 and some evidence suggests that it plays a larger role in disease resistance (Sepil et al. 2012; Wang et al. 2014). Our goals were to (1) characterize allelic and nucleotide MHC variation within *Z. capensis* distributed along an elevational gradient on the western slope of the Peruvian Andes, (2) investigate signatures of selection on MHC, and (3) identify functionally related MHC alleles (MHC supertypes) based on amino acid variation at positively selected sites (PSSs).

Methods

Sample collection

We obtained pectoral muscle tissue samples from 184 *Z. capensis* specimens collected along three elevational transects (T1–T3) on the western slope of the Peruvian Andes from 2004 to 2007 (Fig. 1). Individuals were either shot or collected in mist nests and euthanized using methods approved by the Institutional Animal Care and Use Committee

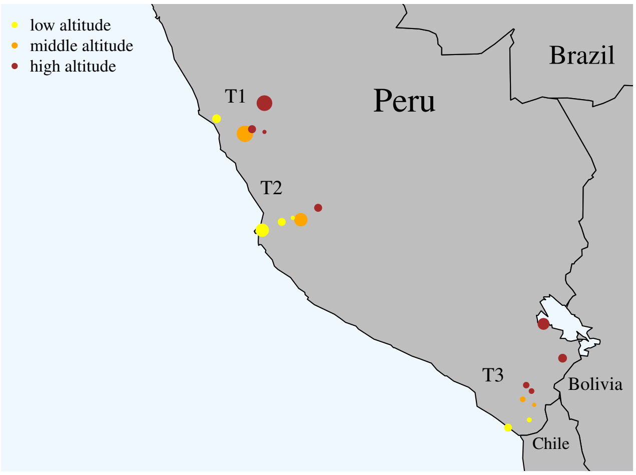

Fig. 1 Sampling sites for the three elevational transects (T1–T3). The size of each *dot*, representing a sampling locality, scales with the number of individuals collected at the site (range 4–25 individuals per site). Sites classified as low (0–1,500 m), middle (1,501–3,100 m), and high elevation (3,101–4,150 m) are represented as *yellow*, *orange*, and *brown*, respectively (color figure online)

at Louisiana State University (IACUC protocol number LSU #06-124). Total genomic DNA was extracted from flash-frozen pectoral muscle using DNeasy tissue extraction kits (Qiagen, Valencia, CA).

Although little is known about local movement patterns among *Z. capensis* populations, on the western slope of the Peruvian Andes, *Z. capensis* populations are nonmigratory and abundant (Schulenberg et al. 2007; Cheviron and Brumfield 2009). At low elevations, *Z. capensis* is patchily distributed and restricted to desert oases and irrigated farmlands (Schulenberg et al. 2007; Cheviron and Brumfield 2009). Along elevational transects, mean elevation gain was ~3,900 m over a mean linear distance of ~161 km. We performed analyses on *Z. capensis* individuals classified by elevational zones across all transects (low elevation 0–1,500 m, middle elevation 1,501–3,100 m, high elevation 3,101–4,150 m) and by transect (T1, T2, and T3; Fig. 1), used as a proxy for latitude.

MHC library preparation and variant calling

Initially, we tested MHC primers A21 and A23 (Westerdahl et al. 1999) for their ability to amplify MHC-I exon 3 in *Z. capensis*, but these primers failed to amplify products within the expected size distribution. We then chose forward primer GCA21M (5′-CGTACAGCGGCTTGTTGGCTGTGA-3′) and reverse primer fA23M (5′-GCGCTCCAGCTCCTTCTGCCCATA-3′), which were designed to amplify MHC-I exon 3 in house sparrows (*Passer domesticus*; Loiseau et al. 2010). This primer pair amplified a 214-bp fragment of exon 3, which falls within the size distribution of other passerine species (Bonneaud et al. 2004; Loiseau et al. 2010; Schut et al. 2011; Sepil et al. 2012). Pyrosequencing was performed with two titanium fusion primers (forward 5′-CGTATCGCCTCCCTCGCGCCATCAG-3′; reverse 5′-CTATGCGCCTTGCCAGCCCGCTCAG-3′) ligated to a 10-bp multiplex identifier (MID) tag (Online Resource 1) and the forward or reverse MHC primer (GCA21M and fA23M) with phosphorothiolate bonds, resulting in a 59-bp primer sequence.

To maximize the concentration of MHC amplicons for sequencing, we performed two rounds of PCR amplification. First, we prepared a 10-μl reaction with 6.15 μl dH_2O, 1.0 μl (~20 ng) genomic DNA, 1.0 μl $MgCl_2$ (2.5 mM), 1.0 μl 10× PCR buffer, 0.25 μl dNTPs (10 mM for each dNTP), 0.25 μl of each MHC primer (10 mM), and 0.1 μl AmpliTaq (5 U/μl; Applied Biosystems, Foster City, CA). Following the first PCR, we prepared a 10-μl reaction with 6.35 μl dH_2O, 1.0 μl of template from initial PCR, 1.0 μl $MgCl_2$ (2.5 mM), 1.0 μl 10× PCR buffer, 0.25 μl dNTPs (10 mM for each dNTP), 0.15 μl of each pyrosequencing primer (10 mM), and 0.1 μl AmpliTaq (5 U/μl; Applied Biosystems, Foster City, CA). The thermocycler profile consisted of an

initialization step at 95 °C for 3 min, followed by 10 cycles (first PCR) or 35 cycles (second PCR) of 94 °C denaturation for 30 s, 64 °C annealing for 30 s, and 72 °C extension for 30 s, with a final elongation at 72 °C for 10 min. Amplicon libraries were purified using the AMPure PCR purification kit following the manufacturer's directions (Agencourt, Beverly, MA). We assessed individual amplicon concentration using a Qubit 2.0 Fluorometer and pooled individual amplicons into equimolar quantities for sequencing. The pooled library was sent to the Genome Sequencing & Analysis Core Resource at Duke University and sequenced on one half of a picotiter plate on a Roche 454 GS FLX+platform.

Reads were aligned and sorted by individual using unique MID tags after removing reads with ambiguous base pair assignments or incomplete primer or tag sequences using *jMHC* (Stuglik et al. 2011). Unique variants were then identified as well as the number of reads of each variant per individual (*jMHC*; Stuglik et al. 2011). We used a five-step variant validation procedure to attempt to exclude sequencing error artifacts and pseudo-alleles from the MHC dataset. We focused our study on variation at putatively functional MHC alleles because of their potential relevance in disease resistance. Here, we define "putatively functional" alleles as those with an open reading frame (Galan et al. 2010; Zagalska-Neubauer et al. 2010; Sepil et al. 2012). First, we removed all reads that were not 214 bp in length or that contained stop codons within the sequence. We focused on 214-bp reads because the majority of our reads (61.4 %) were this length and we wanted to remove pseudoalleles, which are often characterized by indels (Ophir and Graur 1997). However, we cannot be certain that we removed a fraction of functional alleles not 214 bp in length or that we amplified 214-bp nonfunctional alleles. Second, we removed individuals that had fewer than 200 total reads, which are unlikely to have been accurately genotyped (Galan et al. 2010; Sepil et al. 2012). The probability of accurately genotyping all alleles in an individual with more than 200 reads, a ploidy level of 12 (6 MHC-I loci, see "Results"), and a minimum number of reads per allele of 2 is very high ($P>0.999$; Galan et al. 2010). Hence, we have chosen a very conservative cutoff value for individual genotyping. Third, sequences with a maximum per allele frequency (MPAF) less than 0.01 were removed from the dataset (Zagalska-Neubauer et al. 2010; Sepil et al. 2012). These are alleles that comprised less than 1 % of the total number of reads within an individual and likely occur due to sequencing error. Fourth, we removed all sequences that occurred fewer than five times in the entire dataset and only once within an individual. Assuming a mean substitution error rate of 1.07 % (Gilles et al. 2011), the probability of observing the same sequencing error five times in our dataset is very low ($P<10^{-9}$; Galan et al. 2010). Finally, we removed variants that appeared in only a single individual in the dataset. These are variants that may have reached a high frequency in a single individual as a result of PCR duplication of single nucleotide misincorporation errors or chimeras (Sepil et al. 2012). With the final dataset, we tested for correlation between individual coverage and number of alleles using ordinary least squares linear regression in R (R Development Core Team 2013). To examine differences in the number of MHC alleles per individual by transect and elevational zone, we performed a two-way ANOVA with transect and elevational zone as fixed factors in R (R Development Core Team 2013). For pairwise comparisons of individual allelic diversity between transects and elevational zones, we used a Student's *t* test.

MHC phylogeny construction

We inferred a phylogeny of putatively functional MHC alleles using MrBayes v.3.2.1 (Ronquist and Huelsenbeck 2003) with *Carpodacus erythrinus* MHC-I exon 3 (*Caer_101*; Promerová et al. 2012) as an outgroup (Fig. 2). We determined an appropriate model of nucleotide substitution using FINDMODEL (Tao et al. 2005). Likelihood scores were calculated from a Weighbor tree, and substitution models were assessed based on the Akaike Information Criterion score (Tao et al. 2005). A generalized time-reversible (GTR)+Γ was found to be the best-fit nucleotide substitution model. A GTR+Γ model uses six substitution rate parameters and four equilibrium base frequency parameters and a shape parameter (Γ) for the gamma-distributed site rate heterogeneity. To converge on the posterior probability distribution for the phylogeny, we ran 50 million Markov Chain Monte Carlo (MCMC) generations using default priors, sampling trees every 10,000 generations. We ran four independent runs with a random initial tree and four chains (one cold, three heated) and calculated a majority-rule consensus tree using the last 10 % of sampled trees from each of the four runs. MHC gene topologies are difficult to resolve due to their rapid rate of molecular evolution and duplication (Hughes and Nei 1989; Takahashi et al. 2000). To independently evaluate support for low support nodes (<75 %) on our final consensus tree, we calculated majority-rule consensus trees using the last 10 % of sampled trees for each replicate. We then checked if the major splits (those involving four or more alleles) and node support values were similar between each replicate tree and final consensus tree.

Tests of historical selection

To examine signals of historical selection on paralagous MHC-I exon 3 loci, we calculated the ratio of nonsynonymous (d_N) to synonymous mutations (d_S) in the CodeML module implemented in the software PAML (Yang 2007). Ratios of nonsynonymous to synonymous substitution rates significantly >1 are indicative of positive selection, whereas ratios significantly <1 are indicative of negative selection. Importantly,

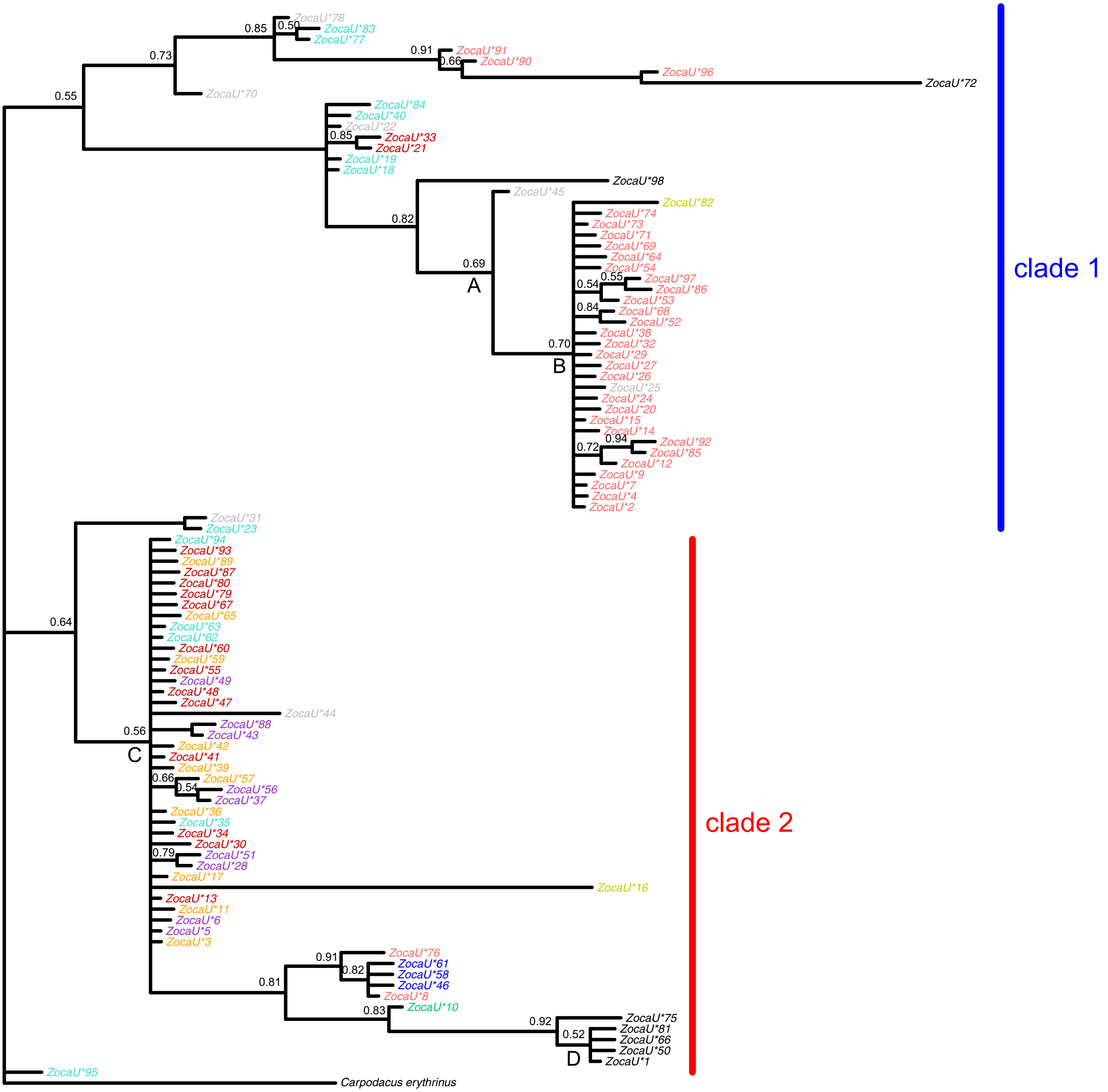

Fig. 2 MrBayes majority-rule consensus tree depicting two major clades of MHC alleles. *Colors* of allele names correspond to their supertype cluster (*black* = 1, *dark red* = 2, *green* = 3, *blue* = 4, *turquoise* = 5, *red* = 6, *gold* = 7, *gray* = 8, *purple* = 9, *orange* = 10). The *red* and *blue bars* represent the alleles within each clade (see text). Posterior support value for each node is 1.0 unless otherwise noted. Nodes A and B were collapsed in a consensus tree produced from one independent run, while nodes C and D were collapsed in a separate run (color figure online)

while selection operates at the allele level (or groups of alleles), individual sites may contribute disproportionately to the overall fitness of an allele and thus show strong signatures of positive selection. At the MHC, we expect sites corresponding to the PBR to contribute disproportionally to allele fitness and show signatures of stronger positive selection relative to other sites because they are directly involved in pathogen peptide-binding (Hughes and Nei 1988). Therefore, we used a "sites" model, which allows d_N/d_S to vary across amino acid positions. We examined two hypotheses of codon evolution: (1) a nearly neutral codon substitution model (M1), which allows sites to have $d_N/d_S \leq 1$ and (2) a positive selection codon substitution model (M2), which allows sites to have $d_N/d_S \leq 1$ or $d_N/d_S > 1$. To examine the relative support for each model, we used a likelihood ratio and an empirical Bayes procedure to identify PSSs,

which are defined as sites bearing a significantly elevated d_N/d_S ratio.

Tests of selection based on d_N/d_S are theoretically problematic for MHC datasets because they do not account for uncertainty in the tree topology, which is difficult to resolve for paralogous MHC loci. High recombination rates can lead to multiple tree topologies for population sequences, which in turn can increase false positives in a d_N/d_S outlier test (Anisimova et al. 2003; Wilson and McVean 2006). Because our sequencing strategy produced unphased MHC genotypes and an unknown number of MHC loci, we are unable to formally account for recombination in our dataset. The empirical Bayes approach that we applied here has been shown to have high power and accuracy for detecting true PSSs (M2 model) in the presence of high rates of recombination (Anisimova et al. 2003). Additionally, detecting signatures of selection based on d_N/d_S tests can be relatively robust to alternative topologies (Stager et al. 2014). The results of d_N/d_S analyses applied to within species datasets should also be interpreted cautiously since this analysis assumes fixed differences between populations rather than segregating polymorphisms within populations (Kryazhimskiy and Plotkin 2008). However, we are comparing MHC paralogs with divergences that are much older than the population level splits and that likely possess fixed differences. Furthermore, the "random-sites" model of codon evolution, similar to the sites model we used, has been demonstrated to detect signatures of strong positive selection in the PBR of human population MHC datasets (Yang and Swanson 2002).

To classify the "putative PBR" in *Z. capensis*, we aligned *Z. capensis* MHC-I exon 3 to *Gallus gallus* and assumed conservation of codon positions comprising the PBR in *G. gallus* (Fig. 3). We calculated nucleotide diversity and proportion of segregating sites across the entire exon, within the PSSs and putative PBR, and outside of the PSSs and putative PBR using *pegas* (Paradis 2010) in R (R Development Core Team 2013). Nucleotide diversity was calculated as the sum of the number of pairwise differences between alleles divided by the number of comparisons (Paradis 2010). To examine spatial differences in nucleotide diversity, we performed a one-way ANOVA with transect and elevational zone as fixed factors. We used a Student's *t* test to examine pairwise differences in nucleotide diversity across different codon classes (whole exon, PBR and PSS, and non-PBR and non-PSS), MHC clades, and spatial zones.

MHC supertypes

Physicochemical variation at the PBR and PSSs is expected to provide the phenotypic targets of natural selection on MHC alleles (Bernatchez and Landry 2003). We characterized the physicochemical properties of PSSs as proposed by Doytchinova and Flower (2005). We aligned amino acid sequences from PSSs, characterized those amino acids based on five *z*-descriptor variables—hydrophobicity (*z1*), steric bulk (*z2*), polarity (*z3*), and electronic effects (*z4* and *z5*)—and translated them into a matrix (Ellison et al. 2012; Sepil et al. 2012). To identify MHC "supertype" clusters and describe those clusters, we performed a K-means clustering algorithm and a principal components analysis using *adegenet* (Jombart 2008; Jombart et al. 2010) in R (R Development Core Team 2013). The number of supertype clusters was determined using a Bayesian Information Criterion score after retaining five principal components.

Results

Population and individual level variation

Initially, we obtained a total of 616,150 reads and 5,364 unique variants. Seven individuals with fewer than 200 reads were removed from subsequent analyses leaving a total of 177 individuals. After removing all reads with ambiguous base pair assignments, incomplete primer or tag sequences, or reads not 214 bp in length, our dataset was reduced to 360,156 reads (58.5 % of original reads). Our final dataset consisted of 98 unique MHC alleles (GenBank KF433977-KF434074), which comprise 56 unique MHC protein sequences. Mean coverage across MHC amplicons per individual was 2,035± 787 (range 203–5,072 reads). We did not find a significant correlation between the number of MHC alleles per individual and the number of reads per individual (P=0.864, R^2=0.002, Online Resource 2). Across all alleles, 31.8 % of nucleotides were polymorphic (68/214), 49.3 % of amino acid positions were polymorphic (35/71), and nucleotide diversity was 0.085±0.002 (Table 1). Mean pairwise sequence divergence among MHC alleles was 8.52 % and ranged from 0.47 to 16.36 %. The mean number of MHC alleles per individual was 4.36±1.75 corresponding to a mean of 3.56±1.42 MHC proteins per individual. The number of MHC alleles per individual varied from 1 to 11, which yields a minimum of 6 MHC-I loci. Most alleles were rare (<5 %), and only one allele (*ZocaU*1*) was found in over half of the individuals (Online Resource 3).

We identified ten MHC supertype clusters based on physicochemical variation at PSSs (Fig. 4, Online Resource 4). Individuals possessed a mean of 3.21±1.11 MHC supertypes. The first three principal components explained 92.4 % of variation in MHC supertypes (standard deviation=2.03). The first two principal components explained 83.8 % of variation and were plotted together (Fig. 4) to summarize differences among clusters. PC1 was strongly associated with variation in electronic effects (*z*4) at codon 61 (r=0.862), and

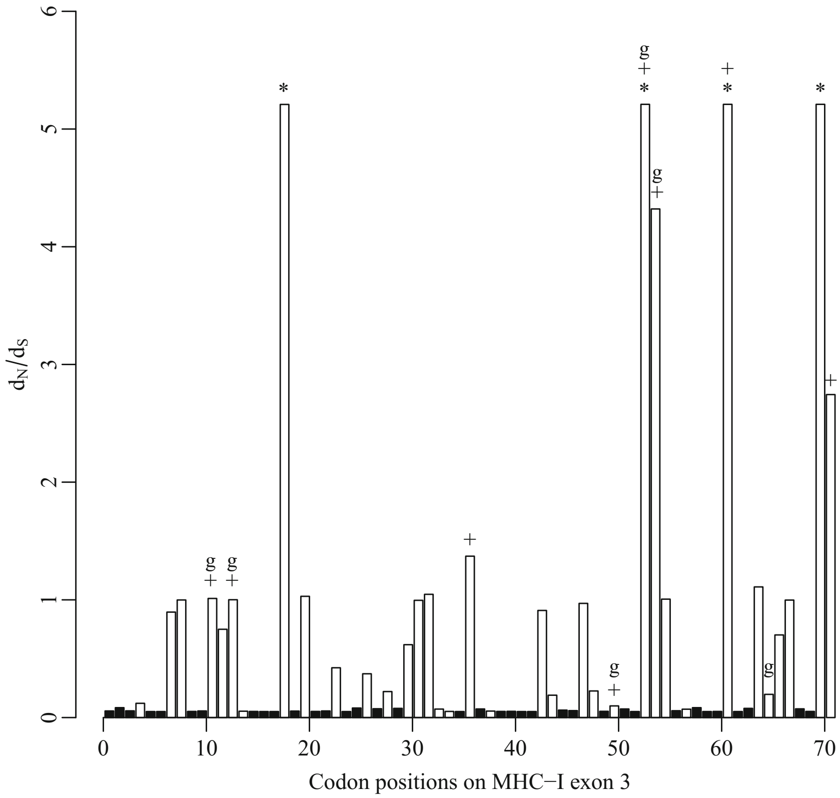

Fig. 3 The d_N/d_S ratio along MHC-I exon 3 in *Zonotrichia capensis*. Four significant peaks in d_N/d_S (indicated by *asterisks*) correspond to codon positions 18, 53, 61, and 70. Sites marked by a "+" indicate positively selected sites in>50 % of passerines examined (Alcaide et al. 2013). Sites marked by a "g" correspond to the peptide-binding region of *Gallus gallus* MHC (Wallny et al. 2006; Sepil et al. 2012). *White horizontal bars* represent polymorphic codons, while *black horizontal bars* represent invariant codons

PC2 was most strongly correlated with amino acid variation ($z1$–$z5$) at codon 18 (r=0.761).

The number of MHC alleles, proteins, and supertypes per individual and nucleotide diversity varied significantly by elevational zone (P_{allele}=0.017, $P_{protein}$=0.001, $P_{supertype}$=0.003, P_{π}<0.0001). In particular, middle elevation individuals possessed significantly more MHC alleles, proteins, and supertypes than low elevation individuals (P_{allele}=0.002, $P_{protein}$<0.001, $P_{supertype}$=0.005) and high elevation individuals (P_{allele}=0.007, $P_{protein}$<0.001, $P_{supertype}$=0.001, Table 2). Nucleotide diversity was also significantly higher in middle elevation populations relative to low (P<0.001, Table 2) or high elevation (P=0.004, Table 2). After controlling for the effect of elevation, MHC allele and supertype diversity per individual did not significantly vary by transect (P_{allele}=0.228,

Table 1 Proportion of segregating sites and nucleotide diversity for different MHC sequences or subsets of alleles

Sequence	Proportion of segregating sites	Nucleotide diversity
Entire MHC-1 exon 3	0.32	0.085±0.002
Non-PSS	0.29	0.070±0.001
PSS	0.75	0.302±0.029
Non-PBR	0.28	0.064±0.001
PBR	0.78	0.308±0.027
Clade 1 alleles	0.20	0.052±0.001
Clade 2 alleles	0.24	0.049±0.001

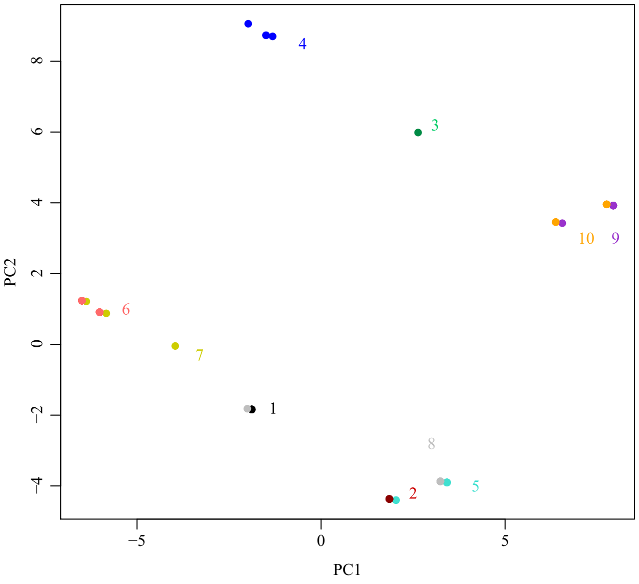

Fig. 4 First and second principal components for physicochemical properties of amino acids under positive selection. Each *dot* represents a unique amino acid sequence for positively selected sites. MHC supertypes are depicted as unique colors corresponding to colors on MHC phylogeny (1=7 alleles, 2=15 alleles, 3=1 allele, 4=4 alleles, 5=12 alleles, 6=31 alleles, 7=3 alleles, 8=6 alleles, 9=9 alleles, 10=10 alleles)

Table 2 Summary statistics of MHC-I exon 3 diversity for different *Zonotrichia capensis* populations

	Population					
	Elevational transect T1	Elevational transect T2	Elevational transect T3	Low elevation	Middle elevation	High elevation
Number of individuals	62	54	61	52	46	79
Total number MHC alleles	69	56	61	43	66	81
MHC alleles per individual	4.73±1.48	4.13±1.68	4.20±2.01	4.00±1.75	5.02±1.44	4.22±1.83
MHC proteins per individual	3.92±1.32	3.28±1.50	3.45±1.39	3.29±1.38	4.24±1.45	3.35±1.31
MHC supertypes per individual	3.48±1.11	3.06±1.17	3.07±1.01	3.04±1.17	3.70±1.09	3.04±1.01
Segregating sites	63	51	57	54	63	68
Nucleotide diversity	0.0847±0.0018	0.0812±0.0017	0.0848±0.0018	0.0827±0.0017	0.0869±0.0019	0.0860±0.0018

$P_{supertype}$=0.074). The number of MHC proteins per individual was significantly different across transects ($P_{protein}<$ 0.031) with elevated diversity at the low-latitude transect (T1). Nucleotide diversity was also significantly different across different transects (P_{π}<0.001) with substantially decreased nucleotide diversity in the middle-latitude transect (T2) relative to the low- and high-latitude transects. Across the elevational transects, 19 MHC alleles were only present in T1, seven were found exclusively on T2, and nine were found on T3. Across elevational zones, we found five MHC alleles only at low elevation, eight exclusively found in middle elevations, and 17 found only in high elevations.

MHC Phylogeny

We identified two clades of MHC sequences in the final tree and in all four replicate runs (Fig. 2), although most MHC alleles within these groups did not fall into distinct, well-resolved clusters. Consensus trees from two of the four independent runs produced identical topologies to Fig. 2 with similar nodal support, while the other two consensus trees were nearly identical, however with nodes A and B collapsed in one run and nodes C and D collapsed in the other (Fig. 2). For all other nodes, support values were similar to those in Fig. 2. Nucleotide diversity was significantly higher in clade 1 (π=0.052±0.001) compared to clade 2 (π=0.049±0.001, P<0.001).

Evidence of selection

Across paralagous MHC-I exon 3 sequences, d_N/d_S analyses supported a positive selection model for codon evolution (M2) over the nearly neutral codon substitution model (M1, likelihood ratio=87.4, P<0.001, Table 3). We identified four codons (positions 18, 53, 61, and 70) that showed signatures of positive selection (PSSs, P <0.01), corresponding to 5.6 % of amino acids in exon 3 (Fig. 3). Assuming conservation of the PBR positions between *Z. capensis* and *G. gallus*, codon 53 corresponds to a peptide-contact site on the α-helix of class I MHC (Fig. 3). Codons 53 and 61 also are PSSs in all 16 passerine species investigated by Alcaide et al. (2013), while codons 18 and 70 are not PSSs in other passerine species (Fig. 3). The putative PBR in *Z. capensis* was defined as those codon positions homologous with the *G. gallus* PBR (positions 11, 13, 50, 53, 54, and 65, Fig. 3). Nucleotide diversity within the putative PBR (π=0.308±0.027) was significantly higher than nucleotide diversity across the entire exon (π= 0.085±0.002, P<0.001) and outside of the PBR (π=0.065± 0.001, P<0.001, Table 1). In addition, the proportion of segregating sites was also elevated in the inferred PBR (S=0.78) compared to the entire exon (S=0.32) and sequences outside the inferred PBR (S=0.28, Table 1).

Discussion

Patterns of MHC variation

Variation at the MHC has been linked to important fitness-related traits, including disease resistance and secondary

Table 3 Summary of parameter estimates from CodeML output for two different models of nucleotide substitution

Model	lnL	Parameters
M1—nearly neutral	−2,016.7	ω_1=0.048, ω_2=1.0<br>p_1=0.366, p_2=0.634
M2—positive selection	−1,929.3	ω_1=0.048, ω_2=1.0, ω_3=6.176<br>p_1=0.541, p_2=0.205, p_3=0.254

ω_1 is the parameter estimate of d_N/d_S for sites under negative selection, ω_2 is the estimate of d_N/d_S for neutrally evolving sites, and ω_3 is the estimate of d_N/d_S for sites under positive selection. p_1, p_2, and p_3 correspond to the proportion of sites in each ω class. M2 is supported over M1 with a likelihood ratio value of 87.4

lnL log-likelihood value

sexual ornamentation (Ditchkoff et al. 2001; Bonneaud et al. 2006; Loiseau et al 2010; Dunn et al. 2013). In this study, we found substantial allelic diversity at a functionally important MHC-I exon in *Z. capensis*. We identified 98 putatively functional MHC-I exon 3 alleles distributed across a minimum of 6 loci. These MHC alleles correspond to 56 unique MHC protein sequences. The total number of alleles we identified (scaled by the number of genotyped individuals) is comparable to *Parus major* (758 alleles, 1,492 genotyped individuals; Sepil et al. 2012), *Carpodacus erythrinus* (189 alleles, 182 genotyped individuals; Promerová et al. 2012), *Halobaena caerulea* (156 alleles, 110 genotyped individuals; Strandh et al. 2012), and *Geothlypis trichas* (224 alleles, 44 genotyped individuals; Dunn et al. 2013); however, the number of MHC alleles per individual is lower in *Z. capensis* (4.36 alleles/individual) compared to these species (range 23.8–7.99 alleles/individual).

Nucleotide diversity at the PSSs and putative PBR of *Z. capensis* MHC is substantially higher than nucleotide diversity at the putative PBR in several species, such as *Cyanistes caeruleus* (π_{PBR}=0.14, Schut et al. 2011) and *Halobaena caerulea* (π_{PBR}=0.14, Strandh et al. 2011), but similar to the low levels of exon-wide MHC-I nucleotide diversity reported in these species (π_{exon}=0.06). Furthermore, most amino acids in *Z. capensis* MHC-I exon 3 are invariant (50.7 % of codons) or show d_N/d_S <1 (54.1 % of codons, Table 3). Thus, low nucleotide and amino acid variation in *Z. capensis* may reflect pervasive negative selection, which plays an important role in the evolution of MHC in some species (Yang and Swanson 2002). Historical population bottlenecks are capable of producing similar signatures of reduced MHC variation (Babik et al. 2009a; Sutton et al. 2011; Sutton et al. 2013). In New Zealand saddlebacks, the effects of a severe bottleneck event are manifested in decreased overall allelic diversity and allelic diversity per individual rather than nucleotide diversity (Sutton et al. 2013). Allele surfing following a population expansion may also create similar genetic signatures as population bottlenecks (e.g., low genetic variation; Excoffier et al. 2009). A recent phylogeographic study of *Z. capensis* revealed signatures of population expansion in Peruvian lineages following the Pleistocene glaciation (Lougheed et al. 2013), though the patterns were weak and locus specific. Alternatively, low MHC variation in nonmigratory populations, such as the *Z. capensis* populations in this study, may reflect adaptation to a simple parasite community (Møller and Erritzøe 1998). A screening of avian malaria in these *Z. capensis* populations revealed a single *Haemoproteus* lineage contributed to ~94 % of malaria infections (Jones et al. 2013), suggesting a relatively simple malaria parasite community associated with *Z. capensis*. The factors driving low exon-wide nucleotide variation (Fig. 3, Table 1) and per individual allele variation are unclear. However, the high nucleotide variation at the PSSs and inferred PBR in spite of low exon-wide variation is consistent with strong balancing selection on the MHC driven by variation at these amino acid positions.

Although we do not know the transcription levels of the putatively functional alleles, given that the majority of reads were 214 bp in length (61.4 %) and had an open reading frame suggests that they may be functional. The high proportion of initial reads that were excluded from analyses by our variant verification procedure (42.5 % of total) likely comprises pseudoalleles or PCR or sequencing errors. Other MHC studies using pyrosequencing discarded a similarly high proportion of reads presumed to represent either pseudoalleles or errors (Galan et al. 2010; Zagalska-Neubauer et al. 2010; Spurgin et al. 2011; Sepil et al. 2012). The high level of potential pseudogenes and the lack of structure on our MHC phylogeny are consistent with extensive gene duplication and pseudogenization in MHC.

Selection on MHC

We found strong signatures of historical positive selection at specific amino acid positions (PSSs) on MHC-I exon 3 (Fig. 3). One PSS (codon 53) corresponded to a peptide-binding site on the *G. gallus* MHC, which is 80–100 million years diverged from *Z. capensis* (Shetty et al. 1999), while the remaining three PSSs did not correspond to the *G. gallus* PBR. As a result of the substantial sequence divergence between passerines and chickens and their differences in MHC structure (Balakrishnan et al. 2010), the *Z. capensis* PBR is likely not perfectly homologous with the *G. gallus* PBR. However, it is possible that not all PSSs in *Z. capensis* correspond to the PBR. Some PSSs may correspond to non-PBR sites that serve important subsidiary roles, function as epistatic compensatory mutations as a result of changes in the PBR, or result from the stochastic mutational process. Furthermore, variation in MHC-I exon 3 will not encompass all functionally important variation in MHC-I because we did not sequence exon 2, which also comprises a portion of the PBR, or regulatory sequences. Nonetheless, the elevated nucleotide diversity and proportion of segregating sites (Table 1, Fig. 3) within the putative PBR suggest that these amino acids may be also functionally important. We identified ten MHC supertypes that share similar physicochemical properties at PSSs. Supertype clusters differed primarily by the physicochemical properties at codon positions 18 and 61, according to a principal components analysis (Fig. 4). MHC supertypes do not form distinct phylogenetic clusters (Fig. 2), which reflect either (i) repeated convergent evolution of MHC supertypes, (ii) that MHC supertypes we identified do not represent functionally important groups, or (iii) that we lack the appropriate phylogenetic resolution to observe distinct patterns.

Maintenance of diversity

Our data are consistent with historical balancing selection on *Z. capensis* MHC. The method of MHC sequencing we employed does not yield allele or genotype frequencies, so we cannot use traditional population genetic approaches to further investigate evidence of current selection. An approach comparing the geographic distribution of MHC variation to neutral variation would help elucidate whether selection on MHC is ongoing. Identifying associations between MHC alleles and parasite infection status or mate choice would further help to reveal the nature of selection on MHC.

Individuals sampled from intermediate elevations possess significantly more MHC alleles, proteins, and supertypes compared to those from high or low elevation populations (Table 2). We also observed significantly higher nucleotide diversity in middle elevation populations relative to low and high elevation populations. Thus, middle elevation populations may experience stronger balancing selection on MHC relative to low and high populations. MHC allelic and supertype diversity per individual did not significantly vary by transect after controlling for the effects of elevation, although MHC protein diversity per individual and nucleotide diversity were significantly affected by transect. Taken together, environmental heterogeneity seems to play a role in mediating selection dynamics on MHC. Differences in parasite composition, abundance, or virulence between elevational zones and transects may contribute to spatial variation in parasite-mediated selection and MHC composition. Indeed, Jones et al. (2013) found higher prevalence of avian malaria within *Z. capensis* at middle elevations and low latitudes. The patterns of malaria prevalence and MHC variation are consistent with intensified parasite-mediated selection in these environments, yet the precise selective mechanisms are unclear and warrant further investigation.

Conclusions

Our study reveals a high level of allelic variation in MHC-I exon 3 of a common South American passerine. Although we find low levels of allelic diversity per individual and exon-wide nucleotide diversity, we find extraordinarily high levels of nucleotide diversity within regions of the exon showing signatures of positive selection and those corresponding to the putative PBR. Variation in physicochemical properties of amino acids at the PSSs revealed ten functional MHC supertypes that do not cluster together on the MHC allele phylogeny, suggestive of either recurrent parallel evolution of these diverse supertypes or low phylogenetic resolution. Geographic variation in nucleotide diversity and the number of MHC alleles, proteins, and supertypes per individual is consistent with varying environmental selective pressures, perhaps related to malarial parasites. This study lays the groundwork for future research into the specific selective agents and mechanisms acting on MHC and provides important insight into the evolution of the MHC in a wild Neotropical passerine.

Acknowledgments This work was funded by the American Museum of Natural History Frank M. Chapman Fund, Sigma Xi Grant-In-Aid-Of-Research, the American Ornithologists' Union, and the University of Wyoming. Voucher specimens and pectoral muscle tissue samples of all of the specimens included in this study are accessioned at the Louisiana State University Museum of Natural Science (Baton Rouge), the Museo de Historia Natural, Universidad Nacional Mayor de San Marcos (Lima, Peru), and the Centro de Ornitologia y Biodiversidad (Lima, Peru). The Genome Sequencing and Analysis Core Resource at Duke University sequenced MHC amplicon libraries. We thank Amy Ellison for advice with designing MHC sequencing protocol. We thank Shawn M. Billerman, C. Alex Buerkle, Michael E. Dillon, James M. Maley, Melanie M. Murphy, and three anonymous reviewers for providing helpful comments on the manuscript.

References

Alcaide M, Edwards SV, Negro JJ (2007) Characterization, polymorphism, and evolution of MHC class II B genes in birds of prey. J Mol Evol 65:541–554

Alcaide M, Edwards SV, Cadahía L, Negro JJ (2009) MHC class I genes of birds of prey: isolation, polymorphism and diversifying selection. Conserv Genet 10:1349–1355

Alcaide M, Lui M, Edward SV (2013) Major histocompatibility complex class I evolution in songbirds: universal primers, rapid evolution and base compositional shifts in exon 3. Peer J 1:e86

Anisimova M, Nielsen R, Yang Z (2003) Effect of recombination on the accuracy of the likelihood method for detecting positive selection at amino acid sites. Genetics 164:1229–1236

Babik W, Pabijan M, Arntzen JW, Cogălniceanu D, Durka W, Radwan J (2009a) Long-term survival of a urodele amphibian despite depleted major histocompatibility complex variation. Mol Ecol 18:769–781

Babik W, Taberlet P, Ejsmond MJ, Radwan J (2009b) Next generation sequencers as a tool for genotyping of highly polymorphic multilocus MHC system. Mol Ecol Res 9:713–719

Babik W, Kawałko A, Wójcik JM, Radwan J (2012) Low major histocompatibility complex class I (MHC I) variation in the European Bison (*Bison bonasus*). J Hered 103:349–359

Balakrishnan CN, Ekblom R, Völker M, Westerdahl H, Godinez R, Kotkiewicz H, Burt DW, Graves T, Griffin DK, Warren WC, Edwards SV (2010) Gene duplication and fragmentation in the zebra finch major histocompatibility complex. BMC Biol 8:29

Bernatchez L, Landry C (2003) MHC studies in nonmodel vertebrates: what have we learned about natural selection in 15 years? J Evol Biol 16:363–377

Bjorkman PJ, Saper MA, Samraoui BSB, Bennett WS, Strominger JL, Wiley DC (1987) The foreign antigen binding site and T cell recognition regions of class I histocompatibility antigens. Nature 329:512–518

Bonneaud C, Sorci G, Morin V, Westerdahl H, Zoorob R, Wittzell H (2004) Diversity of Mhc class I and IIB genes in house sparrows (*Passer domesticus*). Immunogenetics 55:855–865

Bonneaud C, Pérez-Tris J, Federici P, Chastel O, Sorci G (2006) Major histocompatibility alleles associated with local resistance to malaria in a passerine. Evolution 60:383–389

Cheviron ZA, Brumfield RT (2009) Migration-selection balance and local adaptation of mitochondrial haplotypes in rufous-collared sparrow (*Zonotrichia capensis*) along an elevational gradient. Evolution 63:1593–1605

Cole RK (1968) Studies on the genetic resistance to Marek's disease. Avian Dis 12:9–28

Delany ME, Robinson CM, Goto RM, Miller MM (2009) Architecture and organization of chicken microchromosome 16: order of the NOR, MHC-Y, and MHC-B subregions. J Hered 100:507–514

Ditchkoff SS, Lochmiller RL, Masters RE, Hoofer SR, Van Den Bussche RA (2001) Major histocompatibility-complex-associated variation in secondary sexual traits of white tailed deer (*Odocoileus virginianus*): evidence for good-genes advertisement. Evolution 55:616–625

Doytchinova IA, Flower DR (2005) In silico identification of supertypes for class II MHCs. J Immunol 174:7085–7095

Dunn PO, Bollmer JL, Freeman-Gallant CR, Whittingham LA (2013) MHC variation is related to a sexually selected ornament, survival, and parasite resistance in common yellowthroats. Evolution 67:679–687

Edwards SV, Hedrick PW (1998) Evolution and ecology of MHC molecules: from genomics to sexual selection. Trends Ecol Evol 13: 305–311

Ekblom R, Stapley J, Ball AD, Birkhead T, Burke T, Slate J (2011) Genetic mapping of the major histocompatibility complex in the zebra finch (*Taeniopygia guttata*). Immunogenetics 63:523–530

Ellison A, Allainguillaume J, Girdwood S, Pachebat J, Peat KM, Wright P, Consuegra S (2012) Maintaining functional major histocompatibility complex diversity under inbreeding: the case of a selfing vertebrate. Proc R Soc B 279:5004–5013

Excoffier L, Foll M, Petit RJ (2009) Genetic consequences of range expansions. Annu Rev Ecol Evol Syst 40:481–501

Galan M, Guivier E, Caraux G, Charbonnel N, Cosson J-F (2010) A 454 multiplex sequencing method for rapid and reliable genotyping of highly polymorphic genes in large-scale studies. BMC Genomics 11:296

Gilles A, Meglécz E, Pech N, Ferreira S, Malausa T, Martin JF (2011) Accuracy and quality assessment of 454 GS-FLX Titanium pyrosequencing. BMC Genomics 12:245

Hackett SJ et al (2008) A phylogenomic study of birds reveals their evolutionary history. Science 320:1763–1768

Hammer J, Gallazzi F, Bono E, Karr RW, Guenot J, Valsasnini J, Nagy ZA, Sinigaglia FL (1995) Peptide binding specificity of HLA-DR4 molecules: correlation with rheumatoid arthritis association. J Exp Med 5:1847–1855

Hansen MM, Skaala O, Jensen LF, Bekkevold D, Mensberg KL (2007) Gene flow, effective population size and selection at major histocompatibility complex genes: brown trout in the Hardanger Fjord, Norway. Mol Ecol 16:1413–1425

Hedrick PW (2002) Pathogen resistance and genetic variation at MHC loci. Evolution 56(1902):1908

Hill AVS (1998) The immunogenetics of human infectious diseases. Ann Rev Immunol 16:593–617

Horton R, Wilming L, Rand V, Lovering RC, Bruford AE, Khodiyar VK, Lush MJ, Povey S, Talbot CC Jr, Wright MW, Wain HM, Trowsdale J, Ziegler A, Beck S (2004) Gene map of the extended human MHC. Nat Rev Genet 5:889–899

Hughes AL, Nei M (1988) Patterns of nucleotide substitution at major histocompatibility complex class I loci reveals overdominant selection. Nature 335:167–170

Hughes AL, Nei M (1989) Evolution of the major histocompatibility complex: independent origin of nonclassical class I genes in different groups of mammals. Mol Biol Evol 6:559–579

Hughes CR, Miles S, Walbroehl JM (2008) Support for the minimal essential MHC hypothesis: a parrot with a single, highly polymorphic MHC class II B gene. Immunogenetics 60:219–231

Jombart T (2008) adegenet: a R package for the multivariate analysis of genetic markers. Bioinformatics 24:1403–1405

Jombart T, Devillard S, Balloux F (2010) Discriminant analysis of principal components: a new method for the analysis of genetically structured populations. BMC Genet 11:94

Jones MR, Cheviron ZA, Carling MD (2013) Spatial patterns of avian malaria prevalence in *Zonotrichia capensis* on the western slope of the Peruvian Andes. J Parasitol 99:903–905

Kaufman J, Milne S, Göbel TWF, Walker BA, Jacob JP, Auffray C, Zoorob R, Beck S (1999) The chicken B locus is a minimal essential major histocompatibility complex. Nature 40:923–925

Klein J, Satta Y, O'hUigin C, Takahata N (1993) The molecular descent of the major histocompatibility complex. Ann Rev Immunol 11: 269–295

Kloch A, Babik W, Bajer A, Siński A, Radwan J (2010) Effects of an MHC-DRB genotype and allele number on the load of gut parasites in the bank vole Myodes glareolus. Mol Ecol 19:255–265

Kryazhimskiy S, Plotkin JB (2008) The population genetics of dN/dS. PLoS Genet 4:e1000304

Lamaze FC, Pavey SA, Normandeau E, Roy G, Garant D, Bernatchez L (2014) Natural and selective processes shape MHC gene diversity and expression in stocked brook charr populations (*Salvelinus fontinalis*). Mol Ecol 23:1730–1748

LePage KT, Miller MM, Briles WE, Taylor RL Jr (2000) Rfp-Y genotype affects the fate of Rous sarcomas in B^2 B^5 chickens. Immunogenetics 51:751–754

Loiseau C, Zoorob R, Robert A, Chastel O, Julliard R, Sorci G (2010) *Plasmodium relictum* infection and MHC diversity in the House Sparrow. Proc R Soc B 278:1264–1272

Lougheed SC, Campagna L, Dávila JA, Tubaro PL, Lijtmaer DA, Handford P (2013) Continental phylogeography of an ecologically and morphologically diverse songbird, *Zonotrichia capensis*. BMC Evol Biol 13:58

Matsumura M, Fremont DH, Peterson PA, Wilson IA (1992) Emerging principles for the recognition of peptide antigens by MHC class I molecules. Science 257:927–934

Miller HC, Bowker-Wright G, Kharkrang M, Ramstad K (2011) Characterisation of class II B MHC genes from a ratite bird, the little spotted kiwi (*Apteryx owenii*). Immunogenetics 63:223–233

Møller AP, Erritzøe J (1998) Host immune defence and migration in birds. Evol Ecol 12:945–953

Nadachowska-Brzyska K, Zieliński P, Radwan J, Babik W (2012) Interspecific hybridization increases MHC class II diversity in two sister species of newts. Mol Ecol 21:887–906

Ophir R, Graur D (1997) Patterns and rates of indel evolution in processed pseudogenes from humans and murids. Gene 205:191–202

Paradis E (2010) pegas: an R package for population genetics with an integrated-modular approach. Bioinformatics 26:419–420

Penn DJ, Damjanovich K, Potts WK (2002) MHC heterozygosity confers a selective advantage against multiple strain infections. Proc Natl Acad Sci 99:11260–11264

Promerová M, Babik W, Bryja J, Albrecht T, Stuglik M, Radwan J (2012) Evaluation of two approaches to genotyping major histocompatibility complex class I in passerine CE SSCP and 454 pyrosequencing. Mol Ecol Res 12:285–292

R Development Core Team (2013) R: a language and environment for statistical computing. R Foundation for Statistical Computing, Vienna, Austria

Reusch TBH, Häberli MA, Aeschlimann PB, Milinski M (2001) Female sticklebacks count alleles in a strategy of sexual selection explaining MHC polymorphism. Nature 414:300–302

Ronquist F, Huelsenbeck JP (2003) MrBayes 3: Bayesian phylogenetic inference under mixed models. Bioinformatics 19:1572–1574

Schulenberg TS, Stotz DF, Lane DF, O'Neill JP, Parker TA III (2007) Birds of Peru. Princeton University Press, Princeton, p 656

Schut E, Rivero-de Aguilar J, Merino S, Magrath MJL, Komdeur J, Westerdahl H (2011) Characterization of MHC-I in the blue tit (*Cyanistes caeruleus*) reveals low levels of genetic diversity and trans-population evolution across European populations. Immunogenetics 63:531–542

Sepil I, Moghadam HK, Huchard E, Sheldon BC (2012) Characterization and 45 pyrosequencing of major histocompatibility complex class I genes in the great tit reveal complexity in a passerine system. BMC Evol Biol 12:68

Shetty S, Griffin DK, Graves JA (1999) Comparative painting reveals strong chromosome homology over 80 million years of bird evolution. Chromosom Res 7:289–295

Shiina T, Shimizu S, Hosomichi K, Kohara S, Watanabe S, Hanzawa K, Beck S, Kulski JK, Inoko H (2004) Comparative genomic analysis of two avian (quail and chicken) MHC regions. J Immunol 172: 6751–6763

Shiina T, Briles WE, Goto RM, Hosomichi K, Yanagiya K, Shimizu S, Inoko H, Miller MM (2007) Extended gene map reveals tripartite motif, C-type lectin, and Ig superfamily type genes within a subregion of the chicken MHC-B affecting infectious disease. J Immunol 178:7162–7172

Spurgin LG, Richardson DS (2010) How pathogens drive genetic diversity: MHC, mechanisms, and misunderstandings. Proc R Soc B 277: 979–988

Spurgin LG, van Oosterhout C, Illera JC, Bridgett S, Gharbi K, Emerson BC, Richardson DS (2011) Gene conversion rapidly generates major histocompatibility complex diversity in recently founded bird populations. Mol Ecol 20:5213–5225

Stager M, Cerasale DJ, Dor R, Winkler DW, Cheviron ZA (2014) Signatures of natural selection in the mitochondrial genomes of *Tachycineta* swallows and their implications for latitudinal patterns of the 'pace of life'. Gene. In press.

Stiebens VA, Merino SE, Chain FJJ, Eizaguirre C (2013) Evolution of MHC class I genes in the endangered loggerhead sea turtle (*Caretta caretta*) revealed by 454 amplicon sequencing. BMC Evol Biol 13:95

Strandh M, Lannefors M, Bonadonna F, Westerdahl H (2011) Characterization of MHC class I and II genes in a subantarctic seabird, the blue petrel, *Halobaena caerulea* (Procellariiformes). Immunogenetics 63:653–666

Strandh M, Westerdahl H, Pontarp M, Canbäck B, Dubois MP, Miquel C, Taberlet P, Bonadonna F (2012) Major histocompatibility complex class II compatibility, but not class I, predicts mate choice in a bird with highly developed olfaction. Proc R Soc B 279:4457–4463

Stuglik MT, Radwan J, Babik W (2011) jMHC: software assistant for multilocus genotyping of gene families using next-generation amplicon sequencing. Mol Ecol Res 11:739–742

Sutton JT, Nakagawa S, Robertson BC, Jamieson IG (2011) Disentangling the roles of natural selection and genetic drift in shaping variation at MHC immunity genes. Mol Ecol 20: 4408–4420

Sutton JT, Robertson BC, Grueber CE, Stanton JL, Jamieson IG (2013) Characterization of MHC class II B polymorphism in bottlenecked New Zealand saddlebacks reveals low levels of genetic diversity. Immunogenetics 65:619–633

Takahashi K, Rooney AP, Nei M (2000) Origins and divergence times of mammalian class II MHC gene clusters. J Hered 91:198–204

Takahata N, Nei M (1990) Allelic genealogy under overdominant and frequency dependent selection and polymorphism of major histocompatibility complex loci. Genetics 124:967–978

Tao N, Bruno WJ, Abfalterer W, Moret BME, Leitner, T, Kuiken C (2005) FINDMODEL: a tool to select the best-fit model of nucleotide substitution. http://hcv.lanl.gov/content/sequence/findmodel/findmodel.html

von-Schantz T, Wittzell H, Göransson G, Grahn M, Persson K (1996) MHC genotype and male ornamentation: genetic evidence for the Hamilton-Zuk hypothesis. Proc R Soc B 263:265–271

Wakenell PS, Miller MM, Goto RM, Gauderman WJ, Briles WE (1996) Association between the Rfp-Y haplotype and the incidence of Marek's disease in chickens. Immunogenetics 44:242–245

Wallny H-J, Avila D, Hunt LG, Powell TJ, Riegert P, Salomonsen J, Skjødt K, Vainio O, Vilbois F, Wiles MV, Kaufman J (2006) Peptide motifs of the single dominantly expressed class I molecule explain the striking MHC determined response to Rous sarcoma virus in chickens. Proc Natl Acad Sci 103:1434–1439

Wang Y, Qiu M, Yang J, Zhao X, Wang Y, Zhu Q, Liu Y (2014) Sequence variations of the MHC class I gene exon 2 and exon 3 between infected and uninfected chicken challenged with Marek's disease virus. Infect Genet Evol 21:103–109

Wegner KM, Reusch TBH, Kalbe M (2003) Multiple parasites are driving major histocompatibility complex polymorphism in the wild. J Evol Biol 16:224–232

Westerdahl H, Wittzell H, von-Schantz T (1999) Polymorphism and transcription of Mhc class I genes in a passerine bird, the great reed warbler. Immunogenetics 49:158–170

Westerdahl H, Waldenström J, Hansson B, Hasselquist D, von-Schantz T, Bensch S (2005) Associations between malaria and MHC genes in a migratory songbird. Proc R Soc B 272:151–1518

Westerdahl H (2007) Passerine MHC: genetic variation and disease resistance in the wild. J Ornithol 148:S469–S477

Westerdahl H, Asghar M, Hasselquist D, Bensch S (2012) Quantitative disease resistance: to better understand parasite-mediated selection on major histocompatibility complex. Proc R Soc B 279:577–584

Wilson DJ, McVean G (2006) Estimating diversifying selection and functional constraint in the presence of recombination. Genetics 172:1411–1425

Wiseman RW, Karl JA, Bimber BN, O'Leary CE, Lank SM, Tuscher JJ, Detmer AM, Bouffard P, Levenkova N, Turcotte CL, Szekeres E Jr, Wright C, Harkins T, O'Connor DH (2009) Major histocompatibility complex genotyping with massively parallel pyrosequencing. Nat Med 15:1322–1327

Wucherpfennig KW, Yu B, Bhol K, Monos DS, Argyris E, Karr RW, Ahmed AR, Strominger JL (1995) Structural basis for major histocompatibility complex (MHC)-linked susceptibility to autoimmunity: charged residues of a single MHC binding pocket confer selective presentation of self-peptides in pemphigus vulgaris. Proc Natl Acad Sci 92:11935–11939

Yang Z (2007) PAML 4: phylogenetic analysis by maximum likelihood. Mol Biol Evol 24:1586–1591

Yang Z, Swanson WJ (2002) Codon-substitution models to detect adaptive evolution that account for heterogenous selective pressures among site classes. Mol Biol Evol 19:49–57

Zagalska-Neubauer M, Babik W, Stuglik M, Gustafsson L, Cichoń M, Radwan J (2010) 454 sequencing reveals extreme complexity of the class II major histocompatibility complex in the collared flycatcher. BMC Evol Biol 10:395
