# Supplementary material for: Spatially variable coevolution between a haemosporidian parasite and the MHC of a widely distributed passerine
Source: Ecol Evol. 2015 Feb 6;5(5):1045–60. doi: 10.1002/ece3.1391 (PMC4364819; doi:10.1002/ece3.1391)
Supplement: Supplementary file 4 [file ece30005-1045-sd4.docx]

**Table S1.** *Zonotrichia capensis* specimen data.

| ID# | Locality code | Collection date | Sex | % skull ossification | Infection status |
| --- | --- | --- | --- | --- | --- |
| 48233 | T1-1 | 8/8/2004 | Male | 100 |  |
| 48246 | T1-1 | 12/8/2004 | Female | 100 |  |
| 48254 | T1-1 | - | - | - |  |
| 48255 | T1-1 | 12/8/2004 | Male | 100 |  |
| 48256 | T1-1 | 12/8/2004 | Male | 100 |  |
| 48257 | T1-1 | 12/8/2004 | Male | 100 |  |
| 48258 | T1-1 | - | - | - |  |
| 48259 | T1-1 | 12/8/2004 | Male | 100 |  |
| 48260 | T1-1 | 12/8/2004 | Male | 100 | *Haemoproteus* sp. KC480265 |
| 48261 | T1-1 | 12/8/2004 | Male | 100 | *Haemoproteus* sp. KC480265 |
| 52248 | T1-2 | - | - | - | *Haemoproteus* sp. KC480265 |
| 52249 | T1-2 | 31/5/2005 | - | - | *Haemoproteus* sp. KC480265 |
| 52252 | T1-2 | 31/5/2005 | Female |  | *Haemoproteus* sp. KC480265 |
| 52253 | T1-2 | - | - | - | *Haemoproteus* sp. KC480265 |
| 52254 | T1-2 | 31/5/2005 | Male | 25 | *Haemoproteus* sp. KC480265 |
| 52255 | T1-2 | 31/5/2005 | Male | 100 | *Haemoproteus* sp. KC480265 |
| 52260 | T1-2 | 1/6/2005 | Female | 100 | *Haemoproteus* sp. KC480265 |
| 52261 | T1-2 | 1/6/2005 | Male | - | *Haemoproteus* sp. KC480265 |
| 52262 | T1-2 | 2/6/2005 | Male | 100 | *Haemoproteus* sp. KC480265 |
| 52263 | T1-2 | 2/6/2005 | Male | 100 | *Haemoproteus* sp. KC480265 |
| 52265 | T1-2 | 2/6/2005 | - | - | *Haemoproteus* sp. KC480265 |
| 52266 | T1-2 | 2/6/2005 | Male | - | *Haemoproteus* sp. KC480265 |
| 52267 | T1-2 | 3/6/2005 | Male | 100 | *Haemoproteus* sp. KC480265 |
| 52268 | T1-2 | 3/6/2005 | Male | 100 | *Haemoproteus* sp. KC480265 |
| 52269 | T1-2 | 3/6/2005 | Male | 100 | *Haemoproteus* sp. KC480265 |
| 52270 | T1-2 | 3/6/2005 | Female | 100 | *Haemoproteus* sp. KC480265 |
| 52271 | T1-2 | 3/6/2005 | Male | - | *Haemoproteus* sp. KC480265 |
| 56627 | T1-2 | 15/2/2007 | Male | 100 | *Haemoproteus* sp. KC480265 |
| 56628 | T1-2 | 15/2/2007 | Female | 50 | *Haemoproteus* sp. KC480265 |
| 56629 | T1-2 | 15/2/2007 | Female | 100 | *Haemoproteus* sp. KC480265 |
| 56630 | T1-2 | 15/2/2007 | Male | 75 |  |
| 56634 | T1-2 | 15/2/2007 | Female | 100 | *Haemoproteus* sp. KC480265 |
| 56635 | T1-2 | 15/2/2007 | Female | - |  |
| 56636 | T1-2 | 15/2/2007 | Female | - | *Haemoproteus* sp. KC480265 |
| 56637 | T1-2 | 15/2/2007 | Male | - | *Haemoproteus* sp. KC480265 |
| 52310 | T1-3 | 5/6/2005 | Male | 100 | *Haemoproteus* sp. KC480265 |
| 52323 | T1-3 | 5/6/2005 | - | 100 |  |
| 52324 | T1-3 | 5/6/2005 | Female | 100 | *Haemoproteus* sp. KC480265 |
| 52327 | T1-3 | 5/6/2005 | - | - | *Haemoproteus* sp. KC480265 |
| 52311 | T1-4 | 5/6/2005 | Female | 100 |  |
| 52277 | T1-4 | 5/6/2005 | Male | 100 |  |
| 52278 | T1-4 | 5/6/2005 | Male | 100 | *Haemoproteus* sp. KC480265 |
| 52312 | T1-4 | 5/6/2005 | - | 100 |  |
| 52313 | T1-4 | 5/6/2005 | Male | 100 |  |
| 52314 | T1-4 | 5/6/2005 | Male | 100 |  |
| 52315 | T1-4 | 5/6/2005 | Male | 100 |  |
| 52344 | T1-4 | 5/6/2005 | - | - |  |
| 49604 | T1-4 | 17/8/2004 | Male | 100 |  |
| 49596 | T1-5 | - | - | - |  |
| 52289 | T1-5 | 7/6/2005 | Female | 100 |  |
| 52286 | T1-5 | 7/6/2005 | Female | 100 |  |
| 52287 | T1-5 | - | - | - |  |
| 52288 | T1-5 | 7/6/2005 | Male | 100 |  |
| 52290 | T1-5 | 7/6/2005 | Male | 100 |  |
| 52291 | T1-5 | 7/6/2005 | Female | 100 |  |
| 52292 | T1-5 | 7/6/2005 | Male | 100 |  |
| 52293 | T1-5 | 7/6/2005 | Male | - |  |
| 52304 | T1-5 | 6/6/2005 | Female | 25 |  |
| 52305 | T1-5 | 6/6/2005 | Female | 100 |  |
| 52306 | T1-5 | 6/6/2005 | Male | 100 |  |
| 52343 | T1-5 | 6/6/2005 | Male | 100 |  |
| 56622 | T1-5 | 11/2/2007 | Female | 100 |  |
| 56623 | T1-5 | 9/2/2007 | Male | 50 |  |
| 56624 | T1-5 | 9/2/2007 | Male | 100 |  |
| 56625 | T1-5 | 13/2/2007 | Male | 100 |  |
| 56626 | T1-5 | 13/2/2007 | - | - |  |
| 56631 | T1-5 | 9/2/2007 | Male | 100 |  |
| 56632 | T1-5 | 13/2/2007 | Female | 100 |  |
| 56633 | T1-5 | 13/2/2007 | Male | 100 |  |
| 52328 | T2-1 | 14/6/2005 | Female | 100 |  |
| 52329 | T2-1 | 14/6/2005 | Female | 100 |  |
| 52330 | T2-1 | 14/6/2005 | Male | 50 |  |
| 52331 | T2-1 | 14/6/2005 | Male | - |  |
| 52332 | T2-1 | 14/6/2005 | Female | 25 | *Haemoproteus* sp. KC480266 |
| 52333 | T2-1 | 14/6/2005 | Male | 100 |  |
| 52334 | T2-1 | 14/6/2005 | Male | 25 |  |
| 52353 | T2-1 | 15/6/2005 | Female | 100 |  |
| 52354 | T2-1 | 15/6/2005 | Female | 100 |  |
| 52355 | T2-1 | 15/6/2005 | Male | 100 |  |
| 52356 | T2-1 | 15/6/2005 | Female | 100 |  |
| 52357 | T2-1 | 15/6/2005 | Female | 100 |  |
| 52358 | T2-1 | 15/6/2005 | Male | 100 |  |
| 52359 | T2-1 | 15/6/2005 | - | 100 |  |
| 52360 | T2-1 | 15/6/2005 | - | - |  |
| 52362 | T2-1 | 14/6/2005 | Male | 100 | *Plasmodium* sp. KC480267 |
| 56541 | T2-2 | 21/2/2007 | Male | 0 |  |
| 56542 | T2-2 | 21/2/2007 | Male | 100 |  |
| 56543 | T2-2 | 21/2/2007 | Male | 100 |  |
| 56544 | T2-2 | 21/2/2007 | - | 100 |  |
| 56545 | T2-2 | 21/2/2007 | Male | 100 |  |
| 56546 | T2-2 | 21/2/2007 | - | 100 |  |
| 56547 | T2-2 | 20/2/2007 | Male | 100 |  |
| 56548 | T2-2 | 20/2/2007 | Male | 100 |  |
| 56549 | T2-2 | 21/2/2007 | Male | 100 |  |
| 56569 | T2-3 | 23/1/2007 | Female | 100 | *Haemoproteus* sp. KC480265 |
| 56570 | T2-3 | 24/1/2007 | Male | 100 |  |
| 56571 | T2-3 | 24/1/2007 | Male | 100 |  |
| 56572 | T2-3 | 24/1/2007 | Male | 100 |  |
| 56553 | T2-4 | 22/1/2007 | Male | 100 | *Haemoproteus* sp. KC480265 |
| 56554 | T2-4 | 22/1/2007 | Male | 100 | *Haemoproteus* sp. KC480265 |
| 56555 | T2-4 | 22/1/2007 | Female | 75 |  |
| 56556 | T2-4 | 22/1/2007 | Female | 75 |  |
| 56557 | T2-4 | 22/1/2007 | Female | 75 | *Haemoproteus* sp. KC480265 |
| 56558 | T2-4 | 22/1/2007 | Male | 100 |  |
| 56559 | T2-4 | 22/1/2007 | Female | 100 |  |
| 56560 | T2-4 | 23/1/2007 | Male | 100 |  |
| 56561 | T2-4 | 23/1/2007 | Female | 100 | *Haemoproteus* sp. KC480265 |
| 56562 | T2-4 | 23/1/2007 | Male | 10 |  |
| 56563 | T2-4 | 23/1/2007 | Female | 100 |  |
| 56564 | T2-4 | 23/1/2007 | Male | - |  |
| 56565 | T2-4 | 23/1/2007 | Male | 100 | *Haemoproteus* sp. KC480265 |
| 56566 | T2-4 | 23/1/2007 | - | - |  |
| 56567 | T2-4 | 23/1/2007 | Male | 100 | *Haemoproteus* sp. KC480265 |
| 56568 | T2-4 | 23/1/2007 | Male | 100 |  |
| 52432 | T2-5 | 26/6/2005 | Female | - |  |
| 52433 | T2-5 | 26/6/2005 | Male | 25 |  |
| 52434 | T2-5 | 26/6/2005 | Male | 0 |  |
| 52435 | T2-5 | 26/6/2005 | Male | 0 |  |
| 52436 | T2-5 | 26/6/2005 | Female | 100 |  |
| 52437 | T2-5 | 26/6/2005 | - | - |  |
| 52405 | T2-5 | 26/6/2005 | Female | 10 |  |
| 52406 | T2-5 | 26/6/2005 | - | 10 |  |
| 52440 | T2-5 | 26/6/2005 | Female | 10 |  |
| 52415 | T3-1 | 19/6/2005 | Male | - |  |
| 52416 | T3-1 | 19/6/2005 | Male | 100 |  |
| 52417 | T3-1 | 19/6/2005 | Male | 100 |  |
| 52418 | T3-1 | 19/6/2005 | Female | 10 |  |
| 52419 | T3-1 | 19/6/2005 | Female | 5 |  |
| 52421 | T3-1 | 19/6/2005 | Female | 10 |  |
| 52422 | T3-1 | 19/6/2005 | Male | - |  |
| 52423 | T3-1 | 19/6/2005 | Female | 10 |  |
| 52424 | T3-1 | 19/6/2005 | Female | 0 |  |
| 52341 | T3-2 | 19/6/2005 | Male | 100 |  |
| 52369 | T3-2 | 19/6/2005 | Male | 100 |  |
| 52370 | T3-2 | 19/6/2005 | Female | 100 |  |
| 52371 | T3-2 | 19/6/2005 | Female | 100 |  |
| 52375 | T3-2 | 19/6/2005 | Male | 100 |  |
| 52425 | T3-3 | 21/6/2005 | Male | 25 |  |
| 52426 | T3-3 | 21/6/2005 | Male | 50 |  |
| 52430 | T3-3 | 21/6/2005 | - | 100 |  |
| 52431 | T3-3 | 21/6/2005 | - | 5 | *Haemoproteus* sp. KC480265 |
| 52382 | T3-4 | 20/6/2005 | Male | 75 |  |
| 52383 | T3-4 | 20/6/2005 | Female | 100 | *Haemoproteus* sp. KC480265 |
| 52399 | T3-4 | 21/6/2005 | Male | 10 |  |
| 52396 | T3-4 | 20/6/2005 | Male | 100 | *Haemoproteus* sp. KC480265 |
| 52400 | T3-4 | - | - | - |  |
| 52452 | T3-4 | - | - | - | *Haemoproteus* sp. KC480265 |
| 52388 | T3-5 | 22/6/2005 | Male | 100 | *Haemoproteus* sp. KC480265 |
| 52389 | T3-5 | 22/6/2005 | - | - | *Plasmodium* sp. KC480268 |
| 52390 | T3-5 | 22/6/2005 | - | - | *Haemoproteus* sp. KC480265 |
| 52401 | T3-5 | 22/6/2005 | Female | 10 |  |
| 52404 | T3-5 | 22/6/2005 | Female | 10 | *Haemoproteus* sp. KC480265 |
| 52402 | T3-5 | 22/6/2005 | Male | 90 | *Haemoproteus* sp. KC480265 |
| 52443 | T3-6 | 24/6/2005 | Female | 100 |  |
| 52444 | T3-6 | 24/6/2005 | Male | 100 |  |
| 52445 | T3-6 | 24/6/2005 | Male | 100 | *Haemoproteus* sp. KC480265 |
| 52446 | T3-6 | 24/6/2005 | - | 100 | *Haemoproteus* sp. KC480265 |
| 52447 | T3-6 | 24/6/2005 | Male | 100 |  |
| 52448 | T3-6 | 24/6/2005 | - | 5 |  |
| 52449 | T3-6 | 24/6/2005 | Male | - |  |
| 56573 | T3-7 | 1/2/2007 | Male | 100 |  |
| 56574 | T3-7 | 1/2/2007 | Male | 100 |  |
| 56575 | T3-7 | 1/2/2007 | - | - |  |
| 56576 | T3-7 | 1/2/2007 | Female | 100 |  |
| 56577 | T3-7 | 1/2/2007 | Male | 100 |  |
| 56578 | T3-7 | 1/2/2007 | Male | 100 |  |
| 56579 | T3-7 | 1/2/2007 | Male | 100 |  |
| 56580 | T3-7 | 1/2/2007 | Male | - |  |
| 56581 | T3-7 | 1/2/2007 | Female | 100 |  |
| 56582 | T3-7 | 1/2/2007 | Male | 10 |  |
| 56583 | T3-8 | 29/1/2007 | Female | 100 |  |
| 56584 | T3-8 | 29/1/2007 | Female | 100 |  |
| 56585 | T3-8 | 29/1/2007 | Male | 100 |  |
| 56586 | T3-8 | 29/1/2007 | Male | 100 |  |
| 56587 | T3-8 | 29/1/2007 | Male | - |  |
| 56588 | T3-8 | 29/1/2007 | Male | 100 |  |
| 56597 | T3-8 | 30/1/2007 | Male | 100 |  |
| 56598 | T3-8 | 30/1/2007 | Male | 100 |  |
| 56599 | T3-8 | 30/1/2007 | Female | 100 |  |
| 56600 | T3-8 | 30/1/2007 | Male | - |  |
| 56601 | T3-8 | 30/1/2007 | Female | 50 |  |
| 56602 | T3-8 | 30/1/2007 | Male | 100 |  |
| 56603 | T3-8 | 30/1/2007 | Female | 100 |  |
| 56605 | T3-8 | 30/1/2007 | Female | 100 |  |

**Table S2.** Sampling locality information.

| Locality code | Latitude | Longitude | Elevation (m) |
| --- | --- | --- | --- |
| T1-1 | 11.40°S | 77.24°W | 450 |
| T1-2 | 11.73°S | 76.70°W | 2000 |
| T1-3 | 11.69°S | 76.16°W | 3300 |
| T1-4 | 11.63°S | 76.44°W | 3800 |
| T1-5 | 11.06°S | 76.16°W | 4150 |
| T2-1 | 13.84°S | 76.21°W | 130 |
| T2-2 | 13.66°S | 75.77°W | 620 |
| T2-3 | 13.57°S | 75.52°W | 1375 |
| T2-4 | 13.61°S | 75.34°W | 3100 |
| T2-5 | 13.35°S | 74.95°W | 4030 |
| T3-1 | 18.16°S | 70.67°W | 5 |
| T3-2 | 17.99°S | 70.19°W | 720 |
| T3-3 | 17.66°S | 70.08°W | 2750 |
| T3-4 | 17.54°S | 70.34°W | 3050 |
| T3-5 | 17.36°S | 70.14°W | 3300 |
| T3-6 | 17.23°S | 70.26°W | 3725 |
| T3-7 | 16.64°S | 69.44°W | 4110 |
| T3-8 | 15.89°S | 69.87°W | 3820 |

**Table S3.** Translation table between MHC alleles, proteins, and supertypes.

| **Alleles** | **Proteins** | **Supertypes** |
| --- | --- | --- |
| *ZocaU*01* | P1 | S1 |
| *ZocaU*02* | P2 | S6 |
| *ZocaU*03* | P3 | S10 |
| *ZocaU*04* | P2 | S6 |
| *ZocaU*05* | P4 | S9 |
| *ZocaU*06* | P4 | S9 |
| *ZocaU*07* | P5 | S6 |
| *ZocaU*08* | P6 | S4 |
| *ZocaU*09* | P7 | S6 |
| *ZocaU*10* | P8 | S3 |
| *ZocaU*11* | P3 | S10 |
| *ZocaU*12* | P9 | S10 |
| *ZocaU*13* | P10 | S2 |
| *ZocaU*14* | P2 | S6 |
| *ZocaU*15* | P2 | S6 |
| *ZocaU*16* | P11 | S7 |
| *ZocaU*17* | P12 | S10 |
| *ZocaU*18* | P13 | S5 |
| *ZocaU*19* | P13 | S5 |
| *ZocaU*20* | P14 | S6 |
| *ZocaU*21* | P15 | S2 |
| *ZocaU*22* | P16 | S8 |
| *ZocaU*23* | P17 | S5 |
| *ZocaU*24* | P2 | S6 |
| *ZocaU*25* | P18 | S7 |
| *ZocaU*26* | P5 | S6 |
| *ZocaU*27* | P19 | S6 |
| *ZocaU*28* | P4 | S9 |
| *ZocaU*29* | P5 | S6 |
| *ZocaU*30* | P20 | S2 |
| *ZocaU*31* | P21 | S8 |
| *ZocaU*32* | P7 | S6 |
| *ZocaU*33* | P15 | S2 |
| *ZocaU*34* | P10 | S2 |
| *ZocaU*35* | P22 | S5 |
| *ZocaU*36* | P12 | S10 |
| *ZocaU*37* | P23 | S9 |
| *ZocaU*38* | P2 | S6 |
| *ZocaU*39* | P3 | S10 |
| *ZocaU*40* | P24 | S5 |
| *ZocaU*41* | P10 | S2 |
| *ZocaU*42* | P3 | S10 |
| *ZocaU*43* | P25 | S9 |
| *ZocaU*44* | P26 | S8 |
| *ZocaU*45* | P27 | S8 |
| *ZocaU*46* | P28 | S4 |
| *ZocaU*47* | P29 | S2 |
| *ZocaU*48* | P10 | S2 |
| *ZocaU*49* | P4 | S9 |
| *ZocaU*50* | P1 | S1 |
| *ZocaU*51* | P4 | S9 |
| *ZocaU*52* | P2 | S6 |
| *ZocaU*53* | P5 | S6 |
| *ZocaU*54* | P5 | S6 |
| *ZocaU*55* | P30 | S2 |
| *ZocaU*56* | P23 | S9 |
| *ZocaU*57* | P31 | S10 |
| *ZocaU*58* | P32 | S4 |
| *ZocaU*59* | P12 | S10 |
| *ZocaU*60* | P30 | S2 |
| *ZocaU*61* | P6 | S4 |
| *ZocaU*62* | P33 | S5 |
| *ZocaU*63* | P34 | S5 |
| *ZocaU*64* | P35 | S6 |
| *ZocaU*65* | P36 | S10 |
| *ZocaU*66* | P1 | S1 |
| *ZocaU*67* | P10 | S2 |
| *ZocaU*68* | P2 | S6 |
| *ZocaU*69* | P2 | S6 |
| *ZocaU*70* | P37 | S8 |
| *ZocaU*71* | P2 | S6 |
| *ZocaU*72* | P38 | S1 |
| *ZocaU*73* | P2 | S6 |
| *ZocaU*74* | P2 | S6 |
| *ZocaU*75* | P39 | S1 |
| *ZocaU*76* | P40 | S6 |
| *ZocaU*77* | P41 | S5 |
| *ZocaU*78* | P42 | S8 |
| *ZocaU*79* | P43 | S2 |
| *ZocaU*80* | P10 | S2 |
| *ZocaU*81* | P44 | S1 |
| *ZocaU*82* | P45 | S7 |
| *ZocaU*83* | P41 | S5 |
| *ZocaU*84* | P46 | S5 |
| *ZocaU*85* | P9 | S6 |
| *ZocaU*86* | P46 | S6 |
| *ZocaU*87* | P47 | S2 |
| *ZocaU*88* | P48 | S9 |
| *ZocaU*89* | P49 | S10 |
| *ZocaU*90* | P50 | S6 |
| *ZocaU*91* | P51 | S6 |
| *ZocaU*92* | P9 | S6 |
| *ZocaU*93* | P52 | S2 |
| *ZocaU*94* | P53 | S5 |
| *ZocaU*95* | P54 | S5 |
| *ZocaU*96* | P55 | S6 |
| *ZocaU*97* | P5 | S6 |
| *ZocaU*98* | P56 | S1 |
